# Supplementary material for: Applying community health systems lenses to identify determinants of access to surgery among mobile & migrant populations with hydrocele in Zambia: A mixed methods assessment
Source: PLOS Glob Public Health. 2023 Jul 18;3(7):e0002145. doi: 10.1371/journal.pgph.0002145 (PMC10353788; doi:10.1371/journal.pgph.0002145)
Supplement: S1 File — Qualitative and quantitative data collection tools that were used in the study. (DOCX) [file pgph.0002145.s001.docx]

**In-depth Interview guides: Health Providers/Community Health Workers**

Date: Time:

Interviewer: Health provider ID:

Health Facility/District:

Sex: Age:

Education level: Length of stay in the area:

Position: Number of years practicing:

**Barriers to accessibility of MMDP services for migrant and mobile fishing populations**

1. How do hydrocele patients and community members living within this district/health facility catchment area go about accessing services for their condition?
   1. Who do they tend to go to and
   2. What kind of services do they usually receive?
2. Are there specific categories of hydrocele patients who are able to access these services with more ease than others?
3. Which categories are these? Why do you think this is the case?
4. What are some of the reasons that make it difficult for hydrocele patients from fishing and migrant populations to access the health services available within the district? (Probe for knowledge and awareness of existing services, economic reasons, language barriers, socio-cultural beliefs, Discrimination due to nationality, legal requirements in accessing care)

**Community health system barriers to equitable delivery of MMDP services**

1. Within your district/health facility catchment area who is usually involved in providing health services for patients with hydrocele? Are you able to list them (Probe for government organizations, community volunteers, non-governmental organizations, community-based organizations, care givers and local religious /political/traditional leaders)
2. How did these actors become involved in the implementation of health services for hydrocele patients?
   1. Do they have specific roles and responsibilities? If yes, please describe them.
   2. Do they have any influence in how these services are being delivered or utilized within the district?
3. Are local communities, patients and stakeholders able to actively participate in programme activities related to the implementation of health services for hydrocele patients? Why do you think this is the case?
   1. Does this also extend to participation rates for communities and patients from fishing and migrant populations?
   2. Are there any barriers that may inhibit effective participation of fishing and migrant populations? Please describe them?
4. Are there any opportunities for these stakeholders to review the implementation of services for hydrocele and provide their input so as improve the quality and efficiency of services?
   1. What type of opportunities are available? Probe for data review meetings, public forums.
   2. Are such meetings/ forums for providing feedback in the implementation of hydrocele services held regularly? How often are they held?
5. How is the implementation of services for hydrocele within your district funded?
   1. Who provides this funding?
   2. How much funding has been provided for hydrocele services over the past two-three years?
6. What proportion of the funding you receive is used to ensure that the services for hydrocele you provide are able to reach the most marginalised populations?
   1. Does this include communities and patients from fishing and migrant populations?
7. Do patients and their caregivers from fishing and migrant populations incur any costs associated with accessing services for hydrocele? What type of costs are these?
8. Do the existing health facilities cover all regions of the district, making it easy for patients from fishing and migrant populations to access health services? Do you think this is sufficient?
9. Do the health facilities have adequate equipment and supplies to provide services for hydrocele patients from fishing and migrant populations to access health services?
   1. Are there referral systems in place to ensure that patients receive the most appropriate care?
   2. What gaps exist in the available infrastructure to provide these services?
10. Are there adequate health care providers/community-based volunteers who are able to provide services for hydrocele patients from fishing and migrant populations?
    1. Have they received any training to do so? What kind of training have they received?
    2. What are the main human resource challenges affecting the provision of services for hydrocele in the district?
11. Do local health facilities/community health workers utilize any guidelines or manuals to help them implement services for hydrocele services? If yes please describe them.
    1. How do they ensure that the practices in these guidelines are being followed? (Probe for supervision)
12. Are local health care providers or community health workers provided with any incentives to improve their performance when implementing services for hydrocele ? If yes please describe the incentives that they receive.
13. Do you collect any data on how hydrocele services are being utilized within your district/health facility catchment area?
    1. Please describe your role in the collection of this data.
    2. Is there an effective monitoring and feedback mechanism in place to help you collect the relevant information?
14. How would you describe the quality of the data that you collect?
    1. Is it often analyzed and used to inform the delivery of hydrocele services ?
    2. Have you been able to use the data to identify communities that are not receiving hydrocele services?
    3. To what extent do you feel that the data you collect is able to inform you on the needs of the communities?

**Impact of COVID-19 on the delivery and integration of MMDP**

1. Has COVID-19 affected the extent to which you are implementing hydrocele services within the district/health facility catchment area?
   1. Please describe the ways in which it has affected your activities? Probe for both positive and negative effects on surgical procedures, suspension of programme activities, multisectoral collaboration etc.
   2. Has it had any impacts on service delivery that are specific to fishermen and migrant populations in Luangwa? Please describe them.
2. What measures are being put in place to address these challenges?
   1. Are there any measures put in place to improve service delivery to fishermen and migrant populations in Luangwa? Please describe them.
   2. How can we prevent and address these challenges in the future?

**Potential strategies to improve the uptake and sustainability of MMDP services**

1. What recommendations would you suggest to help improve the implementation of hydrocele services?
   1. Do you have any suggestions that can address challenges that are specific to hydrocele patients from fishing and migrant populations? (Probe for Community level recommendations. Facility level recommendations, local political structures etc)
2. What recommendations do you have for integrating services for hydrocele patients into community based health systems?
3. Community level recommendations; community health workers, patients, community members
4. Facility level recommendations
5. Provincial level recommendations
6. National level recommendations

**Interview guide: Community Leaders**

Date: Time:

Interviewer: Interview ID:

Health Facility Catchment Area:

**Barriers to accessibility of MMDP services for migrant and mobile fishing populations**

1. How do hydrocele patients and community members living within this district/health facility catchment area go about accessing services for their condition?
   1. Who do they tend to go to and what kind of services do they usually receive?
2. What MMDP services are commonly used by hydrocele patients and community members?
3. Are there specific categories of hydrocele patients who are able to access these services with more ease than others?
   1. Which categories are these? Why do you think this is the case?
4. What are some of the reasons that make it difficult for hydrocele patients from fishing and migrant populations to access the MMDP services available within the district? (Probe for knowledge and awareness of existing services, economic reasons, language barriers, socio-cultural beliefs, Discrimination due to nationality, legal requirements in accessing care)

**Community health system barriers to equitable delivery of MMDP**

1. **Governance**
2. Within your community, who is usually involved in providing MMDP interventions for patients with hydrocele? Are you able to list them (Probe for government organizations, community volunteers, non-governmental organizations, community-based organizations, care givers and local religious /political leaders)
3. Ate you or any community members involved in the implementation of these MMDP services?
   1. Do you have any specific roles and responsibilities? If yes, please describe them.
   2. How did you or other community members become involved?
   3. Do you feel that you or other community members have an influence on how these services are implemented? Why do think that this is the case?
4. Are you or any community members able to actively participate in programme activities related to the implementation of MMDP services? Why do you think this is the case?
   1. Is this for communities and patients from fishing and migrant populations?
   2. Are there any barriers that may inhibit effective participation? Please describe them?
5. Do you feel satisfied with the quality of MMDP services that are being implemented in your community? Why would you say this is the case?
6. Are there any opportunities for you or any community members to review the implementation of MMDP services and provide your input so as improve the quality and efficiency pf services?
   1. What type of opportunities are available? Probe for data review meetings, public forums.
   2. Are such meetings/ forums for providing feedback in the implementation of MMDP services held regularly? How often are they held?
   3. What are some of the challenges that may make it difficult for you to provide your input?
7. **Financing**
8. Do hydrocele patients and their caregivers incur any costs associated with accessing MMDP services? What type of costs are these?
9. **Service delivery**
10. Do existing health facilities cover all regions of the district, making it easy for patients from your community to access MMDP services?
    1. Do you think this also applies to fishing and migrant populations to access health services? Why do you think this is the case?
11. Do the health facilities have adequate equipment and supplies to provide MMDP services?
12. Are there referral systems in place to ensure that patients receive the most appropriate care?
13. What gaps exist in the available infrastructure to provide these services?
14. Are there adequate health care providers/community-based volunteers who are able to provide MMDP services to patients?
15. Do you feel like they are well equipped to provide these services?
16. **Information**
17. Have you or other community members ever been asked to provide information that can be used to improve the implementation of MMDP services within your community?
    1. What kind of information were you asked to provide? Please describe.
    2. Do you feel as though your suggestions have been used to improve the programme?

**Impact of COVID-19 on the delivery and integration of MMDP**

1. Has COVID-19 affected the extent to which hydrocele patients who are living in your community have been able to access MMDP interventions over the past few months?
   1. What challenges are they facing in accessing services? Please describe them.
   2. Are challenges that make it especially difficult for hydrocele patients who are fishermen and from migrant populations? Please describe them.
2. Have you seen any measures being used by health workers and community-based volunteers to provide MMDP interventions in a manner that addresses these challenges? If yes, what measures are these
   1. Are of these measures targeted towards fishermen and mobile populations in Luangwa? Please describe them.
   2. How can we mitigate these challenges in the future?

**Potential strategies to improve the uptake and sustainability of MMDP services**

1. What recommendations would you suggest to help improve the implementation of MMDP services? Probe for Community level recommendations. Facility level recommendations, local political structures etc
   1. Do you have any suggestions that can address challenges that are specific to hydrocele patients from fishing and migrant populations?
2. What recommendations do you have for integrating LF morbidity services into community health systems?
3. Community level recommendations; community health workers
4. Facility level recommendations
5. Provincial level recommendations
6. National level recommendations

**Interview guide: Hydrocele patients from migrant and mobile populations**

Date: Time:

Interviewer: Interview ID:

Health Facility/District: Population category:

Sex: Age:

Education level: Length of stay in the area:

**Introduction**

1. Please tell us a bit about yourself. (Probe for How long have you lived in the area, when did they find out that they had hydrocele and how long have they had the condition.)
2. How would you describe the state of your health? Ask the participant whether they feel like the state of their health is excellent, good or poor.
3. Has having hydrocele affected the level in which you are able to; (Probe for exploration)
   1. Work or perform normal activities around the house.
   2. Ability to spend time with your family or friends.
   3. Move freely within your community including visiting public places such as churches, schools or the market.
   4. The extent to which you are able to take part in community activities e.g. community groups.
4. Have you ever felt depressed, sad or hopeless within the past few months?
   1. Did these feelings come about because of your condition?
5. Have you sought any help from your family, friends or health care providers to help you deal with these feelings?
   1. For those who went to the health care provider, what kind of services did you access?

**Barriers to accessibility of MMDP services for migrant and mobile fishing populations**

1. How do you usually go about accessing services for your condition?
   1. Who do you tend to go to and what kind of services do you usually receive?
2. What MMDP services do you commonly use?
3. Are there specific categories of hydrocele patients who are able to access these services with more ease than others?
   1. Which categories are these? Why do you think this is the case?
4. What are some of the reasons that make it difficult for you, being a patient from a fishing and/or migrant population to access the MMDP services available within the district? (Probe for knowledge and awareness of existing services, economic reasons, language barriers, socio-cultural beliefs, Discrimination due to nationality, legal requirements in accessing care)

**Community health system barriers to equitable delivery of MMDP services**

1. **Governance**
2. Within your district/health facility catchment area who is usually involved in providing MMDP interventions for patients with hydrocele? Are you able to list them (Probe for government organizations, community volunteers, non-governmental organizations, community-based organizations, care givers and local religious /political leaders)
3. Ate you involved in the implementation of these MMDP services?
   1. Do you have any specific roles and responsibilities you play? If yes, please describe them. How did you become involved?
   2. Do you feel that you or other patients from fishing or migrant communities have an influence on how these services are implemented? Why do think that this is the case?
4. Are you or any community members able to actively participate in programme activities related to the implementation of MMDP services? Why do you think this is the case?
   1. Is this for also the communities and patients from fishing and migrant populations?
   2. Are there any barriers that may inhibit effective participation? Please describe them?
   3. Do you have any suggestions on how you can be involved in implementation of these services?
5. Do you feel satisfied with the quality of MMDP services that are being implemented in your community? Why would you say this is the case?
6. Are there any opportunities for you or any community members to review the implementation of MMDP services and provide your input so as improve the quality and efficiency pf services?
   1. What type of opportunities are available? Probe for data review meetings, public forums.
   2. Are such meetings/ forums for providing feedback in the implementation of MMDP services held regularly? How often are they held?
   3. What are some of the challenges that may make it difficult for you to provide your input?
7. **Financing**
8. Do hydrocele patients and their caregivers incur any costs associated with accessing MMDP services? What type of costs are these?
9. **Service delivery**
10. Do existing health facilities cover all regions of the district, making it easy for patients from your community to access MMDP services?
    1. Is it easy for you as a patient from a fishing and/or migrant population to access health services? Why do you think this is the case?
11. Do the health facilities have adequate equipment and supplies to provide MMDP services?
12. Are there referral systems in place to ensure that patients like you receive the most appropriate care?
13. What gaps exist in the available infrastructure to provide these services?
14. Are there adequate health care providers/community-based volunteers who are able to provide MMDP services to patients?
15. Do you feel like they are well equipped to provide these services?
16. **Information**
17. Have you ever been asked to provide information that can be used to improve the implementation of MMDP services within your community?
    1. What kind of information were you asked to provide? Please describe.
    2. Do you feel as though your suggestions have been used to improve the programme?

**Impact of COVID-19 on the delivery and integration of MMDP**

1. Has COVID-19 affected the extent to which, you as a hydrocele patient are able to access MMDP interventions over the past few months?
   1. What challenges are you facing in accessing services? Please describe them.
   2. Do you think these challenges are because you are a fishermen or from migrant population? Why do you think this is the case?
2. Have you seen any measures being used by health workers and community-based volunteers to ensure that you are able to receive these services? If yes, what measures are these
   1. How can we mitigate these challenges in the future?

**Potential strategies to improve the uptake and sustainability of MMDP services**

1. What recommendations would you suggest to help improve the implementation of MMDP services? Probe for Community level recommendations. Facility level recommendations, local political structures etc
2. Do you have any suggestions that can address challenges that are specific to hydrocele patients from fishing and migrant populations?
3. What recommendations do you have for integrating LF morbidity services into community health systems?
4. Community level recommendations; community health workers
5. Facility level recommendations
6. Provincial level recommendations
7. National level recommendations

**Patient survey questionnaire**

Date: Time:

Interviewer: Translator:

Village: Health Facility Catchment Area:

Distance to nearest Health Facility: Patient ID number:

| **No.** | **Question** | | | | | | | | **Code** | | | **Response** | | |
| --- | --- | --- | --- | --- | --- | --- | --- | --- | --- | --- | --- | --- | --- | --- |
| **SECTION 1: DEMOGRAPHIC INFORMATION** | | | | | | | | | | | | | | |
| 1 | What is your date of birth? | | | | | | | |  | | |  | | |
| 2 | How old are you? | | | | | | | |  | | |  | | |
| 3 | What is your marital status?  Single  Married  Divorced/Separated  Widowed  Cohabiting  Have other wives | | | | | | | | 1  2  3  4  5  6 | | |  | | |
| 4 | What is the highest level of education you have attained?  No education  Primary School  Junior High School  Secondary School  Vocational training  University/College  Other: (Specify) | | | | | | | | 1  2  3  4  5  6  7 | | |  | | |
| 5 | Do you have any disability other than hydrocele or lymphoedema?  Yes  No | | | | | | | | 1  2 | | |  | | |
| 6 | If you have a disability, what kind of disability is it? (Tick all that apply)  Hearing  Seeing  Speaking  Mental  Physical/walking/Limbs  Other (Specify) | | | | | | | | 1  2  3  4  5  6 | | |  | | |
| 7 | What is your country of origin?  Zambia  Zimbabwe  Mozambique  Other (Specify) | | | | | | | | 1  2  3  4 | | |  | | |
| 8 | Do you travel across the borders seasonally? (Migrant)  Yes  No | | | | | | | | 1  2 | | |  | | |
| 9 | If yes, how often do you travel? | | | | | | | |  | | |  | | |
| 10 | When you travel how long do you stay away from home? | | | | | | | |  | | |  | | |
| 11 | If yes and they travel often, what are the main reasons for your movement?  Fishing  Farming  To see family  To conduct trading activities  For school  Other (Specify) | | | | | | | | 1  2  3  4  5  6 | | |  | | |
| 12 | Do you have a permanent residence in Luangwa?  Yes  No | | | | | | | | 1  2 | | |  | | |
| 13 | If yes, how long have you lived here? | | | | | | | |  | | |  | | |
| 14 | What is your main economic activity?  Employed  Looking for a job  Household work  Does not work | | | | | | | | 1  2  3  4 | | |  | | |
| 15 | For those who are employed, what is your primary source of income?  Farming  Fishing  Day worker (e.g. factory, construction)  Small scale enterprise/ self-employed (e.g. kiosk owner, market owner)  Private sector employment (e.g. banking, business, clerical)  Civil servant/ Government official  Other: (Specify) | | | | | | | | 1  2  3  4  5  6  7 | | |  | | |
| 16 | What is your average income per month? Respondent should state the actual amount. | | | | | | | |  | | |  | | |
| **SECTION 2: HYDROCELE MANAGEMENT** | | | | | | | | | | | | | | |
| 17 | How long have you had Hydrocele? | | | | | | | |  | | |  | | |
| 18 | Does the swelling appear bigger on one side or is it evenly spread out?  One side (unilateral)  All sides are swollen to the same degree (bilateral) | | | | | | | | 1  2 | | |  | | |
| 19 | Do you have hydrocele on the left side, right sides or both sides?  Left sides  Right sides  Both sides | | | | | | | | 1  2  3 | | |  | | |
| 20 | Are you aware of any health care services that can help treat your condition?  Yes  No | | | | | | | | 1  2 | | |  | | |
| 21 | If yes, how did you find out about the existence of these services?  Family members  Friends  Community meetings  Community health worker/Health care provider  Radio, TV or social media  Community leaders  Other (Specify) | | | | | | | | 1  2  3  4  5  6  7 | | |  | | |
| 22 | Have you ever sought medical help for your condition from a health facility in the past 12 months?  Yes  No | | | | | | | | 1  2 | | |  | | |
| 23 | If yes, how many times have you visited the health facility for your condition in the past 12 months? | | | | | | | |  | | |  | | |
| 24 | What type of services did you receive?  Surgery  Draining of fluid  Doxycycline  Referral to another facility  Did not receive any form of care  Other specify | | | | | | | | 1  2  3  4  5 | | |  | | |
| 25 | Have you ever sought medical help for your condition from a traditional healer in the past 12 months?  Yes  No | | | | | | | | 1  2 | | |  | | |
| 26 | If yes, how many times have you visited the traditional healer in the past 12 months for your condition? | | | | | | | |  | | |  | | |
| 27 | In general, would you say that your health is  Excellent  Very good  Good  Fair  Poor | | | | | | | | 1  2  3  4  5 | | |  | | |
| 28 | During the past 30 days, how often would you say you have felt  Nervous  Hopeless  Restless or fidgety  So, depressed nothing could cheer you up  That everything was an effort  Worthless | | **All the time** | | **Most of the time** | | **Some of the time** | | | **A little of the time** | | | **None of the time** | |
|  |  |  | 1 | | 2 | | 3 | | | 4 | | | 5 | |
|  |  |  | 1 | | 2 | | 3 | | | 4 | | | 5 | |
|  |  |  | 1 | | 2 | | 3 | | | 4 | | | 5 | |
|  |  |  | 1 | | 2 | | 3 | | | 4 | | | 5 | |
|  |  |  | 1 | | 2 | | 3 | | | 4 | | | 5 | |
|  |  |  | 1 | | 2 | | 3 | | | 4 | | | 5 | |
| 29 | If you consider your responses from the previous questions, how often did these feelings come up?  More often than usual  About the same as usual  Less often than usual  Never had any of these feelings | | | | | | | | 1  2  3  4 | | |  | | |
| 30 | During the past 30 days, how many days out of the 30 days were you totally unable to work or carry out your normal activities because of your feelings? **(List number of days)** | | | | | | | |  | | |  | | |
| 31 | Not counting the days you have mentioned in the question above, how many days were you able to do only half or less of what you would normally have been able to do because of these feelings? **(List number of days)** | | | | | | | |  | | |  | | |
| 32 | During the past 30 days, how many times did you see a doctor because of these feelings? **(List number of times)** | | | | | | | |  | | |  | | |
| 33 | During the past 30 days, how often has hydrocele been the main cause of these feelings  All the time  Most of the time  Some of the time  A little of the time  None of the time | | | | | | | | 1  2  3  4  5 | | |  | | |
| 34 | Do you have any other health challenge that you would like to share?  Yes  No | | | | | | | | 1  2 | | |  | | |
| 35 | If yes please state what health challenge you have? | | | | | | | |  | | |  | | |
| **SECTION 4: INDIVIDUAL AND COMMUNITY HEALTH SYSTEM BARRIERS AFFECTING EASE OF ACCESSING SERVICES** | | | | | | | | | | | | | | |
| 36 | Do you face any problems/challenges in accessing health services for hydrocele?  Yes  No | | | | | | | | 1  2 | | |  | | |
| Next questions to be filled out by those who have answered yes to the previous question. | | | | | | | | | | | | | | |
|  | **Availability and access of services** | | | | | | | |  | | |  | | |
| 37 | Do you know where to get services to help treat your condition?  Yes  No | | | | | | | | 1  2 | | |  | | |
| 38 | Are there any health facilities close to you from where you can easily access health services?  Yes  No | | | | | | | | 1  2 | | |  | | |
| 39 | If there are none, where do you usually go to receive medical assistance? | | | | | | | |  | | |  | | |
| 40 | How far is this health facility from where you live? (List in kilometres) | | | | | | | |  | | |  | | |
| 41 | Are you able to receive care at these health facilities at any time both day and night?  Yes  No | | | | | | | | 1  2 | | |  | | |
| 42 | Is the health facility closest to you able provide the services needed to treat your condition?  Yes  No | | | | | | | | 1  2 | | |  | | |
| 43 | If not, have they given you a referral to another facility to receive these services?  Yes  No | | | | | | | | 1  2 | | |  | | |
| 44 | For Migrants and fishermen, during the periods when you have travelled are you still able to access health facilities?  Yes  No | | | | | | | | 1  2 | | |  | | |
| 45 | If not, how do you usually access health services for your condition | | | | | | | |  | | |  | | |
| 47 | Are you required to present any documents to access the services for your condition?  Yes  No | | | | | | | | 1  2 | | |  | | |
|  | **Inclusive service delivery:** | | | | | | | |  | | |  | | |
| 48 | Is it difficult to communicate your symptoms to health care providers and community-based volunteers? **(language barriers)**  Yes  No | | | | | | | | 1  2 | | |  | | |
| 49 | Have you ever felt afraid to go to the health facility because you are afraid you will face stigma and judgement for your condition?  Yes  No | | | | | | | | 1  2 | | |  | | |
| 50 | Do you face discrimination from health care providers and community-based volunteers when you go to the health facility to access services for your condition?  Yes  No | | | | | | | | 1  2 | | |  | | |
| **51** | Are the services and materials used by health providers/community health workers culturally sensitive?  Yes  No | | | | | | | | 1  2 | | |  | | |
| **52** | Have you ever been denied services at the health facility for your condition?  Yes  No | | | | | | | | 1  2 | | |  | | |
| **53** | What reason was provided for the refusal to provide you with services? | | | | | | | |  | | |  | | |
| 54 | Are there any cultural and social beliefs that prevent you from accessing health services for your condition?  Yes  No | | | | | | | | 1  2 | | |  | | |
| 55 | If yes, please specify which ones | | | | | | | |  | | |  | | |
|  | **Affordability** | | | | | | | |  | | |  | | |
| 56 | Do you incur any costs in order to access health services for your condition?  Yes  No | | | | | | | | 1  2 | | |  | | |
| 57 | What type of costs do you incur when accessing health services for your condition? | | | | | | | |  | | |  | | |
| 58 | If yes, are you able to afford these costs?  Yes  No | | | | | | | | 1  2 | | |  | | |
| 59 | Do you consider these costs to be a challenge to your ability to access health services for your condition?  Yes  No | | | | | | | | 1  2 | | |  | | |
|  | **Community health system actors** | | | | | | | |  | | |  | | |
| 60 | Who are the main actors in the delivery of health services for your community and how would you rate their involvement  Health facility staff  Community health workers  Community leaders e.g. chiefs and headmen  Religious leaders  Traditional healers  Neighbourhood Health Committee  Community based groups | **Highly**  **Involved in all activities** | | **Involved in most activities** | | | **Involved some of the**  **times** | | | **Minimal involvement** | | | | **Not involved**  **At all** |
|  |  |  | |  | | |  | | |  | | | |  |
|  |  |  | |  | | |  | | |  | | | |  |
|  |  |  | |  | | |  | | |  | | | |  |
|  |  |  | |  | | |  | | |  | | | |  |
|  |  |  | |  | | |  | | |  | | | |  |
|  |  |  | |  | | |  | | |  | | | |  |
|  |  |  | |  | | |  | | |  | | | |  |
| 62 | How would you describe the level to which they work together to implement health services for your condition?  They work very well together  They work together but not very well  They do not work together at all  I don’t know | | | | | | | | 1  2  3  4 | | |  | | |
|  | **Trust and confidence in community health systems** | | | | | | | |  | | |  | | |
| 63 | How confident are you in the community actors involved in providing health services for your condition?  Health facility staff  Community health workers  Community leaders e.g. chiefs and headmen  Religious leaders  Traditional healers  Neighbourhood Health Committee  Community based groups | | | | **Very confident** | **Confident** | | **Fairly confident** | | | **Little confidence** | | | **No confidence at all** |
|  |  |  |  |  |  |  | |  | | |  | | |  |
|  |  |  |  |  |  |  | |  | | |  | | |  |
|  |  |  |  |  |  |  | |  | | |  | | |  |
|  |  |  |  |  |  |  | |  | | |  | | |  |
|  |  |  |  |  |  |  | |  | | |  | | |  |
|  |  |  |  |  |  |  | |  | | |  | | |  |
|  |  |  |  |  |  |  | |  | | |  | | |  |
| 64 | How much do you trust that?  When you go to the facility you will be taken seriously  That you will receive enough attention and time  That Health care providers and community health workers have adequate information about your condition  You will be given enough information about your condition  You will be provided with the most appropriate care | | | | **A lot** | **Quite a lot** | | **little** | | | **Very little** | | | **No trust at all** |
|  |  |  |  |  |  |  | |  | | |  | | |  |
|  |  |  |  |  |  |  | |  | | |  | | |  |
|  |  |  |  |  |  |  | |  | | |  | | |  |
|  |  |  |  |  |  |  | |  | | |  | | |  |
|  |  |  |  |  |  |  | |  | | |  | | |  |
|  | **Participation and social exclusion** | | | | | | | |  | | |  | | |
| 65 | Have you ever been involved in the provision of services for your condition?  Yes  No | | | | | | | | 1  2 | | |  | | |
| 66 | If yes, in what aspects of the implementation were you involved in?  Planning  Contribution of resources  Implementation e.g. awareness raising  Monitoring and Evaluation | | | | | | | | 1  2  3  4 | | |  | | |
| 67 | Do you feel that your condition affects the level to which you are able to be involved in these activities including accessing health services?  Yes  No | | | | | | | | 1  2 | | |  | | |
| 68 | Are there any other challenges you have faced in accessing services for your condition?  Yes  No | | | | | | | | 1  2 | | |  | | |
| 70 | Thank you for your participation, would you have any other comments to add on the subject matter, the interview or general health in your area. | | | | | | | |  | | |  | | |
