# Supplementary material for: Applying community health systems lenses to identify determinants of access to surgery among mobile & migrant populations with hydrocele in Zambia: A mixed methods assessment
Source: PLOS Glob Public Health. 2023 Jul 18;3(7):e0002145. doi: 10.1371/journal.pgph.0002145 (PMC10353788; doi:10.1371/journal.pgph.0002145)
Supplement: S2 File — Filled out reporting guideline appropriate for a mixed methods study. (DOCX) [file pgph.0002145.s002.docx]

**Good Reporting of A Mixed Methods Study (GRAMMS)**

| **Guideline** | **Section: page** |
| --- | --- |
| Describe the justification for using a mixed methods approach to the research question | Methods: Page 5 |
| Describe the design in terms of the purpose, priority and sequence of methods | Methods: Page 5 and 8 |
| Describe each method in terms of sampling, data collection and analysis | Methods: pages 6-8 |
| Describe where integration has occurred, how it has occurred and who has participated in it | Methods: pages 8 |
| Describe any limitation of one method associated with the present of the other method | Methods: pages 8 |
| Describe any insights gained from mixing or integrating methods | Methods: pages 8 |
