# Supplementary material for: Applying community health systems lenses to identify determinants of access to surgery among mobile & migrant populations with hydrocele in Zambia: A mixed methods assessment
Source: PLOS Glob Public Health. 2023 Jul 18;3(7):e0002145. doi: 10.1371/journal.pgph.0002145 (PMC10353788; doi:10.1371/journal.pgph.0002145)
Supplement: S3 File — Data collected and reported in the manuscript. (ZIP) [file pgph.0002145.s003.zip › S2. Datasets/Hydrocele Managment practices.docx]

Files\\COMMUNITY HEALTH WORKER 1 - § 1 reference coded [ 6.52% Coverage]

Reference 1 - 6.52% Coverage

I= Yes people who suffer from hydrocele, how do they find help, how does it go?
R= Those who have this disease of hydrocele there are some people who go throughout the community to give medicine.
I= What program is that?
R= The program that they give medicine concerning the same disease because this disease enters through a mosquito bites
I= Okay
R= So they give medicine for that disease if is in the body it should die and those people if they are found with this disease they have to be operated on from the hospital.
I= Where do these people go when they know that they have this disease of hydrocele, who do they go to?
R= As volunteers we go through the community to find out those who are suffering from this disease then we come with them and talk to those who operates of the hospital then we take them there.
I= Okay, how do you find out those people who are sick?
R= We do find them by going in the community door to door, because we usually have some meetings, where you are able to identify and sometimes you can just see the way they are looking and you conclude to say this hydrocele.
I = Just by looking
R= Yes
I= How do they look like
R= This disease the tentacle becomes big and you are able to tell.

Files\\Head Clinical Care LDH - § 2 references coded [ 3.67% Coverage]

Reference 1 - 2.25% Coverage

I: I want an overall overview of how hydrocele patients or cases are here in Luangwa.
R: From the time I came, I have noticed that the cases of hydrocele are many and coming from different parts of the district, some come from Mpuka, some from Kakalo and others from Zimbabwe. The number has been quite high because from the time I came, I have operated on more than 50 patients. So the prevalence of hydrocele is high. Even now in the ward they are 2 for hydrocele those that were operated yesterday.

Reference 2 - 1.42% Coverage

I: Who mostly receive services on hydrocele, Malawian, Zimbabweans or those from Mozambique?
R: On average I would rate them as Zambia, Mozambique and Zimbabwe. But for Zimbabweans, the percentage is lower, the bigger chunk comes from Zambians and Mozambicans. Zimbabwe, less than 2%. These are migrants.
