# Supplementary material for: Applying community health systems lenses to identify determinants of access to surgery among mobile & migrant populations with hydrocele in Zambia: A mixed methods assessment
Source: PLOS Glob Public Health. 2023 Jul 18;3(7):e0002145. doi: 10.1371/journal.pgph.0002145 (PMC10353788; doi:10.1371/journal.pgph.0002145)
Supplement: S3 File — Data collected and reported in the manuscript. (ZIP) [file pgph.0002145.s003.zip › S2. Datasets/Mental Health related to hydrocele.docx]

Files\\FISHERMAN - § 1 reference coded [ 5.71% Coverage]

Reference 1 - 5.71% Coverage

I = How did you feel when you knew about your disease?
R = I tried to use the traditional medicine but it never worked out
I = it worked out?
R =Yes it failed and my heart felt very bad.
I = why?
R = because imagin you never had the items but you see them becoming big, my heart felt bad.
I = you felt bad?
R = yes, it was bad because things that you never had.
I = did you go somewhere to explain how you were feeling?
R = No.
I = no?
R= yes, I was just at home and going to the clinic.

Files\\IDI - Patient - Kansinsa - § 1 reference coded [ 2.88% Coverage]

Reference 1 - 2.88% Coverage

I: Have ever felt depressed, sad or hopeless within the past few months for whatever reasons?
R: No. Getting sad comes when we have argued with someone not on my own I get sad or depressed never.
I: Don’t you hopeless as a result of having this condition?
R: No, I just feel good.
I: How do you usually go about accessing services for your condition?
R: Like I said I have only been to the clinic once to see Tony and that is he solved my problem last year and since then I have never been here.

Files\\IDI - Patient - Mpuka 2 - § 1 reference coded [ 8.85% Coverage]

Reference 1 - 8.85% Coverage

I: Has having hydrocele affected your ability to work or perform your daily activities around the house?
R: If the condition is just normal I do manage to do any work but if it has starts being painful, I don’t do any work.
I: You even fail to catch fish?
R: Yes since peddling and putting the net requires power which I usually don’t have because of the pain.
I: What of the ability to spend time with your friends and family? Do you manage to spend time with your family and family like you used to before having this condition?
R: I do manage to be with them and have come chat but when I feel the pain I stay at home and my friends even ask me that why do I look low these days and I am not usually found with them. Meaning that I spend less time with them.
I: Has this condition also affected you in moving freely within your community like going to the market, schools or church?
R: Whenever the condition is critical I do not go there, not even going for fishing. I go to the clinic to get the drugs and come to stay home waiting for it to normalize. Then after a month when I feel like moving I can do that.
I: What about the community works or activities, do you manage taking part?
R: If I am feeling well I do participate but if I am not ok, I don’t.
I: So meaning when you compare the way you used to participate in activities and now.
R: I don’t take part in a lot of activities like I used to previous, I miss many of them.

Files\\IDI - Patient - Sinyawagora - § 2 references coded [ 19.63% Coverage]

Reference 1 - 10.84% Coverage

I: So in short you are saying you are not very good?
R: Yes my status is not very good especially that I am not working the way I used to in the past.
I: So since you are saying you cannot work the same you way you used to in the past? What type of works are you failing, is farming or house chores or what?
R: No. Because some times when you stay at the same point for some time it will start paining, and even when you walk for a long distance that means you have invited the pain.
I: Do you manage to go to places like the church, at school or at the market?
R: Since the church is near I do manage to go. But going to the market from the time got I have not tried to move that far on foot. I do discourage myself walking that long distance because of the pain I experience.
I: What of in terms of the time spend with your family or friends, has it reduced or it is the same as a result of having hydrocele?
R: With my friends, it is ok, it doesn’t mean that when you feel something paining you need to change the way of living, there is no problem being with them.
I: I mean the length of time spent with your friends?
R: The time I spend with my friends has reduced, because sometimes you find that while you are with your friends, you start feeling the pain, then you excuse yourself and go home to rest.
I: What about the time you spend with your family?
R: My family is now aware, when I feel the pain, I just tell my wife that I am not feeling well and she knows that it is the hydrocele pain.
I: When it comes to taking part in community work, do you participate the same way you used to previously before having this condition?
R: I have not taken part in any community works here because I am usually not around.

Reference 2 - 8.78% Coverage

I: In the past few months, have you ever felt depressed, sad or hopeless?
R: Yeah there are times when I feel like that. I sometimes ask myself questions why I get angry or sad as if there is someone who has made me to upset or there is something bad. This usually happens.
I: When you look at all these, do you feel these feelings come because of your condition?
R: I try to ask about this but I fail to find the answer.
I: But before you had condition, were you still feeling depressed, sad or hopeless?
R: The beginning of this condition I thought it was a joke, I was thinking maybe I didn’t just move properly or I didn’t sit well because it would pain and stop for several times until it grew big on the left side.
I: So when you were feeling depressed, sad or hopeless, did you sought any help from your family friends or health workers to deal with these feelings?
R: I tried to seek for help from my family because as a family, when you meet problem you share with your family first.
I: Since you told a family, how were you helped?
R: The way I looked at that, the person I asked for help from is someone you can think he can fail to heal any disease but on this very one he tried here and there but he failed. But when I met this man who was going round in my community and shared with him what I am going through, he advised me to go to the hospital and seek medical attention.
