# Supplementary material for: Applying community health systems lenses to identify determinants of access to surgery among mobile & migrant populations with hydrocele in Zambia: A mixed methods assessment
Source: PLOS Glob Public Health. 2023 Jul 18;3(7):e0002145. doi: 10.1371/journal.pgph.0002145 (PMC10353788; doi:10.1371/journal.pgph.0002145)
Supplement: S3 File — Data collected and reported in the manuscript. (ZIP) [file pgph.0002145.s003.zip › S2. Datasets/Potential implementation strategies.docx]

Files\\Head Clinical Care LDH - § 1 reference coded [ 15.19% Coverage]

Reference 1 - 15.19% Coverage

I: Okay, let us talk about the recommendations on how we can improve the hydrocele services in the district. So will talk about community, local political structures and facility levels. How can the challenges be addressed to improve the issue of hydrocele especially for fishermen and migrants?
R: With the community, there is an area where the fishermen always start from before they go and fish and they have an association, so if health education can be given to them on what is, what causes and what are the benefits of hydrocele can be given to them, it can help a lot. Because once we involve people, they will not be surprised if there are some success stories, we can also take to them for them to learn. And it is very easy for anyone to disseminate information to them at the harbour since that is where most of the fishermen are found. You talk to them, they understand the issues and complication and a lot will come, that is at community level. Then at health centre level, people have just been seeing hydrocele as a disease form the books. So the district office can do a clinical visit on these centres to sensitize on hydrocele, this can help a lot during their monthly visits at these centres also put hydrocele as one of the health problems in the district.
I: Any recommendations at local political structures?
R: Those have to be involved because if there is political will, then that is when things can run. If it is the Councillor, town Chairperson is well informed because these are the people who the people on the ground and if the information may come from civic leaders, it may be easy for people follow what they always say. So these are the key people to tackle so that when they come they will talk to the district and the district will take it up.
I: As we conclude, we can talk about how we can integrate services for hydrocele patients into the community health systems.
R: I think for me, integration of hydrocele at community is very simple undertaking, because there are those programmes that run like MCH, oral vitamin A, those are the ones that always run I think monthly. So what the district can do is to incorporate public health officers to talk about hydrocele into the community, if it is a programme that is been held consistently, it will be very easy for us to identify those people that have this condition in the community and allocating a small portion of money to the PR for the same communities. Because that is what is happening with the eye clinic, like for them, they start with community leaders and they go to talk to the people through the sensitization and most people come for eye check-ups and that can be the same with hydrocele and we can save a lot of people.
I: How can we integrate at facility level?
R: At facility level, they just have to continue talking about this hydrocele during their child health week and community meetings through the headmen and community leaders.
I: What about recommendations on how we can integrate these health systems at provincial level?
R: I think it is very easy because the problem which is there is that the condition is not very prevalent in most of the district, so now since it is prevalent, the province can integrate and give a small grant for surgical, health education to the affected district because each district has a peculiar disease in their area.

Files\\IDI - CHW - Mangelengele - § 1 reference coded [ 4.78% Coverage]

Reference 1 - 4.78% Coverage

I: So how do we now integrate the programmes of hydrocele services and into community health services, how do we put them so that they work together at community level?
R: When we go in the community, after teaching for antenatal, we can also teach about hydrocele to all of them.
I: And how do we integrate these services for hydrocele and also for the community health systems at facility level like here?
R: Like here at the facility, I can say that if there is any activity at the facility, after that activity, we are supposed to talk about hydrocele, and not keeping quiet and we forget.
I: Alright, any recommendations in terms of integration at provincial and national level?
R: At provincial level, I would like it if they make an effort to communicate with the people in the hospitals so that the expenses can be less for everyone to go and access for help since they are the ones with authority to talk about the bills.

Files\\IDI - Com Leader - Chitope - § 1 reference coded [ 7.98% Coverage]

Reference 1 - 7.98% Coverage

I: As we come to the end of this interview what is it that can be done to integrate hydrocele programs into community health systems? How can they work together?
R: What is needed is for the community health workers, the headmen, nurses, clinical officers and doctors to work together. We try to work hard around hydrocele program and also fuse in Covid19 issues so that it does not make us fail to deliver hydrocele services to people with this problem in the community so that they get the help they need one by one eventually many will access hydrocele services. All is needed is for the people I have mentioned to work together then the hydrocele program will be a success. In addition, let these other clinic programs be combined so that they can be done at once like screening when they go to one area.
I: Thank you so much. We are talking on how we can integrate other health programs at the clinic with hydrocele program. How can we integrate the two?
R: They have to be very strong. For us in the community we tell the person going around the community announcing to emphasize that we all meet at certain place so that when health staff from the clinic or hospital come, people get to be addressed at the same time and get to understand the message.
I: Thank you. I don’t know do you have any question for me sir or any comment?
R: I don’t have a question but I would like to say that hydrocele here in Luangwa district is prevalent. Our fathers used to tell us that hydrocele started a longtime ago. In past people used to say that people with hydrocele are the ones that would infect others without hydrocele while some believed that there is medicine used for someone to have hydrocele whilst others believed that someone with hydrocele cannot be a chief or headman like myself but we now have better understanding that there are mosquitoes in the river which when sucking blooding from the veins they leaves virus that cause hydrocele.

Files\\IDI - Com Leader - M - Kasinsa - § 1 reference coded [ 3.93% Coverage]

Reference 1 - 3.93% Coverage

I: Ok. Thank you so much. How can we integrate hydrocele services with community health systems?
R: We should not have special time for hydrocele patient but they should get to be treated just like malaria patient are treated such that when going for outreach programs even hydrocele topics are covered that way we shall win the game.
I: Why do you say so?
R: It is difficult because it take time when you tell someone about this for them to respond, and that may bring more delays for one to receive the services.
I: Ok. Is there anything you may want to add or even suggestion on what we have discussed?
R: No, I think all has been said already.
I: We are now done with the discussion, do you have a question or comment for me.
R: I don’t have a comment I just want appreciate what we have shared and that should continue.

Files\\IDI - Patient - Kanemela - § 2 references coded [ 7.95% Coverage]

Reference 1 - 3.68% Coverage

I: What measures are being put in place to ensure that the service delivery for hydrocele to fishermen and migrants are improved in Luangwa?
R: I have not seen any measures being put in place regarding us as patients with hydrocele were I can say we are being regarded as real patients even in this Covid era nothing is being done here.
I: Especially in this Covid time?
R: It is not usually done.
I: What can be done for you to receive help fast at the clinic although with your condition you are not supposed to be known to the public?
R: My suggestion is that the health staff need to have data which they can use to know the number of hydrocele patients in their catchment area. So that when they know that such a person has this condition then they quickly attend to him and he goes back.
I: Any other suggestion on how the service delivery can be improved on people with hydrocele even in this Covid period?

Reference 2 - 4.27% Coverage

: What recommendation would you give suggest to help improve the implementation of hydrocele services? Let’s start from community level and then facility level.
R: At community level I would request that the head persons should also be aware of how people in his or her village have got hydrocele. Then they can also see how to help those subjects when they visit the clinic by engaging the health staff. That way they will ensure that we are treated well at the clinic since we will give the report after coming back from the clinic. And also as I said the health staff need data on the number of patients with hydrocele in their catchment area the give those options on how to treat their condition like going for surgery.
I: What recommendation do you have on how we can integrate hydrocele services into community health systems?
R: Since everything is about health, they have to work side by side and creating awareness in communities. And those patients with hydrocele should not be discriminated. This will make us live as one people.

Files\\IDI - Patient - Mpuka 2 - § 1 reference coded [ 12.30% Coverage]

Reference 1 - 12.30% Coverage

I: What recommendation do you have on how we can improve the service delivery for hydrocele?
R: There should be a trained doctor who will deal with hydrocele conditions, enough equipment and the room where to be conducting the surgeries.
I: What recommendation is there on how we can improve the implementation of hydrocele services at community level?
R: Let the Community Health Workers continue to go round in the community and sensitize them on hydrocele and also to register those who have it. With such records and they are submitted to you, then as you come, you can make better plans of how to tackle this. Otherwise, letting those patients to come here on their own no, they can’t show up.
I: Any recommendation on how we can improve the implementation of hydrocele services at facility level apart from what you mentioned?
R: The other recommendation I have is that the doctor to work here should not be from here which people know, otherwise the patients may stop coming since they may be feeling shy because of their condition.
I: Do you have any recommendation at facility level?
R: No, I don’t have.
I: So now what recommendation do you have for integrating hydrocele services into the community health systems at community level? For HIV Aids, TB.
R: Here there are days when people come for example HIV/ Aids and get tested, so when they come on such days, let the people responsible like community volunteers and community health workers also bring in the sensitization on hydrocele and also how the services are delivered at the hospital.
I: What other recommendation do you have on how to integrate the services?
R: We can do that by using the same room to do all the services. But let the boys and men have the same room and the girls and women use the same room so that when you there no one will know that you have gone there for this or that problem since each gender will have its own room and deal with its own services.

Files\\IDI health provider Chitope - § 1 reference coded [ 18.68% Coverage]

Reference 1 - 18.68% Coverage

I: Now since there has not been any activities for hydrocele, how we can improve the implementation of hydrocele to fishermen and migrants at community level?
R: As in other services, what we have been doing, I think instead of monthly visits or other visits we have been giving to the patients, maybe you might give appointment dates to the clients so that they can come to the facility or maybe you can go and see them at an appointed date. So the best is to make appointment date so that you can get in touch with the client.
I: Other suggestions that cane improve the service delivery to fishermen and migrants?
R: Use the Community Health Workers since they are the ones that are found in the community such that if we are overwhelmed with work here, we ask them to follow up on fishermen and migrants especially that our community health workers also do other activities like farming so they go out there across and they can meet the migrants and fishermen there and communicate to them on the information they may need on hydrocele.
I: Any suggestions that can address the challenges that are directed on patients with hydrocele at facility level?
R: Patients are free to come to the facility whenever they see the need and the facility is always ready to welcome them. So as a facility when such a patient comes, because it is not often that they usually come, so when they come, they should be given first priority, so that since they have a known condition which needs to be managed quickly. So in short we are saying, services to hydrocele patients should be prioritized so that these other patients can be encouraged to come and seek for services.
I: How can we integrate hydrocele patients into the community health systems? Let us start with the Community level, how do you integrate these services?
R: There is need for integration of these services into the community by involving the community leaders, because where these people come from they need to feel free and when they are free that can make them be able to disclose their conditions not only to the closest person but possibly even to the headmen and other community leaders and they can be referred to the person who will be of help to such people. If the headman knows that this person has such a condition, they can talk to the community health worker, then that person will refer the client to us. In terms of confidentiality, if the patient does not want people to know about the condition, we have sworn to uphold confidentiality and privacy. So I think we need to do more sensitization to headmen, community leaders, church and traditional leaders and also traditional healers in the community because being a rural area most of them might think the people have been bewitched with such a condition, so I think we also need to talk to the traditional healers so that where they see that these people cannot get healed with such conditions a service can be provided at the clinic. So orientation to the community leaders and Community Health Workers who are the closest people in the Community. So if the community knows that such and such has a condition of hydrocele which can be treated, they might have information on someone who does not want to be treated of such a condition. So, sensitization community health workers and other community leaders can help reach the clients who might want access the needed services.
I: What of at facility level?
R: At facility level, number one we need guidelines, more knowledge and skills on how to manage this because some of these people cannot access these services because of how we have treated them, they might think we do not have the medication or maybe we are not interested in their health but possibly because we do not know how to manage these conditions. The other thing I think again is that we may need to have equipment to help us manage patients, we do not need funds because even when there are no funds, these services will go down but we need skills, knowledge and equipment. As the facility, that will to help clients with these conditions have to be there because we are concentrating more on antenatal, we are very active on that one, so In short, male reproductive services should be prioritized.
I: Any recommendation on how to integrate these services at Provincial and National Level?
R: So, at Provincial and National level, I do not know whether there are already guidelines that have been put on the management of these patients, possibly setting up of clinics starting from the grassroots to the higher levels and putting focal point persons to be in charge of these so that they can be communicating with various departments on the needs to address the services and challenges that these patients are facing because as at now, we do not now the focal point who is in charge of these, probably the only person we know is the environmental officer is the one who deals with such, we do not know who to see, all we know is that this programme falls under environmental health department.

Files\\IDI health provider Mandombe - § 1 reference coded [ 9.53% Coverage]

Reference 1 - 9.53% Coverage

I: In terms of recommendations, what would you suggest that can help improve the hydrocele services for fishermen and the migrant population?
R: I think recommendations in relation to migrants and fishermen, it is a matter of strengthening sensitizations as many as we can. Because people move to and from everytime. So it is a matter of sensitizations on symptoms of hydrocele.
I: That is the recommendation at community level, what about at facility level, what do you suggest can address challenges?
R: The challenges at the facility, it is us we need to make sure we isolate the hydrocele condition and take it serious. We should not wait for MDA for hydrocele, we need to have a time table to make sure we start sensitizing the community, weekly, or twice in a month on how to access services and how to go about it when someone has hydrocele.
I: With regards to the already known hydrocele patients like fishermen, how can you help them?
R: On those people, I don’t know if it can work, it is just a matter of sensitizing them on the entry point, because we cannot follow them to Mozambique where they mostly do their fishing, so if mass sensitization can be done on entry point. But if it is not severe, they just come, do their business and go back.
I: Now, I want to get a recommendation on integrating these hydrocele services into the community based health systems? How can we integrate these services for hydrocele into community based systems at community level?
R: I think the services can be integrated, for instance if we can create a programme for hydrocele where we say, I think most of the things are getting back to creating specific time for these conditions. Because for hydrocele, it is different from Malaria and we have underrated the condition. For example, on Covid, it is the programme that has been taken so serious through sensitizations in communities, banners, radio stations too. So if hydrocele can be sensitized like Malaria, it can help, if it means financial support, it can also help.
I: What about the recommendation on how we can integrate hydrocele services into community based health systems at facility level?
R: I think at facility level, we can integrate it at the entry point at OPD, we need to start screening everyone maybe, putting a general screening, because some people cannot open up, so we can only integrate it if we put it as part of screening and we ask questions to whoever comes in, it can help us to people who fail to open up.

Files\\IDI_ Health Provider Kasinsa - § 1 reference coded [ 12.79% Coverage]

Reference 1 - 12.79% Coverage

I: Do you have any recommendation that can help address the challenges with regards to hydrocele patients from fishing camps and migrant populations at community level and facility level?
R: At community level, I think it’s important to have more focal point people for hydrocele because there is only one. Because we have 3 NHC, so at least 1 person per NHC to provide sensitizations on what services do people with hydrocele receive at the facility. Then at facility level, staff members need to be oriented on these cases, some don’t know how to differentiate if it is arthritis or hydrocele, so we need more sensitization on issues of hydrocele and the treatment options that clients can have because others might think you only give patients anti biotics.
I: Any recommendations to do with local political structures or leaders?
R: For us, headmen are the most important ones, if we want the programme to be successful, we start with headmen, if these people are on board, then we have support. There was a time one headman in the community at the meeting gave that testimony that he had this condition for a long time and he did not know about services we offer at the facility until the time we had sensitizations. So if we can have those as champions for the programme, it would be great and I am sure we can do quite a lot.
I: How should local political leaders be involved in the fight for hydrocele?
R: Yes, I think when we have health meetings, it is important to involve the counsellors to build a structure for having outreach activities which can help in sourcing for fund. So I think it should be at all levels, political, church leaders and from the school.
I: Finally, what recommendations do you have on how we can integrate the hydrocele services into community health systems at community level?
R: I think it is a disease which has been forgotten, so it is important to educate many CBVs, such that if they have an outreach service, they sensitize on that regularly.
I: Any recommendations on how to integrate hydrocele services into community health system at facility level?
R: At facility level, I think the way there is antenatal clinic and family planning, I think it is important to have health Corner or a day when we have meetings on conditions affecting men. If they are feeling uncomfortable to be seeing by a female nurse, we can put a man who can be able to examine people effectively and men can get assistance. For men’s health like hydrocele, there is nothing like that.
I: Recommendations at provincial level?
R: For provincial level, I think most time these issues are forgotten became a simple thing like a monthly report, fro reporting and identifying cases is important, just like they do it for Malaria. Focal point persons should be motivated and staff oriented too.
I: Any recommendation at National level?
R: That one I am not too sure.
