# Supplementary material for: Applying community health systems lenses to identify determinants of access to surgery among mobile & migrant populations with hydrocele in Zambia: A mixed methods assessment
Source: PLOS Glob Public Health. 2023 Jul 18;3(7):e0002145. doi: 10.1371/journal.pgph.0002145 (PMC10353788; doi:10.1371/journal.pgph.0002145)
Supplement: S3 File — Data collected and reported in the manuscript. (ZIP) [file pgph.0002145.s003.zip › S2. Datasets/COVID.docx]

Files\\COMMUNITY HEALTH WORKER 1 - § 1 reference coded [ 9.47% Coverage]

Reference 1 - 9.47% Coverage

I= have you heard that there is COVID now.
R= yes
I= the coming of Covid how has it affected your program of hydrocele
R= this Covid is really affected us so much because you wont have times to go and visit other people, or some time start person can not come to you then in the hospital you can not have a lot of people so this has really affected us and distributes us.
I= it has disturbed you
R= yes
I= in which way has it disturbed you?
R= people are not coming in number to the hospital or us we can no have meetings to tell people that now things are like this and that.
I= okay, but these surgical programs have confined.
R= yes if we find that person and he is willing to go yes they go for operation..
I= they go.
R= yes because its once or one person its once or one person and not in a group, they do go. And another problem or issue is that side where they test you for COVID, so people are scared to be tested through their noses, so they just say its better I just say at home, so tat one also has missed up things
I=okay, how has it disturbed those who come from other countries or fishermen. R= it has disturbed us because that one coming from a cross we do not know how that person is because movements have been restricted may be he is the one who has Covid he can bring it out us that is why they have bring it to us that’s why they have said no one should enter but you know how people are, some are entering through other places.
I = okay, so you do not go to the same places and look for those same people in those places who have the same disease.
R= at this time, no we have stopped
I= you have stopped.
R= yes, but we just talk to those meet and see in the community that shows some signs if the is willing he will go but others they do refuse because of what I have said.

Files\\Head Clinical Care LDH - § 1 reference coded [ 2.26% Coverage]

Reference 1 - 2.26% Coverage

I: Covid 19 has been there and it is still there. Has it affected the extent to which you are implementing hydrocele services within the district?
R: No.
I: Meaning all the activities have been running smoothly regardless of Covid 19?
R: Yes.
I: You have been doing surgeries?
R: Yes.
I: You have been able to coordinate and collaborate with other?
R: That one no, no meetings have been taking place.
I: Was it due to Covid?
R: Yes.
I: What about surgeries?
R: Surgeries, we have been performing them.

Files\\IDI - CHW - Mangelengele - § 1 reference coded [ 4.39% Coverage]

Reference 1 - 4.39% Coverage

I: Has Covid-19 affected the way you implement the hydrocele services within this facility?
R: Yes, when it started, there was no moving, no meeting and things were on stand still.
I: Did you suspend any activities like referrals to the hospital?
R: Yes, since that time, when the programme finished, there was no financial support for people to go to the hospital, so the programmes came to a standstill. Even surgeries were not taking place at the hospital.
I: Are there any measures put in place to improve service delivery with regards to hydrocele services? Since Covid-19 came. Like putting things in order to meet the needs of people?
R: Looking at five golden rules and following them as we go in the community. Even the people we meet, we are supposed to teach them about Covid-19 when we finish with the programme we have gone there for.

Files\\IDI - Com Leader - Chitope - § 1 reference coded [ 4.92% Coverage]

Reference 1 - 4.92% Coverage

I: In this covid19 era, you know it is there right?
R: Yes it is there.
I: Do you think covid19 has brought any challenges that has affected how patients access hydrocele services?
R: From the start, Covid19 has disturbed a lot programs like the hydrocele program we started in 2019 and 2020 with Professor Michelo it got disturbed because Professor Michelo and the team stopped coming due to covid19 and then hydrocele surgeries at the hospitals also stopped.
I: Any other challenges that you think came after Covid19.
R: At the clinic gatherings were affected and where we would have 3 gathering per week were reduced due to Covid19 restrictions. In the community you find that as the headman with health workers would want to facilitate for people to get understand hydrocele but the number of gatherings were reduced due to fear of Covid 19 pandemic.
I: Were services targeted at fishermen and migrants, do you think such programs were also affected in their delivery?
R: Yes, there was a challenge instead fishermen coming to access the services and even go to the hospitals to undergo surgery they would rather remain in their field and would not come for fear of moving in numbers.

Files\\IDI - Com Leader - M - Kasinsa - § 1 reference coded [ 14.08% Coverage]

Reference 1 - 14.08% Coverage

I: Due to Covid 19, are there challenges people faced when accessing hydrocele services?
R: Yes. Challenges were there.
I: What challenges were those?
R: There was no longer large gatherings which we used to have to discuss or share information with the people on hydrocele. Home visitation was discouraged as well people were advised to not move around. Physical contact was also discouraged. These challenges made it difficult to make progress in promoting hydrocele services both at community and facility level.
I: Did the same challenges you have highlighted also affect fishermen and migrants accessing the services?
R: Yes! They were not managing to come to the facility to access any service due to Covid 19 movement restriction.
I: Meaning they could not even go to the clinic?
R: Most could not because of Covid 19 restrictions they could not get the necessary information on the available hydrocele services.
I: So, are there ways the clinic came up with to help address challenges caused by Covid 19 in stopping people from accessing services?
R: Yes. Some of the ways which helped were preventive measure like clinic encouraging people to wear facemasks, washing hands and social distancing of one meter apart helped people start meeting again in smaller group when initially people were advised to just stay home.
I: When you look at the help the clinic is giving to help reduce Covid 19 cases so that people can be going to access hydrocele services, do fishermen and migrants have information that they can also access hydrocele services from the clinic during this Covid 19 era provided they follow the preventive measures.
R: Yes. Fishermen also do get the information on how to access hydrocele services amidst the Covid 19.
I: How do they get the information?
R: Fishermen usually come back to the village on Sundays and as the headman of the village I would and announce and talk to them about Covid 19 as well teach them about hydrocele. I would share with them the dangers of Covid 19 and inform that should not stop them from going to the clinic to access hydrocele services. So, once in while they do come to the village and this information is shared with them as well.
I: Covid 19 caused a number of challenges which affected how patients access hydrocele services but going forward, what can be done to make sure that people continue accessing hydrocele services despite the challenges of Covid 19?
R: The most important thing is to continue following Covid 19 prevention guideline such as wearing facemasks. With vaccine here, encouraging everyone to get vaccinated to prevent covid 19.
I: How can we improve hydrocele service delivery in your community?
R: If there can be a vaccine for hydrocele like Covid 19 vaccine injections which can be a prevention instead of waiting for someone to have hydrocele then start getting them to treated for hydrocele.

Files\\IDI - Patient - Kanemela - § 1 reference coded [ 2.92% Coverage]

Reference 1 - 2.92% Coverage

I: Looking at Covid – 19, has it affected you on how you to access hydrocele services?
R: Yeah, it has affected me in some way. You find that sometimes you are not feeling and when you go to the clinic they may not attend to you on time depending with the people you find there. If they are to see you it will be on short time because they want to see others while you are in pain. And with Covid – 19, you are not supposed to be at the clinic for a long period.
I: So that issue of delaying to see you, is it because you are fisherman or it is because of other reason?
R: No, it is not because I am a fisherman but the health staff are also trying to follow the Covid – 19, health guidelines of not keeping patients longer at the facility.

Files\\IDI - Patient - Mpuka 2 - § 1 reference coded [ 4.01% Coverage]

Reference 1 - 4.01% Coverage

I: Covid 19 has been there? Has Covid 19 affected you as a hydrocele patient to access the hydrocele interventions in the past few months?
R: No. have never been found with Covid 19.
I: Not you having Covid 19 but failing or having difficulties in accessing hydrocele interventions because of Covid 19?
R: No, I was still going without any challenges.
I: Alright! Was there any hydrocele service that you failed to access?
R: No. we were able to go to the clinic even amidst Covid 19 by wearing masks.
I: Were you turned away or missed the appointment from the clinic because of the Covid 19?
R: No they just used to welcome us properly.

Files\\IDI - Patient - Sinyawagora - § 1 reference coded [ 4.74% Coverage]

Reference 1 - 4.74% Coverage

I: Has Covid -19 affected the extent to which you as a hydrocele patient are able to access hydrocele interventions over the past months?
R: I can say Covid 19 disturbed many things. But in terms of this condition since it started last year I would say it disturbed me anything.
I: How can we improve the service delivery for hydrocele in our community even in this Covid era?
R: The way we can improve on that is first by creating more awareness programs in all the communities because many people seem not have knowledge on this conditions. And also the number of people to sensitize should be increased because it is like people are many and the facilitators are few. In fact they can create groups in each village to be teaching.

Files\\IDI health provider Chitope - § 1 reference coded [ 5.04% Coverage]

Reference 1 - 5.04% Coverage

I: Covid 19 is there. Do you think so?
R: Yes, I saw patients who presented the symptoms. And I now believe after one patient came and tested positive to Covid 19 then later our colleague was down with the same symptom.
I: Do you think Covid 19 has affected the way you are implementing hydrocele services in your catchment area?
R: Yes, I think being in a rural setup and Luangwa having economic challenges, the moment we started telling people to come with masks, they used to shun the services. Even if we tell people that we have Covid 19 services here, they wouldn’t want to come for fear of being swabbed. This would make the clients with hydrocele shun away from receiving the services due to fear of being swabbed.
I: Describe to me in which way Covid 19 has affected your service delivery in the implementation of hydrocele works.
R: With the coming of Covid-19, I think we have been overwhelmed with work and having very few staff to attend to patients at our facility, it means while doing the testing, one is making diagnosis and because of that we are not able to go out do sensitizations on hydrocele patients.
I: So, there has not been activities that were focusing on hydrocele patients during this Covid period?
R: No, not much unless the patient comes to seek the services from the facility, otherwise following them up has not been done in a while.

Files\\IDI health provider Mandombe - § 3 references coded [ 12.28% Coverage]

Reference 1 - 3.27% Coverage

I: How has Covid 19 affected the extent to which you have been implementing hydrocele services in the community?
R: How it has affected us is that we have situations where we have general screening of everyone who comes to the facility. For instance, you find that people are naturally scared to be swabbed and be tested for Covid. For them, through hearing that when they go to the clinic, they will be swabbed has negatively affected us. Those people might come to the facility and they present themselves with the Covid 19 symptoms. We cannot say since they are hydrocele patients then we deal with their condition, we first focus on the Covid 19 when they have presented some symptoms. However, people are still scared of being swabbed.
I: Do people come to the clinic when they have symptoms of hydrocele even during Covid 19?
R: Yes, they do come.

Reference 2 - 4.71% Coverage

I: How has it affected the implementation process when it comes to surgical procedures? Does it have anything to do with surgical procedures activities, have you been able to send patients and receive the feedback?
R: Yes, provided if the patient agrees, on that one, we have no problem we refer them. The challenge comes in because people have that mentality that they would get Covid 19 from the hospital because there are a lot of people. If it involves surgical, they might stay away from the health facility, for fear of being found positive of Covid and being admitted after been swabbed. Because they know when they go there they won’t say no.
I: Are there times when you have postponed programmes due to Covid 19 with regards to hydrocele?
R: In terms of hydrocele management, no. We never had any programmes for hydrocele that were suspended. It has never happened.
I: What about multi sectoral meetings and collaborations?
R: Yes, in terms of Covid 19, we have done that, especially when Covid was at its pick, we had to stop gatherings where we had chances of sitting together with the community sensitizing them about hydrocele but when Covid was getting out of control, we had no option but to pause the services.

Reference 3 - 4.31% Coverage

I: Has it also had any impact on the service delivery to fishermen and migrants the district?
R: It had because these people stopped moving during that period, even going to the facility, they would feel they were going to be tested for Covid. So this had an impact because they would rather stay home and resort to tradition ways than been tested for Covid.
I: Are there any measures put in place to improve the services delivery to fishermen and migrants, with regards to Covid 19.
R: Yes, measures are there. For instance, vaccinations. When we vaccinate a lot of people, it will help us in the number reduction of Covid 19.
I: Now that there are vaccines, do you think people will still be coming to access services?
R: They will, in the sense that when you vaccinate them, you assure them that they are protected but they still need to mask up. Back then we used to screen everyone. So then if a person is vaccinated and masks up I think it will help.
I: And how can we address the challenges of people not accessing hydrocele services because of Covid?
R: It is a matter of sensitization we educate the community as much as we can.

Files\\IDI_ Health Provider Kasinsa - § 2 references coded [ 9.38% Coverage]

Reference 1 - 7.18% Coverage

I: Okay, Let’s talk about the issue of Covid. So, has Covid affected the extent of implementation of hydrocele services in the long run?
R: Yes, I can say so, number one, there has been little attention that we were giving lymphatic paralysis, it has diverted the funding to Covid 19 response. I can also say there was a time we discouraged people to make unnecessary visits to the centre unless for critical issues. So this made people stay away and there were those who thought when they visit the clinic, they might be vaccinated against their will. This made people stay away from the clinic even if they might have it.
I: Did you suspend any surgical procedures and any activities?
R: At Katondwe, we were advised to refer cases that were urgent and critical, issues like these we were told to wait for appropriate time and now they normalised. But at its pick in June, people were discouraged to go that side unless it is something very urgent. So issues like lymphatic paralysis was not really considered to be serious and we were still told to wait.
I: What about the discussions on how you can improve?
R: And also meetings, gatherings were suspended.
I: What is your comment with regards to the services you were offering directly pointing to migrants and fishermen?
R: Migrants, the borders were closed. So, there was restrictions on travel, so the people who might have wanted to come couldn’t come. Regular outreach services were suspended we could not go out for any program. It was more of social distancing, when you get to service call, there was no spending much time to talk about this health issues.

Reference 2 - 2.20% Coverage

I: Are there times when you have programmes targeting fishermen or to go to fishing camps to talk about hydrocele issues with them?
R: For now we have not done any outreach service to the fishing camps.
I: If at all there is a Community where there are only migrants?
R: The place where I can say thee are migrants is the market place where there are rid mats, there we do some HIV and AIDS services where we distribute condoms. For fishing camps, there are no any specific programs where we go there..
