# Supplementary material for: Applying community health systems lenses to identify determinants of access to surgery among mobile & migrant populations with hydrocele in Zambia: A mixed methods assessment
Source: PLOS Glob Public Health. 2023 Jul 18;3(7):e0002145. doi: 10.1371/journal.pgph.0002145 (PMC10353788; doi:10.1371/journal.pgph.0002145)
Supplement: S3 File — Data collected and reported in the manuscript. (ZIP) [file pgph.0002145.s003.zip › S2. Datasets/Patient experieces with the disease.docx]

Files\\FISHERMAN - § 2 references coded [ 8.18% Coverage]

Reference 1 - 6.10% Coverage

I = how many years have passed since you knew about your disease?
R = It has been six (6) years since I knew about it.
I – so I have stayed with it for six years.
R = the other thing that I can say is that this disease I have some time back I was just okay but now it’s difficult because it takes even about one week without sleeping with my wife.
I = Okay, now tell us talk about your life as at now, where do put your life?
R = My life, the body is just okay the only problem I have is just the disease that I have.

Reference 2 - 2.08% Coverage

I = Okay, do you have other things?
R = Things like what?
I = Like how you relate with your friends going to church.
R = I have no problems with my friends and I do go to church

Files\\IDI - Patient - Kanemela - § 1 reference coded [ 1.56% Coverage]

Reference 1 - 1.56% Coverage

: When you look at your health status, how would describe it? Do you think it is excellent, Good or poor?
R: Health wise my status is bad.
I: Why do you say so?
R: I am say like this because most of the time I fail to finish the works that plan to do as a result of the problem that I have. That is why I am saying I am not good since I have some things I have to do but with this condition, I fail to do.

Files\\PATIENT 1 - § 1 reference coded [ 9.17% Coverage]

Reference 1 - 9.17% Coverage

= I went to the clinic, they tested me and said we cannot find anything in your body, I also went to the traditional healers they said there is nothing in your body.
I= How has hydrocele disease affected your life in terms of work?
Work, English I think I cannot get it.
I mean In terms of work, when you came to know about the same disease, do you manage?
R = No I don’t manage
I = what happens?
R = if I want to work I will only work for a short period of time, I stand for a long time it pains until sit down.
I= okay, what about staying with the family how it being because of the disease?
R= this disease with the family it’s a bit difficult.
I= How difficult is it?
R= If I want to work the other is paining like you are squeezing them.
I = okay so do you manage I want to know everything.
R = you find that one testicle is big and the other one is sick.

Files\\PATIENT 2 - § 2 references coded [ 11.56% Coverage]

Reference 1 - 7.63% Coverage

I = Like how many years ago?
R = about 4 years ago
I = Four (4) years
R = Yes
I = 4 years just
R = from that time I cannot do anything no piece work or anything to do.
I = Okay
R = Even when they are employing, they cannot employ me because am unable to do any work
I = from the time you know that you have this disease of hydrocele, how has been life in this community
R = before I know or I use to do some work like piece work from people but now I cannot
I = what about fishing?
R = to catch fish from the water
I = Yes
R = It’s just the same I have difficulties, it’s really a challenge

Reference 2 - 3.93% Coverage

I = I want to know about the same fishermen who have hydrocele, what makes them not to go to the Hospital to seek for medical attention, why is it that a problem to them?
R = It becomes a challenge for them because if it starts paining they cannot do anything because it pains a lot sometimes

Files\\PATIENT 5 - § 1 reference coded [ 2.97% Coverage]

Reference 1 - 2.97% Coverage

I = So which one was more difficult for you that you could not do?
R = The other thing is that I was not sleeping with my wife very well I used to be very short. So even my wife it was just the love that she has that she even stayed, so when I found some medicine and they went back inside and that’s when I became fine again.
