# Supplementary material for: Applying community health systems lenses to identify determinants of access to surgery among mobile & migrant populations with hydrocele in Zambia: A mixed methods assessment
Source: PLOS Glob Public Health. 2023 Jul 18;3(7):e0002145. doi: 10.1371/journal.pgph.0002145 (PMC10353788; doi:10.1371/journal.pgph.0002145)
Supplement: S3 File — Data collected and reported in the manuscript. (ZIP) [file pgph.0002145.s003.zip › S2. Datasets/Collective action/Intermediary or boundary spanners.docx]

Files\\COMMUNITY HEALTH WORKER 2 - § 1 reference coded [ 3.78% Coverage]

Reference 1 - 3.78% Coverage

I= Okay, so what do you call this place where it we are now and where there is program concerning hydrocele
R= Yes where you said there are no any other groups, so where there’s no such, which place is that?
R= I can trust that all Luangwa
I= The all Luangwa
R= why I have said so there are some people who attended the meeting, chief and others but now when we come on ground level. They are not so active to tell their people to do that, for them to let people know they may have the information but fails to deliver to their people, because in the community you find out the people have no information but the headman has the full information

Files\\COMMUNITY LEADER - § 1 reference coded [ 6.08% Coverage]

Reference 1 - 6.08% Coverage

I = What can you tell me about the help that you received?
R = It was just okay.
I = Just okay how?
R = After I took the drugs I was healed.
I = You only took medicine.
R = Yes only the drugs.
I = And you never went to the hospital?
R = No I did not go.
I = How do you see it, the work that you have done, is it enough work that even those called to can go and tell others about this same disease?
R = yes they even go and their friends because in the past the stories that if you go to the hospital and they operate on you if then you shall not have children, so I tell them no its not true people do have children.

Files\\COMMUNITY LEADER 1 - § 4 references coded [ 16.07% Coverage]

Reference 1 - 6.88% Coverage

I = Do you know the group of people who came from Lusaka?
R = No I just know that they are health people.
I = What about you have you ever taken part in the same program?
R = I did that yes when I was a young boy.
I = Okay, you passed through?
R = Yes, I passed through it year.
I = For how long have you started with hydrocele disease.
R = I was working for judiciary since 1981.
I = Okay
R = Yes, it use to come out and go till last year 2021 that when I though of going for an operation.
I = from that time it has not come back?
R = Now am just okay, I even work hard I have no problem.
I = So, how do you help these people with hydrocele disease, do you take part?
R = Since I am the headman I look for people with that disease then I advice them to go to the hospital so that they can remove it as well. I also inform the nurses if someone had the disease and should go to the hospital.

Reference 2 - 1.78% Coverage

I = Okay, so when you look at yourself as a community, do you think that you do manage when you look at the way programs are moving?
R = Yes I do manage
I = You do manage?
R = Yes because I do encourage people to go for operation.

Reference 3 - 5.25% Coverage

I = Okay, Are there any people or organization that come to ask you about the same disease of hydrocele in this community?
R = Yes they came.
I = Okay what type of things did they ask you about?
R = They asked about the goodness of hydrocele programs and I said its good because people are operated on and good because problems of sickness are no more.
I = Did any one ask you of any suggestions concerning the hydrocele patient how they can improve on it?
R = They asked me that if I go for operation won they cut them and I will stop giving birth? Then I said no when you go to the hospital they will remove the water and not cutting your reproductive organ.

Reference 4 - 2.16% Coverage

I = Okay those who came to ask you questions you never added anything or you did add. do you think those words worked?
R = yes it worked.
I = Why do you say so?
R = I say so because most of them that were they and got those words are able to go to the hospital and be operated on.

Files\\HEALTH PROVIDER - § 1 reference coded [ 2.56% Coverage]

Reference 1 - 2.56% Coverage

I=okay so what about the local people like the people that have been reached with hydrocele and like the all catchment area, are their able to participate actively relating to the implementation like the services that you have been given.
R= yes like those who where worked on last time they are able to implement because the educating others who have the same conditions to be open up to go to the facilities.

Files\\HEALTH WORKER 1 - § 1 reference coded [ 3.01% Coverage]

Reference 1 - 3.01% Coverage

I= so may be let’s look at the babble community here, I want who is involved in the hydrocele disease case.
R= we have our community health workers, of course we the families of the patients we also have the headmen.
I= okay
R= yes, because you cannot do any program, or attar where it is coming from without our traditional leaders, because for you to b able to enter that community you first need to go through them.
I= okay, so can you tell me specific roles that they play for example the community leader, that I may be concerned with this disease, what do they do in helping.
R= they are the ones that Convey the meetings on our behalf
I= okay the meetings
R=they Convey the meetings so that we are able to spread the information is it the mass drug administration, they know what when people know that it has the blessings of the traditional leader s then it is safe.

Files\\HEALTH WORKER 2 - § 1 reference coded [ 7.03% Coverage]

Reference 1 - 7.03% Coverage

I= okay so what new was been put in palace that in making sure that even that said that are holding me are not about able access, what measure have put especially, every in this COVID 19?
R= continues intensive health education
I= what is that?
R= they try by all means when to give out health education in public gathering on how you access our services and then we also those people that goes to give out heath education they make sure that if the local people knows that when you are giving health education the local people able to understand that there are one people who cannot understand what you are saying, so if they identify such a one they will try by all means to try and explain to that person in the villages that he can understand. I think continues intensive health education is really happening.
I= okay I want to hear you recommendation just the way you think we can improve especially those who are doing fishing where do you feel it should be improved the servicers for hydrocele?
R= I think the first thing is creating awareness, educating them telling them what hydrocele is, because as I said some of them just consider it has a natural thing because of may be a case of something and there those people that they and there are those people do not if can be treated so we have to create that awareness by educating them and encourage them that if they have something like that they came to health facilities.

Files\\Head Clinical Care LDH - § 4 references coded [ 11.89% Coverage]

Reference 1 - 1.97% Coverage

I: What about community leaders helping?
R: Yes they are because in their first study, people were sensitized. Most Community Health Workers traditional chiefs they know, so the way they help us is that when they identify a patient, they always make sure that the person comes to the hospital, I will give an example of I think two patients whom I did the surgery on two weeks ago were sent by Chief Mpuka, so there, there is no problem.

Reference 2 - 1.91% Coverage

I: Are there any measures put in place in terms of improving service delivery to fishermen and migrants?
R: There is nothing.
I: On your own, how do you think we can address challenges in future?
R: More sensitizations, to the fishermen chairman first to give us permission to talk to fishermen. Through examinations, you can tell that the person has hydrocele, because a lot of fishermen may have it but they do not know.

Reference 3 - 5.76% Coverage

I: Okay, let us talk about the recommendations on how we can improve the hydrocele services in the district. So will talk about community, local political structures and facility levels. How can the challenges be addressed to improve the issue of hydrocele especially for fishermen and migrants?
R: With the community, there is an area where the fishermen always start from before they go and fish and they have an association, so if health education can be given to them on what is, what causes and what are the benefits of hydrocele can be given to them, it can help a lot. Because once we involve people, they will not be surprised if there are some success stories, we can also take to them for them to learn. And it is very easy for anyone to disseminate information to them at the harbour since that is where most of the fishermen are found. You talk to them, they understand the issues and complication and a lot will come, that is at community level. Then at health centre level, people have just been seeing hydrocele as a disease form the books. So the district office can do a clinical visit on these centres to sensitize on hydrocele, this can help a lot during their monthly visits at these centres also put hydrocele as one of the health problems in the district.

Reference 4 - 2.25% Coverage

I: Any recommendations at local political structures?
R: Those have to be involved because if there is political will, then that is when things can run. If it is the Councillor, town Chairperson is well informed because these are the people who the people on the ground and if the information may come from civic leaders, it may be easy for people follow what they always say. So these are the key people to tackle so that when they come they will talk to the district and the district will take it up.

Files\\IDI - CBV - Kasinsa - § 1 reference coded [ 8.62% Coverage]

Reference 1 - 8.62% Coverage

I: What are the roles of these actors? For example, what is your roles as CHW to ensure there is an implementation of hydrocele services for people?
R: I have a list that I identified for hydrocele people. I go and talk to the patients nicely to tell them to go and drain fluid or go for an operation before they reach 50 years. I even tell them not to listen to the myths that after operation, no more child bearing, I tell them those are lies and that they do not touch the area that produces children. So after explaining to them, they do understand
I: What is the role of the clinical officer?
R: When the client comes, they also do sensitizations and after wards, if the client just wants the fluid to be drained, they do drain them. If they want to be operated on, they are given a referral letter for an operation and they go to the hospital.
I: How about the Headmen, what is their role?
R: In the meetings, they sensitize about this disease and say that there are such diseases in the district and they urge the communities about the kind of diseases that are in the community caused by mosquitoes. There is a mosquito that give malaria and the one that gives hydrocele, so they are told about that.
I: Do you think these actors like you as CHW, the headmen and others have influence on how the services are been delivered in your community?
R: Yes.
I: Why do you say so?
R: I have said so because if I have spoken to someone, depending on how I am and what I have done in the past, they do understand me.
I: Even for the headman if is the same?
R: Yes. They listen to what they say.

Files\\IDI - CHW - Mangelengele - § 2 references coded [ 3.94% Coverage]

Reference 1 - 2.29% Coverage

I: Do you think all these actors involved in the implementation of these services have influence on how hydrocele services are being delivered?
R: They have a great influence because even if they are not paying attention but when they see that both Traditional and Church leaders are talking about the same thing in the community and at church, then it means that the things they are talking about are important and people become serious.

Reference 2 - 1.65% Coverage

I: So you said you collected data for hydrocele? What role did you have?
R: I was collecting data.
I: Okay, is there an effective monitoring and feedback mechanism to help collect the relevant information?
R: Yes, we had Mr Musonda and Mr Chileshe who were consistently making sure that we are doing the collect thing.

Files\\IDI - Chairman - M - Mandombe - § 1 reference coded [ 6.38% Coverage]

Reference 1 - 6.38% Coverage

I: So, do you or other members of society get to influence how hydrocele services are implemented?
R: Yes very much. As a community leader I need to get involved by sensitizing people about any disease including hydrocele because if you delay to access hydrocele services you may become impotent. So we do sensitize and advise the patient to go to hospital as soon as possible and for those with fear of dying I advise them that they don’t kill at the hospital so just go you get the help you need.
I: So, do you as an individual and other members of the community manage to take part programs concerning hydrocele services?
R: Yes we do by teaching people when we see that there is a hydrocele patient at particular home in the community although now there are fewer cases. In the recent past there was a boy with hydrocele so I advised the mother to take to the hospital now the boy whole is whole grown up and doing fine.
I: So, the help you give the ordinary community members do you also get to extend it to the fishermen and migrants?
R: Yes I do. Like I already said earlier I once took a patient from Mozambique to Katondwe hospital and I gave him money before I left but he runaway. After that I took him to another hospital for the second time he runaway again.

Files\\IDI - Com Leader - Chitope - § 2 references coded [ 3.20% Coverage]

Reference 1 - 1.35% Coverage

I: Thank you. The role you play in intervention services do you think you influence how these services are implemented?
R: Yes. As headmen we are the ones who live with people in the villages so as headmen when we speak many people listen to us and do go for the surgery. They all listen but not everyone does what we tell them to do.

Reference 2 - 1.85% Coverage

I: You talked other stakeholders’ active participation in programs concerning hydrocele. Do fishermen also actively participate in all programs concerning hydrocele?
R: The fishermen that have hydrocele and have undergone surgery do try by all means to inform their colleagues who have not undergone surgery but it’s just that some people delay going for the surgery despite their friends that went for surgery have now fully recovered.

Files\\IDI - Com Leader - M - Kasinsa - § 1 reference coded [ 2.52% Coverage]

Reference 1 - 2.52% Coverage

I: How did you get involved in the hydrocele program?
R: When health workers taught us that when people are getting sick in village especially the youth, there will be no development in the area. So, that is how I decided to get involved to help people by influencing them to go the hospital for their condition.
I: Do you get to influence how hydrocele services are implemented?
R: Yes! As a headmen we have influence than ordinary people because when we speak something a lot of people listen to us and follow what we say.

Files\\IDI - Patient - Kansinsa - § 1 reference coded [ 2.41% Coverage]

Reference 1 - 2.41% Coverage

I: What do you discuss when they visit you?
R: When the two men from the clinic visit me, we talk about this disease hydrocele and they discourage the use of traditional medicine saying it will not yield anything in my life and if you see things are bad it is better to go to the hospital rather than going to the witch doctor. Those stories of dying and becoming impotent after the surgery they are all lies.

Files\\IDI - Patient - Sinyawagora - § 1 reference coded [ 1.85% Coverage]

Reference 1 - 1.85% Coverage

I: So are there fishermen or migrants that you know who have come here to seek hydrocele services from your community or within this area?
R: For now there is no one. Those fishermen I knew were already attended to last year and they are the ones who have encouraged me to even come here.

Files\\IDI health provider Chitope - § 1 reference coded [ 3.00% Coverage]

Reference 1 - 3.00% Coverage

I: Do you think these actors have influence on how the services are being implemented in the district?
R: I think they have an influence because I said that there about 2 to 3 clients who came here, but from nowhere, they just felt free and came and said they had a problem that they didn’t want to disclose, but there was a day that we were going round in the villages with the district staff distributing these drugs and showing pictures of the swelling scrotum, lower limbs, that was when he saw us and realised that for these to come and show us these pictures, there must be something that can be done. And that is how he came but for us because of the limited knowledge and knowledge and skills on how to handle such conditions, we referred him to the hospital and I think a surgery was done and he is doing fine now.

Files\\IDI_ Health Provider Kasinsa - § 2 references coded [ 3.28% Coverage]

Reference 1 - 1.36% Coverage

I: Don’t you involve churches, community leaders, and political leaders in terms of the hydrocele services?
R: For now no, previously there was a campaign where we involved the Chiefs Mpuka and Mbuluma. We also involved the Headmen, there were representatives from the church. But the programme ended

Reference 2 - 1.92% Coverage

I: As CBVs, NHCs and community leaders, do they have an influence on how the hydrocele services are delivered in the community?
R: Yes, in our community mostly if you want to do sensitization you need to go through the headmen, because if they are not on board, they might spread negative messages and people stop coming. At least when they are told what will be doing in the community, because the headmen are the community leaders.
