# Supplementary material for: Applying community health systems lenses to identify determinants of access to surgery among mobile & migrant populations with hydrocele in Zambia: A mixed methods assessment
Source: PLOS Glob Public Health. 2023 Jul 18;3(7):e0002145. doi: 10.1371/journal.pgph.0002145 (PMC10353788; doi:10.1371/journal.pgph.0002145)
Supplement: S3 File — Data collected and reported in the manuscript. (ZIP) [file pgph.0002145.s003.zip › S2. Datasets/Collective action/Asset based community development.docx]

Files\\IDI - CHW - Mangelengele - § 1 reference coded [ 2.63% Coverage]

Reference 1 - 2.63% Coverage

I: So whenever there are programmes for hydrocele, are local communities, patients and the stake holders you mentioned participate in the programme?
R: Others participate and other do not, it is not everyone who can manage to participate, some hesitate to take part.
I: What are the reasons why some other actors do not participate in the programs?
R: On that one I don’t know whether it is because of busy or they have other reasons. But for many people when there is a program they attend and participate.

Files\\IDI - Com Leader - M - Kasinsa - § 1 reference coded [ 3.85% Coverage]

Reference 1 - 3.85% Coverage

I: When you come here at the clinic do they ask for information or your views on how they improve the implementation of hydrocele service in your community?
R: Yes. We do advice the clinic from time-to-time to come teach people through drama and people tend to socialize freely. When drama is involved more people come for the meetings.
I: Do you feel your views or opinions have been used to make improvements on the program?
R: Yes. They do consider my views.
I: Do you have an example of you would share with me?
R: Last year when we organized a local drama teaching event. The following day when we went to the clinic we were told that more people had come to clinic to access hydrocele services. This means the clinic considered our advice on drama and more importantly people went to the clinic.

Files\\IDI_ Health Provider Kasinsa - § 1 reference coded [ 2.46% Coverage]

Reference 1 - 2.46% Coverage

I: In the communities, who help out with the support of hydrocele, not necessarily financial but other ways of support to these people?
R: We have neighbourhood health committees where we do outreach programmes, we disseminate that information using CBVs. When it comes to Voluntary Male Circumcision, we also try to integrate other health issues when we go to mobilise because we usually have men and boys, so we tell them even during the VMC that if they have any other problem, they can tell us afterwards, so that we see how we can offer assistance.
