# Supplementary material for: Applying community health systems lenses to identify determinants of access to surgery among mobile & migrant populations with hydrocele in Zambia: A mixed methods assessment
Source: PLOS Glob Public Health. 2023 Jul 18;3(7):e0002145. doi: 10.1371/journal.pgph.0002145 (PMC10353788; doi:10.1371/journal.pgph.0002145)
Supplement: S3 File — Data collected and reported in the manuscript. (ZIP) [file pgph.0002145.s003.zip › S2. Datasets/Collective action/Community monitoring.docx]

Files\\COMMUNITY HEALTH WORKER 2 - § 1 reference coded [ 0.90% Coverage]

Reference 1 - 0.90% Coverage

I= So do you usually have meetings to review where you went wrong and see where its difficulty and what should be done.
R= no meeting
I= no meeting
R= yes

Files\\COMMUNITY LEADER - § 2 references coded [ 9.27% Coverage]

Reference 1 - 7.76% Coverage

I = Do you have sometime where you sit down with the people from the hospital to address the community and talk about how the program is going?
R = Yes we do in the community.
I = do you give them your ideas of how this program should move?
R = Yes we do, we make programs were we tell them it show how the program is going.
I = Okay, So when do you have these same meetings?
R = After three months.
I = okay, after three months?
R = Yes.
I = Is there any time you have taken your view or idea to them that this is what you are thinking and you do receive a feedback?
R = Yes it happens.
I = Can you give me an example
R = Like the time when someone wanted to go for operation, they said the money is not enough because some people need a a lot of money when they go that side.

Reference 2 - 1.50% Coverage

I = Okay, is there any group or an individual who has come to ask about how they should improve on this program of hydrocele?
R = No one came.
I = No?

Files\\HEALTH WORKER 2 - § 1 reference coded [ 4.30% Coverage]

Reference 1 - 4.30% Coverage

I= okay do they have like an opportunity to review how you implement these programs those same people
R= the NGOs?
I= yes those people you find that include trading, business or those you find in this community, do they have opportunities to see how you implement those programs
R= yes they do.
I= why do you say so?
R= because when you send them to go and identify the same people we hold meetings after meetings that when we have the review of all thing we tell them how if went because they bring their names of the facilities and the same people at the facilities, so they expect a feed back after wards.
I= okay how often do these meetings take place?
R= quarterly
I= Quarterly
R= Yes
I= is there any times that you miss quarterly meetings?
R= Yes,
I= What was the reason?
R= We did not have the finances because when going in the fields, those people need some incentives.

Files\\IDI - CBV - Kasinsa - § 2 references coded [ 9.54% Coverage]

Reference 1 - 4.41% Coverage

I: Are local communities, patients and stakeholders able to actively participate in the programme activities related to the implementation hydrocele services?
R: Yes, if there are activities, they do take part in the programs.
I: I am talking about hydrocele, do they come to listen?
R: Yes, for them to hear what will be said, they do that.
I: Does this also extend to fishermen and migrants?
R: Yes, they come. Even the Bembas who come for business do come and attend.
I: Are there any barriers that hinder patients not to effectively participate especially fishermen and migrants?
R: No, nothing can stop them to come here for such activities.
I: Is there anyone you know who doesn’t come?
R: No, we just live as one members in one community and you cannot tell who has missed and which tribe.

Reference 2 - 5.13% Coverage

I: Are there any opportunities for these stakeholders review the implementation of hydrocele services and provide their input in order to improve the quality of services?
R: Yes.
I: Why do you say so?
R: There was a time we were sensitization the programme for the University Of Zambia but before that we had a meeting with different stakeholder at Boma. After that people registered their names and we went to the Headmen to report of the patients we found.
I: Is the Headman found in meetings?
R: In the meetings, yes he is found.
I: Did these stakeholders provide feedback on the implementation program of hydrocele?
R: We were just sharing information on hydrocele and we discussed how we can implement this program
I: How often do you have these meetings where you involve the church leaders, the Traditional leaders, CBVs and others were they provide feedback?
R: Once in a month, we call them when we have HCC meeting.

Files\\IDI - Chairman - M - Mandombe - § 1 reference coded [ 1.22% Coverage]

Reference 1 - 1.22% Coverage

I: Ok. Thank you. Being the chairperson yourself, have you ever been asked about ways you can help improve the hydrocele services?
R: No. I have not been asked before to do that.
I: You have not been asked before to help in that regard?
R: No.

Files\\IDI - Com Leader - Chitope - § 1 reference coded [ 1.33% Coverage]

Reference 1 - 1.33% Coverage

I: Were there any challenges that would make you not comment at the meeting concerning hydrocele?
R: The challenge which was there is we would keep having new people at the meeting because those that attended the earlier meeting would not come for meeting maybe just two would come. They also not attend the next meeting.

Files\\IDI - Patient - Kanemela - § 1 reference coded [ 1.26% Coverage]

Reference 1 - 1.26% Coverage

I: Has there been a time when you were given an opportunity to review the implementation of hydrocele services and you provided the feedback or input in order to improve the quality of services?
R: No, up to date I have never been given any chance to look at the implementation of the services for hydrocele.

Files\\IDI - Patient - Sinyawagora - § 1 reference coded [ 2.50% Coverage]

Reference 1 - 2.50% Coverage

I: What of having a chance of looking at the way programs will run or running? Like calling you for a meeting to discuss about the way the program will run and ask you for contributions?
R: Sometimes they just call a community meeting for everyone to come but there has never been a day when I was called seriously to discuss something on this condition or rather them coming to talk to me.
