# Supplementary material for: Applying community health systems lenses to identify determinants of access to surgery among mobile & migrant populations with hydrocele in Zambia: A mixed methods assessment
Source: PLOS Glob Public Health. 2023 Jul 18;3(7):e0002145. doi: 10.1371/journal.pgph.0002145 (PMC10353788; doi:10.1371/journal.pgph.0002145)
Supplement: S3 File — Data collected and reported in the manuscript. (ZIP) [file pgph.0002145.s003.zip › S2. Datasets/Programmatic lens/Intersectionality.docx]

Files\\COMMUNITY LEADER - § 1 reference coded [ 3.37% Coverage]

Reference 1 - 3.37% Coverage

I = Is there any time you have taken your view or idea to them that this is what you are thinking and you do receive a feedback?
R = Yes it happens.
I = Can you give me an example
R = Like the time when someone wanted to go for operation, they said the money is not enough because some people need a a lot of money when they go that side.

Files\\HEALTH WORKER 1 - § 1 reference coded [ 3.28% Coverage]

Reference 1 - 3.28% Coverage

I= okay are there certain types like patients hydrocele patient that access these services more easily than others
R= yes
I= what are those types?
R= I think the younger ones.
I= the younger ones
R= yes I think the younger ones because they look at the productivity of their lives, if you look at who at the old ones I think they have been living with this program for a very long time and then attributed to traditional medicine, you are like because you did this you are like because of witchcraft, you like this because you slept with some body’s wife now some wants to get back to you, so I think before the public had that much information everything would be kept under laps, people wouldn’t want other people to know that this is the condition that they have, the younger ones because they are looking at their at their you know they family men, they need to provide for their families, they need the comfort and also the marital problems.

Files\\HEALTH WORKER 2 - § 1 reference coded [ 2.50% Coverage]

Reference 1 - 2.50% Coverage

I= so what effect has if brought like to migrates, what effect has Covid brought to them like accessing the hydrocele services.
R= so many effects, these people because of the interview of Covid 19 gaudiness, these people fail to cess the health services, so some of them may be where are coming from Mozambique most of them they follow the guidelines like so when they came here, they find it difficult to access to same heath service because know that there is an interstice of guidelines everywhere.

Files\\Head Clinical Care LDH - § 4 references coded [ 8.99% Coverage]

Reference 1 - 4.79% Coverage

I: But do you think this is the reason why hydrocele patients from fishing and migration population do not seek for help?
R: What I have seen is one of the reasons is that there is a myth that maybe when the surgery is done on them, they will stop producing children, and this is very strong myth, whoever comes for surgery asks me whether they will give birth or not. The other thing as I said, fishing is a 24 hour job may be by 17: 00 they go and comeback the following day and they will need to rest and prepare to go back in the afternoon, so the time for them to come to the hospital is limited, only if things are very serious. If you look at our setting, most men do the fishing and their women are fisher ladies they sell fish, the income is quite low and it is very difficult for them to look for money and come to the hospital. So these are some of the challenges especially on the part of migrants because they know that when they come, they will need to pay for something for them to access the services. But otherwise it is just lack of knowledge on the same condition.

Reference 2 - 1.43% Coverage

I: For the migrants do you think one of the reasons why they do not come is because of fear of discrimination?
R: Maybe, it can be one of the reasons that because they are not Zambians, then that issue cannot be attend to.
I: But is it the case?
R: No, for me I think when you ask them it is lack of money and lack of knowledge.

Reference 3 - 1.55% Coverage

I: What of the fishermen or migrants, do they also participate?
R: Those, no, it is very difficult because the time we meet the migrants is very short because when you see them, within a short period, they are gone. For fishermen, themselves it is very difficult because all they believe is to be in water and do fishing to feed their families.

Reference 4 - 1.23% Coverage

I: So, for migrants, is it difficult?
R: Yes.
I: What about the fishermen?
R: It is difficult for both because for the fishermen, they do not just fish from here. Sometimes they go to Mozambique and fish there for a 1 or 2 months, so it is very difficult for them to come here.

Files\\IDI - CBV - Kasinsa - § 1 reference coded [ 3.39% Coverage]

Reference 1 - 3.39% Coverage

I: Could culture be the reason why it is difficult for people to come to the facility to get help for hydrocele services?
R: Yes, there are different cultures and they say that their culture is supposed to be this and that. Fishermen move around a lot even if they have a problem, they feel they are wasting time instead of doing their business.
I: What about the migrants?
R: Others just choose to ignore and concentrate on their business activities.
I: There is nothing like since they are migrants, they can be discriminated or maybe because they don’t have proper documents?
R: No. There is nothing like that.

Files\\IDI - Chairman - M - Mandombe - § 1 reference coded [ 2.71% Coverage]

Reference 1 - 2.71% Coverage

I: Could it be that migrants also fail to go to the hospital because for them to be attended to they need to produce certain documents when visit the hospital?
R: Yes. For instance, a patient coming from Mozambique or Zimbabwe to access medical services here in Zambia they will need to have a border-pass then that is when they would be attended to.
I: Can language barrier also be the reason why migrants and fishermen fail to go to the hospital?
R: No. Language differences cannot be a reason why someone fails to go to the hospital.

Files\\PATIENT 1 - § 1 reference coded [ 4.41% Coverage]

Reference 1 - 4.41% Coverage

I = can you tell how it started
R= The way it started was that I was feeling some stomach pains and then in the morning you will find out that one side of testicles is swollen then I said what is this ? then I went to clinic I was given medicine which I was taking , shill but the pain was there I further went to the traditional healers I was given medicine , it feels like the pain have gone but if starts again
