# Supplementary material for: Applying community health systems lenses to identify determinants of access to surgery among mobile & migrant populations with hydrocele in Zambia: A mixed methods assessment
Source: PLOS Glob Public Health. 2023 Jul 18;3(7):e0002145. doi: 10.1371/journal.pgph.0002145 (PMC10353788; doi:10.1371/journal.pgph.0002145)
Supplement: S3 File — Data collected and reported in the manuscript. (ZIP) [file pgph.0002145.s003.zip › S2. Datasets/Programmatic lens/accessibilty.docx]

Files\\FISHERMAN - § 1 reference coded [ 5.69% Coverage]

Reference 1 - 5.69% Coverage

I = what happened when you went to see them.
R = nothing happened.
I= it just failed?
R = yes.
I = tell me what happened
R = just medicine for men.
I= Tell me what happened like you are telling me a story.
R = what happened was that we went there to the elders with the view that maybe this thing would come back.
I = is there any other place where you went in order to help?
R= No I never went.
I = why did you not go there?
R = didn’t know that can find that help like that there.

Files\\HEALTH WORKER 1 - § 2 references coded [ 4.44% Coverage]

Reference 1 - 3.73% Coverage

I= okay I want to look at the accessibility of everyone around including even those who are doing fishing activities in a case that they have this disease, what are some of the barriers that they face or if they are no barriers that they face or if they are no barriers are they able to access everything with any difficulties, how do they do that?
R= I think luangwa is one of the few places that I have been to in this country where health facilities is donated around the district I think as even when you driver around the main road you will find servel within reachable of each other, so for example, let’s take the boma, here where we are there is this health post, there is Luangwa boma area, there’s Luangwa district hospital which is 3 kilometres away from here, so with hose within our catchment areas no body travels more 5 kilometres to go and access health services, so even those go fishing even those of the labour should be somebody fail sick there people for port health care there, so I think those are the people like they are the front liner s for everything.

Reference 2 - 0.71% Coverage

I= okay specific for service delivery to those fishermen and migrants where there any effect that caused may reduction or an increase or anything.
R= there was reduction because boarder where closed.

Files\\Head Clinical Care LDH - § 2 references coded [ 3.77% Coverage]

Reference 1 - 1.19% Coverage

I: So, which category of people receives services with ease?
R: I think are those who are near the hospital like the civil servant, marketers, some fishermen and not all because there are those that live very far. Those are the only ones who are near the hospital.

Reference 2 - 2.58% Coverage

I: Why do you think could be the reason as to why some hydrocele patients especially fishermen and migrants fail to access these services for their condition?
R: For the fishermen, I would say there are on the river 24/7 and they always want to support their families and have an income at the end of the day, the only time they find to come here is when things are very bad. The other thing I see is lack of sensitization. There are those who have large hydrocele and those with smaller hydrocele, so for the smaller hydrocele, there are no signs and they think it is normal.

Files\\IDI - CHW - Mangelengele - § 2 references coded [ 5.63% Coverage]

Reference 1 - 2.39% Coverage

I: What are some of the reasons why the migrants and fishermen found here find it difficult to access the hydrocele services that are available at the facility?
R: Most people found here are charcoal burners and selling bananas. But I think some of the reasons are that they are scared to go and pay when they go to the hospital and also a situation of being asked a lot of questions. Some migrant are shy because of their tradition and they do not know our culture.

Reference 2 - 3.25% Coverage

I: Where migrants also tackled?
R: I don’t know if they managed to talk to them after the programme was almost coming to an end.
I: Do the existing health facilities cover all the regions of the district, making it easy for fishermen and migrants to access hydrocele services?
R: Coming to the facility they can come, but looking at the problem they have, they are supposed to go to the hospital for surgery, so I can say they are not managing because at the clinic they are given the referral letter to go to the hospital and there is quite distance for them to reach the one in Katondwe and the other one in Boma at Luangwa Hospital.

Files\\IDI - Chairman - M - Mandombe - § 1 reference coded [ 6.06% Coverage]

Reference 1 - 6.06% Coverage

I: What suggestions do have that can help sort out problems facing hydrocele patients especially the migrants and fishermen?
R: I would say that volunteers and community health workers should ensure information reaches the patients wherever the patients are so the as many possible can rush to clinic to access hydrocele services.
I: Alright. Thank you. What should the community health workers do to combine hydrocele programs with other programs of the clinic so that they run at the same time?
R: What they should do is visit hydrocele patients and bring to the clinic so that they work together with clinic staff so that they stop feeling shy. Health workers should be visiting hydrocele patients regularly to make them feel comfortable with them and if they need medication it has to be given the patients without delay.
I: Alright. What should be done at the clinic to help combine hydrocele programs with other clinic programs so that they run together?
R: They should form a hydrocele committee that oversee hydrocele programs so that they are even able to report monthly on hydrocele cases.
I: As we come to the end of this interview, do you any question or comment for me?

Files\\IDI - Com Leader - Chitope - § 8 references coded [ 16.65% Coverage]

Reference 1 - 3.53% Coverage

I: Sure. So, the first thing I would like you to tell me is how do people with hydrocele live in your community?
R: These people with hydrocele are suffering here. So, they are suffering because they are many and the number of people with hydrocele keeps increasing. They suffer a lot when doing domestic work. They also have difficulties walking long distances because they easily get tired because of the same hydrocele disease they have. So, that’s the major challenge I am seeing people with hydrocele have. In addition, people with hydrocele in our community their manhood troubles them very much as they experience waist pains because their scrotum is filled with fluids then after 2 hours or 3 hours the fluids produce a sound, again the patient will start experiencing severe pains in the waist. So, I would say these people suffer very much.

Reference 2 - 2.26% Coverage

I: Why do you think hydrocele cases keep increasing?
R: The reason why hydrocele cases keep increasing is that in the past when we had a program where people were required to go to the hospital to access hydrocele services by undergoing surgery but very few people went to the hospital to access the available hydrocele services. Also the number of cases have gone up because many of these people like to stay along the river farming and fishing. Then others crossover to their fields in Mozambique for some days then come back here to their homes.

Reference 3 - 1.76% Coverage

I: How about those people that travel a lot like travelling from Zimbabwe or Mozambique to Zambia. Why don’t they go to the hospital to receive services for hydrocele? When they know that these services are available.
R: Those that travel a lot in most cases it is deliberate that they don’t go to the hospital because they have the information (education) about which hospital to go to for such services is available to them.

Reference 4 - 2.42% Coverage

I: Looking at all the health facilities from Luangwa Boma to Luangwa Bridge, do you think the facilities cover all the communities which are there or there are communities which are not covered by these health facilities?
R: Some communities are not covered by the facilities.
I: Meaning they are not able to access the services from these facilities?
R: Yes. Some people have to walk many kilometers to reach the health facility. For instance, people from Kalubinga village have to walk 5 kilometers to get to this facility.
I: But they still manage to come here?
R: Yes. They come.

Reference 5 - 1.71% Coverage

I: How about the fishermen and migrant considering there are long distance to the health facility?
R: Right now, if you see a fisherman come to the clinic just that he is very sick because they do not come to the clinic anyhow.
I: What about the migrants?
R: Since, migrant are ever moving they get to access health services wherever they are when they become sick and find a hospital or clinic to access the services.

Reference 6 - 1.42% Coverage

I: So, even if community health worker follow them they would not find them?
R: Yes. Sometime they just find the children.
I: Are there any measures to help improve service delivery to the fishermen and migrants?
R: Right now community health workers follow these fishermen in their camps around the Luangwa River and talk to them from there.

Reference 7 - 1.32% Coverage

I: Is that for all the programs or for hydrocele only?
R: This happens for hydrocele program as well as Covid19 program teaching them on Covid19 prevention measures.
I: So, they follow them in all their camps?
R: Yes. They are followed to all their camps around the Luangwa River. Even those with hydrocele are followed.

Reference 8 - 2.24% Coverage

I: Do you have any suggestions on how we can address challenges specifically for fishermen suffering from hydrocele in camps?
R: What I would say is we should not stop following and teaching these people who are suffering from hydrocele and also teaching them about Covid19. Despite Covid19 pandemic, us the headmen together with the community health workers shouldn’t fail to teach them. Those that get our messages come to clinic and get referral letter to go to the hospital to undergo surgery. We cannot fail we need to just continue.

Files\\IDI - Com Leader - M - Kasinsa - § 1 reference coded [ 2.75% Coverage]

Reference 1 - 2.75% Coverage

I: So, first of all I would like to find out, how is hydrocele situation in your area?
R: In the past hydrocele was more prevalent because people didn’t know how hydrocele developed and also people had fear of going to the hospital thinking when you undergo surgery then you will stop having children but through sensitization by the health worker together working with the headmen people became aware that even after you undergo surgery you can still bear children. So, right now people do get the medical help they need hence hydrocele cases seem to have gone down.

Files\\IDI - Patient - Kanemela - § 2 references coded [ 3.02% Coverage]

Reference 1 - 1.74% Coverage

I: And does that make it easy for you and other patients from the fishing and migrant population to access hydrocele services?
R: Sure! Sure! Sure! Mainly difficulties are not there except the financial problem where you have to pay something that become a bit of a challenge but not that the facility is far from where I stay. At least each of us as for now can walk like a distance of 5 kilometers to reach the health facility.

Reference 2 - 1.29% Coverage

I: Is it the same scenario with migrants?
R: Like for migrants, it depends with the area where they are coming from like when you go to Mozambique across there you find some.
I: What about those who come to Zambia to seek hydrocele health services, do they also find it easy to access these services?
R: Yes it is easy.

Files\\IDI - Patient - Kansinsa - § 2 references coded [ 5.56% Coverage]

Reference 1 - 3.01% Coverage

I: Have you ever been to the hospital to seek health services for your condition?
R: I only came here last year but I have forgotten the month. But when I came, this clinical officer Tony took me to a room where he drained some fluid which looked yellow. After he drained the fluid, they both looked normal and same but after 2 to 3 day, the same testis reverted to the initial state of this condition and grew big again and so the officer told me that this is the health for here but you need to go to the hospital.

Reference 2 - 2.55% Coverage

I: Are there any opportunities for you and any community members to review the implementation of hydrocele services in your community?
R: No. But the opportunities which are there are those were people don’t meet together but they visit house by house following those patients with this condition. And I don’t know how my friends give feedback in their homes when they are visited since we understand things differently.

Files\\IDI - Patient - Mpuka 2 - § 1 reference coded [ 1.92% Coverage]

Reference 1 - 1.92% Coverage

I: When you look at the existing health facilities from Feira to Luangwa Bridge, do they cover all the regions of the district?
R: Yes, I think many areas are covered. Because other people they don’t just want to go to the clinic to access the services even when they know that they are there at the clinic.

Files\\IDI health provider Chitope - § 1 reference coded [ 1.91% Coverage]

Reference 1 - 1.91% Coverage

: You are welcome. To start with, tell me about the hydrocele cases in your catchment area.
R: In this area, we have quite a number of hydrocele patients, from last year 2020, we had 14 cases, then about 4 of them managed to access surgical services, the other 14, surgeries was not conducted. The other just come to relieve the pressure through aspirations. Where we cannot manage, we refer them to the hospital. This year out of 14, 10 we are still in touch with them and we have identified another 5 and it comes to 15.

Files\\IDI health provider Mandombe - § 1 reference coded [ 3.42% Coverage]

Reference 1 - 3.42% Coverage

I: Do they have specific roles?
R: You mean specific roles for the categories I have mentioned?
I: Yes.
R: So sometimes, we go to the radio station to sensitize these people, we have EHTs who go around in the communities with the help from DHO who provide their vehicles and megaphones to use in the communities. We have CBVs who help us map the catchment areas so that we have the information when the distribution of drugs come, we do not miss anyone. Those who are stationed here are involved as well in terms of creating awareness to the patients when they come to the facility.
I: Do you have a radio station here?
R: Yes, Radio Explorer where we have programmes every Sunday, we pick programmes as a district where we go to sensitize people like hydrocele, or Covid. We have our Health Promotional Officers who have come up with different types of conditions that we sensitize people about.
