# Supplementary material for: Applying community health systems lenses to identify determinants of access to surgery among mobile & migrant populations with hydrocele in Zambia: A mixed methods assessment
Source: PLOS Glob Public Health. 2023 Jul 18;3(7):e0002145. doi: 10.1371/journal.pgph.0002145 (PMC10353788; doi:10.1371/journal.pgph.0002145)
Supplement: S3 File — Data collected and reported in the manuscript. (ZIP) [file pgph.0002145.s003.zip › S2. Datasets/Programmatic lens/Acceptabilty.docx]

Files\\COMMUNITY HEALTH WORKER 1 - § 3 references coded [ 5.92% Coverage]

Reference 1 - 2.03% Coverage

I= So which type of people are there? Those who cannot and those who can what types of these people.
R= People who normally fails are those people who are shy to go to the health facility and they say no me it’s a family issue, those it’s very difficult to help them, then those who are willingly to go and they listen very well and understand help and it’s very easy to help them.

Reference 2 - 0.99% Coverage

R=The other barrier is that people have fear that when operated on you will die.
I= Okay
R= Yes most of them that in mind and they are every scared over that if operated on you will die.

Reference 3 - 2.90% Coverage

R= other barriers? Other Barriers someday it is how we live and other say this the way of Kingdom ship if you have those like in the past.
I= what the way to the kingdom ship
R= Yes that you will look like a chief
I= Chef
R= Yes, to look like a headman
I= Headman
R=Yes
I= Okay
R= In the past that is how they use to say that these are big people they have these things.
I= Okay that how they use to take it?
R= Yes that’s how they use to take it so some still have that in mind and some are just scared of being laughed at that they have being operated on

Files\\COMMUNITY HEALTH WORKER 2 - § 1 reference coded [ 4.51% Coverage]

Reference 1 - 4.51% Coverage

I= Okay, tell me the patients in this community who are suffering from hydrocele, how do they find any help
R= Help like going to the hospital
I= Yes going to the hospital? On this issue of hydrocele
R= Okay, these patients they find help through when we go to them as community workers and tell them so that they know why they get sick, that’s how they manage to get help, but on their own they have their own rules that holds them not to go, so they need to be pushed in order for them to go.
I= Can you tell me what are those traditional rules that they follow?
R= Those people they do not believe that they can be healed form the disease of hydrocele, they believe that those patients are bewitched, so most of them who want help they go to the traditional healer.

Files\\COMMUNITY LEADER - § 1 reference coded [ 3.27% Coverage]

Reference 1 - 3.27% Coverage

R = Some of them is that they do not want people to touch their private parts.
I = Okay
R = So for them to come and no this is what I have or am feeling they feel shy.
I = Okay, they feel shy?
R = Yes.
I = Okay, what other problem would you think is there to prevent these people from not coming?
R = Th ey just want to be like that.

Files\\COMMUNITY LEADER 1 - § 2 references coded [ 9.16% Coverage]

Reference 1 - 5.39% Coverage

I = What is the other challenge that you have noticed that these people face.
R = The other challenge that these people do face I think there is no other problem because these people it’s not everyone who comes to find help here but just very few.
I = Can you tell me what kind of people spear head this same hydrocele problem.
R = For this same disease of hydrocele, there is another group that came early on the same problem that they should write all those with the same problem of hydrocele that they should be helped from the hospital and others do come from Lusaka the first thing we nurses here who are doing registration to those who should go for the operation.

Reference 2 - 3.77% Coverage

I = oaky, is the same for fishermen and migrants, do they add a voice to this same programs of hydrocele?
R = Here are just few and no one goes fishing unless at the boma, so here we are few.
I = When you look at the fishermen, migrants or business people, what do you think is the problem that they do not take part in these programs in many cases?
R = Because the migrants do not put effort to the disease or problem that they have what they know is just business that’s all.

Files\\HEALTH PROVIDER - § 2 references coded [ 6.37% Coverage]

Reference 1 - 3.42% Coverage

I= so what kind of barriers the fishing population and migrants group , what kind of barriers that they face to access services here?
R= one of them is stigma
I=stigma?
R=they have stigma on the same conditions because they separate themselves from being opening up to say they have this
I=why don’t they open up?
R=because of the same stigma
I=so why do you think there’s stigma here based on hydrocele patients
R=because they fear to say this, they look at me to say this person is like this is that, so for the to disclose it, it’s really a challenge

Reference 2 - 2.95% Coverage

I=what recommendations would you have like implementations of hydrocele services?
R=the program should not stop but continue like last time we had about 15 clients and only 6 or 7 were worked on so to those who remained it was really a challenge, it reached at a time where they really wanted help but they could not receive it and also the issues of transport, if these people who goes in the field can be helped with transport, it will be easily for them to find these cases.

Files\\HEALTH WORKER 1 - § 3 references coded [ 5.66% Coverage]

Reference 1 - 2.03% Coverage

I= languages
R= but also the types of migrant that w have mostly are illegal.
I= so they have illegal papers documentations limits, I think they limits their movement , they limits places they can go to unless they are really pressed, but some, then some. They want to hinde no I stay in this place and you know the way you speak gives you away,
R= yes
I= so we try to press them a little bite and them try to tell them am not I do not work for customers am not going to deport you am not going to do anything they come out a little bite, but I think the major, major thing is the language barrier.

Reference 2 - 2.50% Coverage

I= the language barriers, so what happened in the case where you know that this person is illegal, like in Zambia even documentation I illegal, what then do you do as health provider yourself.
R= I think, the policy is the ministry of health is you do not turn any anyone.
I= okay
R= you attend to them
I= yes
R= is for myself personally, I have never reported to crossed the border illogically because I think it’s a long process
I= okay
R= but I think there some people that will take it up for themselves to report such but for us I think we have been told, you do not look at colour, race you do not look at the patient is coming from lets attend to them because they have presented with a problem to you . so they attend to them.

Reference 3 - 1.13% Coverage

R= yes of course help us to determine because we still feel the some who are still keeping the problem to themselves
I= okay
R= you know where you were born and raised here and we know. That coming out seeking the service is the personal thing conviction, someone has to convince themselves to say they need to seek the service

Files\\HEALTH WORKER 2 - § 3 references coded [ 15.40% Coverage]

Reference 1 - 3.92% Coverage

R= okay so we have centers for hydrocele here in our catchment area, this is where people are getting to know that its health condition.
I=okay
R= a long to me they considered this like something like a case, so accessing they never use to access health services, because they considered it coming from ancestral spirit or something yes, so this when they are learning about it and getting to know more.
I= so do they go to right now when they discover that they have got hydrocele
R= okay, because of the intensive of education that we are giving them some of them, they are behavioural change some of the have started coming to the clinic reporting to the health services but some of them that have not acquired information, they still going to the witch doctors to seek interfere from there.

Reference 2 - 3.13% Coverage

= okay are there or any specific people like in thing catchment area that can access services easy than other
R= yes, I think so
I= which type of people are those?
R= I thank female not really females those that are let me say are neighbourhood community.
I= why do say so?
R= because those people are customs, they are used coming to the facilities to help out and do everything and they are more free to interact with the health workers, so they are will never such things even in its something which they are ashamed of and just because they are easily interact with the health workers here, they easily reveal it to the health workers.

Reference 3 - 8.35% Coverage

I= what type of resources are not enough?
R= like I hold you because this the same hydrocele we are talking about, this is when people are getting know about it, to say it’s something helped at the health facility, so people do not just come to reveal this that I have hydrocele, it takes identification somebody to go out there and identify them then speak to them encourage them to come to health facility, so have I forgotten the question.
I= you aim asking about the aim asking if the facilities are enough you said there are not
R= only resource
I= only resources, a n trying to find out what kind of resources are not enough.
R= The resources which are not enough the same resources for after identify them, they need resources and when they come tom the centre health facility for example here we have more than 20 people who have the same conditions of hydrocele, so if they come here at the facility and we do not have proper space for them, we do not have Equipment for them so those are the resources I was talking about.
I= okay
R= because if you send them back home without may be close examination, they still go back to the same behaviour I was talking about.
I= so are there referral system in place so that patients receives adequate care?
|R= something when they came here just after the nurses example them of course they writes for them the referral letters to hospitals. The they have to go side but most of them fail to go there because of transport. Going to the hospitals because most of them are not well to do so, they find it difficult to go to the hospital even at the hospital when they all them may be go and buy drugs, expensive drugs so that they fail to do that because of money.

Files\\Head Clinical Care LDH - § 1 reference coded [ 3.11% Coverage]

Reference 1 - 3.11% Coverage

I: Why do you think could be the reason as to why some hydrocele patients especially fishermen and migrants fail to access these services for their condition?
R: For the fishermen, I would say there are on the river 24/7 and they always want to support their families and have an income at the end of the day, the only time they find to come here is when things are very bad. The other thing I see is lack of sensitization. There are those who have large hydrocele and those with smaller hydrocele, so for the smaller hydrocele, there are no signs and they think it is normal. There are even traditional beliefs that cause them not to come to the hospital, so we had cases where a patient comes,

Files\\IDI - CBV - Kasinsa - § 2 references coded [ 3.76% Coverage]

Reference 1 - 1.56% Coverage

I: What are some of the reasons why the migrants and fishermen find it difficult to access the available services for hydrocele at the facility?
R: Others it is lack of information, others are discouraged and others it is fear of been discussed by others or stigma in the community.

Reference 2 - 2.20% Coverage

I: Do they charge hydrocele patients when accessing the services?
R: They do charge them as long as they have been referred.
I: For hydrocele only or any other disease?
R: There was one person I took for the same services to the hospital, but there was an organization from the University of Zambia which paid for all the services. Since they came, at least they tried bring change in the community.

Files\\IDI - CHW - Mangelengele - § 3 references coded [ 10.02% Coverage]

Reference 1 - 3.88% Coverage

I: So today the 6th of October 2021 I am at Mangelengele Health Post, talking to one of the Community Health Workers. So, I explained about the whole programme, to start with, tell me about hydrocele situation here in Mangelengele?
R: The hydrocele situation here at first it was very high, we had a lot of people that had this condition but most of them where not coming to the facility to seek medical services for their problem, but after that study that was conducted in 2019 by the NGO, we started educating them, sensitizing them, then people started coming up. So we had recorded some numbers and out of those numbers, there were some people who managed to go for surgery and some were left because the numbers that were needed had been reached.

Reference 2 - 2.07% Coverage

I: Like for those people who have hydrocele in this area, where do they first go to seek for help?
R: A lot of them go to traditional healers and get traditional herbs. Because from the information that I ask them, they say that this problem can be healed by the traditional medicine and they think that they might be bewitched by someone so they go to the traditional healers for help instead of clinic.

Reference 3 - 4.06% Coverage

I: Others?
R: Others think that it is genetic and that it is inevitable to have it until death, the knowledge of going to the clinic is not there.
I: Do most people who discover that they have hydrocele go to traditional healers or the health facilities mostly?
R: A lot used to go to traditional healers and we never knew as a facility that such conditions existed because they never used to come to register. After that, they started coming and they know that the disease can only be treated at the clinic. So those who manage to open up come and we explain to them.
I: Who do they come to see when they come here?
R: When they come they see the nurse in charge. But there are also others who find difficulties to come and say their problem and they have continued going to traditional healers.

Files\\IDI - Chairman - M - Mandombe - § 4 references coded [ 17.79% Coverage]

Reference 1 - 4.93% Coverage

I: What services do people suffering from hydrocele receive when they go to the hospital?
R: When patients go to the hospital they access hydrocele services such as medication. Note that in some patients hydrocele makes them experience severe waist pains they need medicine to relief the pain. So, when the patients go to the hospital to register to access hydrocele services they will be treated and the problem will eventually go away after the treatment. Then, there are those patients who hide their condition for fear of being stigmatized such patients tend to suffer for a longtime. That is why when hydrocele patients get to be told or talked to by health workers or doctors that they shouldn’t feel shy because of the hydrocele condition they have and that they should just go to the hospital to seek help and get better. Talking to the patients will result in them going to the hospital to seek hydrocele services and eventually the patients get better.

Reference 2 - 3.95% Coverage

I: Do you think if a hydrocele patient from Mozambique and a Zambian hydrocele patient both went to the hospital to access hydrocele services would they be attended to in the same manner or it would be different?
R: Patients get to be treated the same just that some patients have fear that once they undergo hydrocele surgery they will die. An example is, I once took a hydrocele patient from Mozambique to the hospital so that he can access hydrocele services he run away from the hospital because he was afraid of dying after surgery. The second time he was taken to the district hospital he runaway again before accessing the services because he was still afraid of dying as result of surgery. Otherwise, all patients are attended to in the same manner no discrimination.

Reference 3 - 1.91% Coverage

I: Apart from dying, what makes fishermen and migrants fail to go to the hospital to access hydrocele services?
R: Many patients that fail to go to the hospital are those that do not know the benefits of going to the hospital while others it is because they fear being stigmatized. These are some of reasons why patients fail to go to the hospital to access hydrocele services.

Reference 4 - 7.00% Coverage

I: I would like to hear your views on what we can do to improve hydrocele services?
R: If NGOs and volunteers get involved in teaching people in the communities about hydrocele that would help improve the services otherwise if there are no volunteers to get involved these patients will just be staying in their homes with treatment. Hence, volunteers and NGOs should be teaching people about hydrocele to help even reduce the shyness and shame associated with hydrocele. You would find that at times individual can have hydrocele but because they feel shy they end up not going to the clinic. The sad part the condition worsens with time. In addition, in the past people used to refuse to go to the clinic to access available hydrocele services some patients due to fear. This is why volunteers should be teaching people that if you have hydrocele and you would like to get cured you should visit the clinic. If it is surgery you should go to the hospital so that the problem is completely cleared. Interestingly, a lot of young men would refuse to go to the hospital to undergo surgery due to fear of becoming impotent after the surgery but at the hospital they should be telling them that they don’t cut the reproductive veins but the one that fill up the scrotum with fluids. So, I would say they should continue teaching people about the benefits of going to the hospital.

Files\\IDI - Com Leader - Chitope - § 2 references coded [ 7.72% Coverage]

Reference 1 - 3.79% Coverage

I: What help do patients get when they go to the hospital and come back?
R: I talked about things that are needed so that patients don’t travel long distances to Katondwe hospital or somewhere else. Like I said there is need to build a separate surgical room because someone with hydrocele usually feels shy to enter the clinic where there are a lot of women around. So, there is need to build a separate surgical room on side where hydrocele patients can undergo surgery separately from others. Not a situation where hydrocele patients are frequently mingling with a lot of women because what usually happens is a when a hydrocele patient keeps making frequent eye contact with women they will feel shy and uncomfortable so you find they just decide to step out of the clinic for a moment but instead they go for good without been receiving available hydrocele services they came for. That is the last thing I can say.

Reference 2 - 3.93% Coverage

I: I have said that there people that move a lot staying in different places for various reasons some of which is business while others do fishing. So, let us focus on fishermen. Why don’t they go to the hospital to get help for the hydrocele condition they have?
R: Some patients it is shyness. They feel shy to go to the hospital. For other patients their women in homes tell the patients that if they go for that surgery there will be a problem in bedroom performance thereby affecting their romance. Some patients have heard people say that if you go for the surgery you will die just a days after the surgery. All these makes it difficult for some patients to go to the hospital to get the help they need. An example I can give is, the two gentlemen we came with, we have been talking to them for a longtime about accessing hydrocele services but they have delaying to undergo surgery. So, maybe now they will accept to access available hydrocele services.

Files\\IDI - Com Leader - M - Kasinsa - § 2 references coded [ 6.76% Coverage]

Reference 1 - 2.92% Coverage

I: What else apart from shyness?
R: Shyness makes most people fail to come to seek help. They feel shy opening up saying the hydrocele problem brings fear of being stigmatized.
I: So, even fishermen feel shy to go the clinic?
R: Yes.
I: What about the migrants?
R: As you know hydrocele is found on the private part of the body so even with them, it becomes difficult to share with other person or people.
I: But for migrants they are just visitors how can they also feel shy?
R: Some fear that once I am diagnosed with hydrocele at the clinic they will not keep it confidential but share that with other people.

Reference 2 - 3.83% Coverage

I: How are you personally involved in supporting the provision of hydrocele services?
R: As headmen, we also work like volunteers. So, when I find someone I suspect has hydrocele I will talk to that person nicely encouraging him to go to the hospital as the available services are free. Furthermore, I will inform the patient that he will not be stigmatized when he goes to the clinic. Some patients fear going to the clinic because they have heard that once you undergo surgery you stop having children. So, you tell him no that is not true you are still going to have children after the surgery. After nicely talking to that person you will find that the following day he goes to the clinic. When patient undergoes surgery and fully recover many come back to say thank headman I am now better.

Files\\IDI - Patient - Kanemela - § 2 references coded [ 6.04% Coverage]

Reference 1 - 3.93% Coverage

I: Since you are a fisherman, what do you think are the reasons why you as a fisherman together with other fishermen who have the same condition were not going to the clinic to access the available hydrocele services? So first talk about your personal reasons and then mention for others
R: My personal reasons are that what used to trouble me to go to the clinic back then was being shy. And this issue of shyness although I have said it on my own but it also applies to others. That is what used to hold us back from going to the clinic for fear of being laughed at. And the other thing that usually happens especially when you are going for an operation personally is that we fear since I was operated on one side, I am afraid of going for an operation on the other side because when I have an operation on the other side I can become impotent. So that is where I am stack unless I have information which can help me in my future. Looking at the situation I am in, it is not good.

Reference 2 - 2.10% Coverage

I: So you mentioned earlier that other fishermen do not seek the services for this condition because of being shy, what are the other reasons?
R: On the other fishermen I cannot say much but it is mainly an issue of being shy. You find that a person has this condition but he is failing to go to the clinic to receive the help because of the part in which the condition is located at least if it was in the hand or leg but in that area which requires you to undress and that is why many of us feel shy to visit the clinic for help.

Files\\IDI - Patient - Kansinsa - § 4 references coded [ 13.17% Coverage]

Reference 1 - 2.69% Coverage

I: Meaning it was in 2002. So how did this problem start?
R: So it started first with some pain and that is how I went to an African doctor and I was given some traditional medicine. Then I was also given some medicine which I was told to drink but the pain has reduced while the scrotum is still growing. Until now there are times when it will stay for some time with any pain, if the pain is there it will be for short time but the scrotum continues to grow big.

Reference 2 - 6.24% Coverage

I: Have you ever been to the hospital to seek health services for your condition?
R: I only came here last year but I have forgotten the month. But when I came, this clinical officer Tony took me to a room where he drained some fluid which looked yellow. After he drained the fluid, they both looked normal and same but after 2 to 3 day, the same testis reverted to the initial state of this condition and grew big again and so the officer told me that this is the health for here but you need to go to the hospital.
I: So have you gone to the hospital?
R: No. Not yet.
I: Have you been helped or guided on how you can go that side at the hospital?
R: No, I was just told that I should come here any day to get the paper that I will need to go with to the hospital
I: From that time you were told, have you been here again?
R: No. I have not come here again.
I: From the time they have been draining the fluids, do you feel there is any difference or it is just the same?
R: There is no difference because when they drain, it is just 2 to 3 days and then it goes back to the same state.

Reference 3 - 1.21% Coverage

I: Are there other people who have hydrocele that you know? And how are they?
R: Yes, so others they feel shy as if they are talking with a lady meanwhile they are talking to a man. So those I know are about 5.

Reference 4 - 3.03% Coverage

I: What makes you and those others being hydrocele patients difficult to access hydrocele services available at the clinic?
R: That is why I said we think differently. One of the reason according to me is that the operations conducted this time is done by the young nurses from college and after the operation is done, you also die. The second reason is that people say once you undergo a surgery, you become impotent. So these two points, are the one which makes me and my friends not to access the services.

Files\\IDI - Patient - Mpuka 2 - § 3 references coded [ 6.47% Coverage]

Reference 1 - 4.20% Coverage

I: Do you have some people who have hydrocele and they are either fishermen or migrants within your community or where you work from?
R: Yes but they are not many. Like for others you can’t tell that they have hydrocele. The person I know had it and it was very big. So they went to the hospital and he underwent a surgery.
I: What do you think are the reason why it is difficult for patients who are fishermen and migrants to access the hydrocele services available with the facility or district?
R: I think many of them it is because they are shy with the people at the clinic, you find that they know each other and they feel shy to go and seek hydrocele services there.

Reference 2 - 0.72% Coverage

I: What else apart from being shy?
R: Others it is the fear of undergoing a surgery where they feel they will be cut.

Reference 3 - 1.55% Coverage

I: And do you anyone with hydrocele who went for surgery.
R: Yes there is my friend who went for surgery at the hospital and he is fine, he was not managing to cycle a bicycle but now he does that and he also able work properly without difficulties.

Files\\IDI - Patient - Sinyawagora - § 2 references coded [ 9.08% Coverage]

Reference 1 - 5.68% Coverage

I: Meaning what now?
R: Meaning that if the pain persist in the veins, they need to work on it referring to this same hydrocele. So I went there late when the people who were doing that were planning to end their program. So all this long I have just been trying other means and people advised me to use traditional medicine which I did and nothing is changing. Until I reached a point were things were worse and I came to the clinic where I was told to wait saying there will be a time when people will come to assist you on your condition. So that is when I met who works at the clinic going round in our village and I asked him about the program of my condition and he told me he was actually looking for those, then I signed the papers. He further told me to look for other men with a similar condition who may be hiding that condition. And I managed to find two men and I referred them to him.

Reference 2 - 3.40% Coverage

I: There are some fishermen and migrants who also have hydrocele, now what are the reasons that make it difficult for you and those other patients from the fishing and migrant population to access hydrocele services?
R: Yes they are there but I have never heard any reason why these people do not go to the hospital to seek hydrocele services. But I think they just need to remove the fear in them because I know it could be fear that makes them not to visit the hospital for their condition as you know us people are different.

Files\\IDI health provider Chitope - § 1 reference coded [ 1.70% Coverage]

Reference 1 - 1.70% Coverage

I: The explanation I got on some reasons why people do not come to access services was people are shy, others because they think there is no help that they can get, others you are saying, they do not know, so there is need for sensitization.
R: Yes others its tradition and others are scared of surgery because they think when testicles are to be removed they will not be able to have children and others do not want others to know that they have such conditions.

Files\\IDI health provider Mandombe - § 3 references coded [ 5.22% Coverage]

Reference 1 - 2.46% Coverage

I: We are looking at hydrocele and our main focus will be on fishermen and migrants. How do hydrocele patients and other community members living within this community, go about accessing health services for their condition?
R: On that one, the way they go about it, usually they come here then after screening them, sometimes they might not even know it is hydrocele, basing on their explanation, when you examine and you tell them to say this is hydrocele. If the extent of the condition cannot be managed here, we refer them to the hospital, so they have access to the health facility, then after better findings we refer to the hospital.

Reference 2 - 1.43% Coverage

I: What could be some of the other reasons that make it difficult for them to access the services?
R: Maybe, even some cultural beliefs, most times the things I have heard are that when you have got swollen scrotum, they say it is because they slept with someone’s wife and for this thing to go they need to go to traditional healers. So cultural beliefs is one of the reasons.

Reference 3 - 1.33% Coverage

I: What of those from the fishing population?
R: For the fishing population, they are locally here, they access because we make sure we tell them the way it is and we say at the end of the day if they are not cured of hydrocele, they will stop going for fishing. So those locally, despite their activities they do access and accept when we educate them.

Files\\IDI_ Health Provider Kasinsa - § 2 references coded [ 4.95% Coverage]

Reference 1 - 2.51% Coverage

I: So, having such people on the board, what could be the other reason why they do not come to seek for medical attention? Because others obviously live here in Zambia.
R: Looking at our setting, this is a rural area, so the levels of education, they might have reached up to grade 5 or 3 and they like getting information from the traditional healers and sometimes the way the information is presented, some do not understand while others have misconceptions and other beliefs concerning hydrocele, so those things can affect how the person may access services.

Reference 2 - 2.44% Coverage

I: What about on theissue of social cultural beliefs?
R: Like I said, some think they were bewitched and some do not prefer been seeing by a female person. Like the person I saw yesterday, he has been coming for 4 times and he has been finding a female nurse, until I came back yesterday, I checked on him and found that he had a very large hydrocele, so there are those issues where elderly men don’t want to expose themselves to females and some end up saying that the nurses can be their daughters. So some of these issues can be a hindrance to some people.

Files\\PATIENT 1 - § 2 references coded [ 13.22% Coverage]

Reference 1 - 4.41% Coverage

I = can you tell how it started
R= The way it started was that I was feeling some stomach pains and then in the morning you will find out that one side of testicles is swollen then I said what is this ? then I went to clinic I was given medicine which I was taking , shill but the pain was there I further went to the traditional healers I was given medicine , it feels like the pain have gone but if starts again

Reference 2 - 8.82% Coverage

I = here in your village or community do you have some people who find it so easy to find help compared to others concerning this same disease?
R = yes
I = what types of people are those
R = Like the man who is outside
I = which main?
R = my grandfather.
I= why do you think that it’s so easy to find help through him than others.
R= because he has the same problem just like mine (Hydrocele disease)
I = like which problem?
R = problem of hydrocele the way it pains, the same he is feeling that’s the way am feeling also.
I = About what about? About those who find help so easy, what types of those people? do they exist?
R = Yes they are there
I = what types of people or what groups are those which find help that others?
R = there is a man in the house who is also a carrier of Hydrocele disease, it pains like mine also.
