# Supplementary material for: Applying community health systems lenses to identify determinants of access to surgery among mobile & migrant populations with hydrocele in Zambia: A mixed methods assessment
Source: PLOS Glob Public Health. 2023 Jul 18;3(7):e0002145. doi: 10.1371/journal.pgph.0002145 (PMC10353788; doi:10.1371/journal.pgph.0002145)
Supplement: S3 File — Data collected and reported in the manuscript. (ZIP) [file pgph.0002145.s003.zip › S2. Datasets/Programmatic lens/Quality of services.docx]

Files\\COMMUNITY HEALTH WORKER 1 - § 1 reference coded [ 4.15% Coverage]

Reference 1 - 4.15% Coverage

I =when you look at the same book does if show you what is required in the community? When you write the names of people in the community they will tell you that is required is this what is that.
R= that book, there are some books that your writ whether that person has hydrocele write his name, put his age the even take the picture of that person the way he is, so if we tell someone ton say this person needs an operation. Then we use to have some people where they are not suffering from hydrocele may be it is what they say what can I say you just find out that his person is not hydrocele is just a problem of hyner, this won’t go for an operation no. so even those people we use to refer then them then the doctor will tell us this one is not hydrocele but this is hyner he has.

Files\\COMMUNITY HEALTH WORKER 2 - § 3 references coded [ 7.65% Coverage]

Reference 1 - 1.16% Coverage

I= what about the Equipments in these facilities do they have?
R= I can lie, if I can yes there are there people who goes there they manage to be worked on meaning everything are okay.
I=okay
R= yes

Reference 2 - 2.43% Coverage

I= okay, what about the community health worker, is the number okay
R= CHW I can say
I= Yes in every area even reaching on the fisheries.
R= No there are not enough
I= Not enough.
R= yes, like under boma where we have this program we are just two of us but when we look at the catchment area where we do go, its big
I= its big
R= very big, so the CHW are not enough under boma where we are us the area where we go is two big.

Reference 3 - 4.06% Coverage

I= did these CHW did their training
R= yes we received training
I= what type or kinds of those training
R=we received different training s we learnt about how to ask our questions whilst in the field and how to help them with their problems, so in short I can say training we did.
I= okay, what is the most difficult thing to the CHW the major one?
R= for CHW Like what I said under our facility the community is too big we face the problem of transport for us to move from one village to another village, so if we can have bicycles then we can manage to do much, the last village from the boma is a kilometre that how it is, so we have to move from a kilometres and for us to finish it’s a problem.

Files\\COMMUNITY LEADER - § 1 reference coded [ 4.20% Coverage]

Reference 1 - 4.20% Coverage

I = about those who look after these people with hydrocele, are they enough?
R = Yes, they manage.
I = they manage?
R = because the number is not so much.
I = When you look at the number do you think it is the correct number?
R = In our catchment we need some people to help us, in terms of fighting against this disease of hydrocele.
I = what about the training that people go through to teach these people
R = Yes

Files\\COMMUNITY LEADER 1 - § 2 references coded [ 6.46% Coverage]

Reference 1 - 0.99% Coverage

R = Yes
I = Why do you say so?
R = I say that because they always refer to Katondo hospital where they have all these equipment

Reference 2 - 5.47% Coverage

I = Okay, what about the guidance or guideline that help the patients to be treated is it just okay for you?
R = Yes it’s okay
I = Why do you say it’s just okay?
R = I said it’s just okay because long time ago people were not aware and did not want to go and remove it, but today after they have learnt they are free to go for an operation.
I = Okay can you tell about the people who looks after the patients of hydrocele, are they enough to manage all the patients?
R = It looks like they do manage especially at our hospital.
I = They do manage?
I = Yes when you look at the training that were offered are good enough?
R = The training was just okay because that which they wanted is happening.

Files\\FISHERMAN - § 1 reference coded [ 2.49% Coverage]

Reference 1 - 2.49% Coverage

I = what about the equipments to use do they have in the clinic?
R = there is nothing
I = why don’t they have them?
R = here they have not done anything like that
I = here, what do you mean?
R = here at our clinic

Files\\HEALTH PROVIDER - § 1 reference coded [ 4.45% Coverage]

Reference 1 - 4.45% Coverage

I= those healthcare and community based volunteers the ones who are able to provide services to hydrocele patients, are there enough?
R =No
I=they are not why do you say saw?
R=to our catchment there are not enough because villages in our catchment area are very far apart, hence more workers are needed.
I= they there received training to do the hydrocele services?
R=like basic ones, yes
I=they have done?
R=yes
I=what do you mean by basic ones? What kind of basic training did they receive?
R=the one to educate and sensitization
I=okay
R=yes
I=so what are the main Human Resources what are main challenges?
R= staffing
I=staffing?
R=yes
I= what do you mean by staffing?
R=we don’t have enough to do the work

Files\\HEALTH WORKER 1 - § 2 references coded [ 4.34% Coverage]

Reference 1 - 0.94% Coverage

I=okay
R= so which I think the hospital can benefit from, because our patients the surgery that are at the district hospital we do the mobilization afterwards we do the home visit to check on how the patients are doing but the actual procedures are done at the hospital.

Reference 2 - 3.40% Coverage

I= okay, so are there adequate like health care providers or community based volunteers that are there full time to flight this disease? Are there enough.
R= if your asked me, we always want more
I= They always want more
R=Yes we always want more
I=You always want more.
R= Yes, we always want more I think the unto enough health care workers in the community for any particular program and this include even this.
I= okay, so you say there nto enough
R= They are not enough
I= So those that are there now were they trained?
R= They were trained.
I= They received a specific training
R= They did.
I= Was it specific hydrocele training? Hydrocele disease training.
R= I think it was, it was the training came about because of the project that was about to take place.
I= Yes
R= So how do you identify the patient if you do not how the symptoms where, so it was specific.
I= Okay
R= even I think there are always orientations, refresher and it time for mass drug administration.
I= Okay

Files\\HEALTH WORKER 2 - § 2 references coded [ 12.09% Coverage]

Reference 1 - 1.34% Coverage

I= Okay
R= Like transportation because villages are further apart. They need transportations like may be bicycles and some forms of recognition may be they wear ID, T-shirts or cops but they did not have such so they needed some refreshment, because it’s a heavy job.

Reference 2 - 10.76% Coverage

I= Okay, health providers including the community health workers associated to hydrocele, are They enough?
R= No
I= Why do you say so?
R= They are not enough because I believe that just in catchments area, they are a lot of people with hydrocele problems so those people that goes around to identify them are not enough?
I= they are enough?
R= yes they are just a few so we need some people to help them may be each village we need three to four people so that they can be going around.
I= those who are there the community health worker what training have they gone through.
R= they received the same training skill concerning hydrocele program.
I= okay, how long was the training?
R= a day just a day.
I= what do you think is their main challenges
R= with that training?
I= no with community health providers what is their main challenges.
R= I think the main challenges is transportation villages are further apart and just some incentives for motivation.
I = okay
R= yes
I= To the local facilitates like here, do they have a guideline to utilize for hydrocele patients.
R=The guidelines the written ones
I= Yes, this facility including for community health workers.
R= we do not have any guidelines.
I= you do not have
R= Yes
I= so you do not follow like a specific for any patient hydrocele?
R= we only follow what were because they went they got oriented so they came and tell us what to do, they were just told verbally, that’s the same verbal guidelines we follow.
I= so are their given any incentive?
R= no
I= no incentives
R= only when they went for the same training now they just do, they are not given anything.
I= so what do you think they are doing just like that?
R= may be there no money
I= there is no money but for, on their side what do you think they just do it without any incentives.
R= which ones?
I= Community health worker
R= why they just do it
I= Yes
R= Because they have acquired knowledge and knowing the important of hydrocele patients accessing the health services so they have just taken it personal and they just go ahead and it despite them not having any incentives but I think if the incentives where there I know those are going to be doing more than they do.

Files\\Head Clinical Care LDH - § 4 references coded [ 13.41% Coverage]

Reference 1 - 2.85% Coverage

I: So at the health centre, what are the main services they receive?
R: At the health centre, there is nothing they receive. The only thing they do is to refer them, otherwise, nothing is done unless when the patient is in pain, that is when they give a pain killer.
I: When they refer them, what is it that they receive?
R: When they refer them to here, we do the initial assessment to confirm whether it is hydrocele or it is something else. So if it is a confirmed hydrocele, we schedule them for operation to be done mostly on Tuesdays and Thursdays, they would stay in the hospital for 2 days, then the third day they are discharged.

Reference 2 - 2.30% Coverage

I: What could be some of the reasons why these people do not pass through the centre? Or why do the centres refer the people here?
R: They do not have the skill to do the hydrocele operation. Most of them are not trained to do the surgery.
I: Could there be any other reasons as to why people are referred to this facility?
R: I think some it is lack of knowledge they don’t know the problem they just find that the scrotum is swollen and they do not know that it is hydrocele so they just decide to refer to the hospital.

Reference 3 - 3.75% Coverage

I: Do they have the adequate equipment that they need if someone with skill was to be sent there?
R: Yes, because what it is, the equipment needed for you to identify is a torch then you see that it is fluid. It would be very easy for them to identify if it is hydrocele just at the facility.
I: What about in terms of surgery?
R: In terms of surgery, the surgery is aseptic so if they have a room which is sterile, it is not very difficult. Some people can be trained and sometimes you can just aspirate the fluid. The other thing is that if you see that the surgery for hydrocele is difficult, you can just remove just remove the fluid to reduce it but you are not treating but just aspirate.
I: But are there adequate rooms that these services can be done from?
R: Yes some centres have the rooms but at other centres no, they do not have them.

Reference 4 - 4.51% Coverage

I: What gaps exist in the available infrastructure for these facilities to provide services for hydrocele?
R: So like these facilities, for the clinic like Luangwa urban clinic, what is needed is a room, enough light and examination coach, and probably, even a few surgical instruments may be needed.
I: Are the community health workers or health care providers provided with any incentives to improve their performance when implementing services for hydrocele?
R: Now?
I: Let us talk about now.
R: There is nothing.
I: When was the last that they were provided with the incentives?
R: When we had the school of Public health implementing this program, for now, no incentives.
I: Before the school of Public health came in and after it left, where there any incentives to motivate them?
R: Nothing was provided.
I: What about during the UNZA period?
R: During the period, incentives were given to the providers and the patients after discharged, they were also given transport refund.

Files\\IDI - CBV - Kasinsa - § 1 reference coded [ 16.40% Coverage]

Reference 1 - 16.40% Coverage

I: Do the health facilities in Luangwa cover all regions of this district making it easy for fishermen and migrants to health services?
R: Yes, they all know where they are supposed to go to for the hydrocele.
I: From Luangwa Boma or Feira to Luangwa Bridge you carter for all those areas?
R: Yes, not through me but my other friends in the other health facilities.
I: Why do you say so?
R: Because I know that they are catered for since each and every area, we came together and we were taught together with the others how to manage cases at the clinic.
I: Do the health facility have enough equipment and supplies to provide these services?
R: Equipment and also CBVs are not enough because they cater a big area.
I: What do you think should be done in the available infrastructure to provide these hydrocele at the facility?
R: What I can say is that we need a lot of CBVs to be recruited and be trained, we need the equipment as at now there are no surgeries taking place but only draining of fluid is done. They should also train someone to be able to do surgeries on hydrocele patients.
I: As CBVs, do you use any guidelines to implement hydrocele services?
R: I have guidelines that I follow. They have messages of hydrocele and some pictures that show the severity of hydrocele. That is what we use to teach them.
I: How do the health workers ensure that what is written in the guideline is followed?
R: They know when they give me the work, I do it and end where I can manage and then report back to him or her.
I: Do they come to supervise you?
R: No. I just write on the documents what I have done.
I: Then what happens when you write them?
R: They come and ask for them to look at them then we keep.
I: You as CHW, are you given any incentive when doing hydrocele services to improve on service delivery?
R: When the university came, we were given incentives, but since it left there is nothing being provided to us.
I: What incentives where you given?
R: We were given t-shirts, bags.
I: What else?
R: That is all because the dishes and soaps were for the clients for hydrocele/ elephantiasis.
I: You were not given any money?
R: No, unless when we attend a workshop.
I: After going for the workshop were you not given anything?
R: We were given after the workshop. Though that was just for the workshop.
I: Okay, because there are times when you might be given money to use when you go in the field. Was that what happened?
R: No. Unless there are clients you are supposed to take to the hospital. If you have 3 clients, you are given k600 which is K200 per client.
I: Do you collect any data on hydrocele services that are being utilised within your community?
R: I have been idle for 2 months but previously I have been collecting data on hydrocele.
I: What was your role?
R: I used to remind the hydrocele patients on how they are supposed to be living and how they can go to the hospital when they see the situation going worse. Then I also used to assess if there are people hiding their diseases.

Files\\IDI - CHW - Mangelengele - § 3 references coded [ 12.54% Coverage]

Reference 1 - 4.18% Coverage

I: Even though you don’t have proper equipment, are there people trained to handle any hydrocele cases like surgeries?
R: No. But those are here are enough to screen, advice and give pain killers and nothing else
I: Alright, so what is needed at this facility for you to be able to offer hydrocele services apart from lack of equipment?
R: We have no trained personnel for hydrocele surgical procedures and this place is small at this clinic, there is no space for a theatre. The machines for using when operating on patients we do not have. We do not have electricity too for those machines to be able to work.
I: So if those things can be made available, can you manage to provide full hydrocele services for hydrocele patients?
R: Yes, we can manage because we came here to help people for them to get better.

Reference 2 - 5.94% Coverage

I: Do you as Community Health Workers and Assistants use guidelines or manuals as you help to implement hydrocele services?
R: We have posters, they are there, and we stick them. We have pictures that show the condition, and there are papers in Nyanja that talk about the diseases, so that when we are teaching them, they understand what we are talking about by seeing the pictures and papers.
I: Are they just pictures or there are other things inside?
R: They are written inside and they have pictures. Others have pictures inside and a bit of explanations inside.
I: Is there any way that they ensure that the practices in the guidelines being followed?
R: Yes, because they ask what programme it is for and why, so that they know whether we know what we are doing or not. When going, we tell them where we are going and why I’m going and when we come back, we report what we have done on that particular day.
I: Are there times when you go for outreach programmes and you integrate for hydrocele issues or topics?
R: Yes, a lot of times, especially when there are household visitations, you get a chance to teach the topic depending on the population.

Reference 3 - 2.42% Coverage

I: What of how we can improve on the service delivery for hydrocele patients at a facility?
R: If there is someone specific looking at the conditions can be okay. It can help because when someone comes, it will be an express service like the way ART does it instead of making lines. So at least at each facility, there should be someone trained to handle issues for hydrocele patients so that the clients should not be suffering so much as may be the case now in some areas.

Files\\IDI - Chairman - M - Mandombe - § 2 references coded [ 11.36% Coverage]

Reference 1 - 4.10% Coverage

I: How does the patient know that the health workers will be coming?
R: When you have a medical problem health workers will find you wherever you are they don’t fail they even go around the homes.
I: Does this health facility have enough equipment to help hydrocele patients?
R: No. This clinic doesn’t have enough equipment.
I: The clinic doesn’t have equipment?
R: No. It doesn’t have.
I: Are there things you say are lacking at this clinic?
R: There many things that the clinic is lacking.
I: If the hydrocele condition of a patient cannot be handled here, do you have referral services for the patient to go access hydrocele services elsewhere?
R: Yes. If the problem the hydrocele patient has cannot be handled here they write the patient a referral letter to go to the hospital for further treatment.

Reference 2 - 7.25% Coverage

I: Alright. Does that happen at all clinics or it’s just this clinic?
R: Since government clinics are the same and even at the district level it means this happens even at other clinics.
I: When you look at the clinic’s building what equipment do you think are not here?
R: You mean the equipment which are lacking?
I: Yes. The needed equipment to help improve hydrocele services.
R: There are many things which the clinic does not have. For instance, the beds and mattresses are not enough, similarly there are no adequate blankets and medicines. It is for this reason you find that a patient is just given a prescription to go buy the medicines for himself.
I: Is there enough number of rooms here?
R: No. Rooms are not enough.
I: So, do you have enough health workers or community based volunteers especially those helping deliver hydrocele services to the patients?
R: Hydrocele is not like malaria hence you would just find one or two hydrocele patients at the clinic. So, I think there are adequate health workers just the lack of equipment at the clinic?
I: So, you mean there are enough health workers?
R: Yes they are enough health workers.
I: Alright. Do you think the health workers here have the training and skills to deliver hydrocele services?
R: That I may not know because I just see that there are health workers here the clinic.
I: Would you know whether there are trained to do the job?
R: No. I don’t know that.

Files\\IDI - Com Leader - Chitope - § 2 references coded [ 4.26% Coverage]

Reference 1 - 2.13% Coverage

I: What things do you think are needed here to help hydrocele patients stop going to Katondwe hospital and Luangwa hospital for surgery? Also looking at the building facility which is here.
R: What is needed here are medical experts who know how to conduct hydrocele surgery and there also need for enough equipment. Equipment that is going to be used during the surgery should be available. Then there is also need for a separate surgical room for patients that require surgery when they come here at the facility.

Reference 2 - 2.13% Coverage

I: I mean some people when they go to the hospital just to finish the whole process it will take them maybe 2 hours or 5 hours, even 2 days while others it only take them a day everything is done. Is there a group of people you think get to be attended to faster than others?
R: Those that went to hospital brought back a good report that they were no delays undergoing surgery. Both at Katondwe hospital and Luangwa district hospital patients got attended to fast and accessed hydrocele services they had gone for in time.

Files\\IDI - Com Leader - M - Kasinsa - § 2 references coded [ 10.60% Coverage]

Reference 1 - 3.90% Coverage

I: So, if there are no equipment here, how do hydrocele patients get helped to get Katondwe Mission Hospital?
R: Here at the clinic the patient will just be given a referral letter to Katondwe Mission Hospital for surgery.
I: So earlier you said that here at the clinic they deal with fluids on hydrocele patients, do they drain the fluid?
R: Yes. They do drain the fluids form hydrocele patients.
I: When they drain the fluids from the patient’s that is when a patient is referred to Katondwe Mission Hospital?
R: Yes. When these hydrocele fluids have been drained after sometime the fluids fill up the scrotum again meaning if the patient hasn’t undergone surgery the patient may have to be drained again. That is why they advise to go to hospital to undergo surgery to clear the problem for the patient.

Reference 2 - 6.70% Coverage

I: When you look the facility, what gaps exist in the available structure to provide hydrocele services?
R: You know for equipment to be brought to this clinic, what we need the most are trained doctors with expertise in hydrocele surgery. There is a challenge when you refer hydrocele patient to Katondwe Hospital for surgery then that patient does not have money for transport, he will then stay at home and not go to access the hydrocele services he needed.
I: So, what is needed at this facility?
R: What we need the most here medical experts who can be performing hydrocele surgery. And then the necessary equipment to be used to perform the surgeries.
I: So, the medical staff here are not qualified to perform hydrocele surgery?
R: No. They are not qualified to perform the surgery.
I: But are there adequate health care providers or community health volunteers who are able to provide hydrocele services to patients?
R: No. They are just 2 health staff here with a few volunteers whom I feel none of these volunteers can do anything on hydrocele but at least the health staff can drain the fluids.
I: Does the clinic involve you to help with information that can be used to improve the implementation of hydrocele service in your community?
R: Yes, when they call us here they come to teach us how hydrocele starts so that when we go back the community we also teach our people.

Files\\IDI - Patient - Kanemela - § 1 reference coded [ 2.05% Coverage]

Reference 1 - 2.05% Coverage

R: Like I said the first help to make them aware that this is big problem and bring them together and tell them how they can access the services like drugs recommend for surgeries so that his problem can reduce.
I: So far, how satisfied are you with the services for hydrocele which are being offered here?
R: We are not satisfied with the services being provided here. As you know when a person has a problem, they have ensure that they help finish the problem of that person but here they are still many.

Files\\IDI health provider Chitope - § 1 reference coded [ 5.76% Coverage]

Reference 1 - 5.76% Coverage

I: Which kind of services do they receive here when it is confirmed that it is hydrocele apart from referring them?
R: Here it is just the relief of excess fluid because it depends on the diagnosis that is there and they are given due date to go back to the hospital for review. But for some of them, when they feel better they go for good but when the hydrocele comes back again that is when they decide to come back and seek for the service.
I: Okay, I remember you saying you refer some people, why is it so?
R: I think we lack certain treatment and speciality on knowledge how to manage hydrocele patients because the knowledge we receive is from school or maybe certain training on how to manage the patient from the time of diagnosis, during surgery and post surgery. So the only basic knowledge we have is major drugs and the things to be done. Sometimes we are not even well acquainted on the knowledge.
I: So basically, there are no equipment?
R: No.
I: And do you have any trained personnel who can manage that here?
R: No, we do not have.
I: What gaps exist in the available infrastructure here to provide these hydrocele services?
R: Infrastructure we do have maternal health clinic, it is more of the female part and most of male services have been neglected. But last year, there was a project that was going on looking at male reproductive services, so I think that one would help if we had a male clinic because even prostate cancer and other things and most of male services are neglected. So we need extra infrastructures so that our clients can come.

Files\\IDI health provider Mandombe - § 2 references coded [ 5.49% Coverage]

Reference 1 - 1.57% Coverage

I: Do these facilities have adequate equipment and supplies to provide adequate hydrocele services for the patients from the fishing and migrant population?
R: Equipment, not all facilities but supplies some have drugs and other instruments used to manage the pain. The only hospital where all surgeries are done is the Luangwa district hospital, the rest I think it is primary and it is just diagnosing.

Reference 2 - 3.92% Coverage

I: Do these health facilities and the community health workers use any guidelines to help them implement the services for hydrocele?
R: Yes.
I: Do you have manuals that you keep?
R: Yes, we have manuals that are used so we easily distribute to them. We recently distributed when we had MDA.
I: What of previously?
R: I think from the time I came, I found the manual already in place, they were there.
I: So how do you ensure that the practices and the guidelines are being followed?
R: Through monitoring of our community health workers, when we tell them to say we want them to do this, we make sure we follow up and monitor them. We tightly monitor them to make sure they are doing the right thing.
I: So, are these community health workers and CBDs provided with any incentives for them to be motivated?
R: Yes, especially for hydrocele, there are incentives for them which the District Health Office provides for them.
I: Please describe what incentive?
R: They are usually in monetary form. But others are given T-shirts.

Files\\IDI_ Health Provider Kasinsa - § 3 references coded [ 9.44% Coverage]

Reference 1 - 2.64% Coverage

I: You earlier mentioned about you not having necessary equipment with regards to operations of hydrocele conditions. What do you think are some gaps that exist in the available infrastructure like here?
R: With the available infrastructure we are a bit better here, they have been renovated, but I think it can be issues of human resource, no lab technician and the lab, we may need electricity, for now we improvise sterilisation of equipment which is not up to standard. We might also need beds. We have started with a bit of renovation but there is still work for us to do operations safel

Reference 2 - 1.52% Coverage

I: Any other thing that is needed to provide hydrocele services from here apart from these?
P: I think there is need to train more people in the community, as for now, there is only one person covering 2000 people and the target group can be about 500. So, I think we need more people to identify the clients so that they can be coming to the centre.

Reference 3 - 5.29% Coverage

I: Do the Community health workers use any guidelines or manuals to help them implement the hydrocele services?
R: Yes, during the last campaigns they were given booklets on how to identify cases and how to counsel them to the health centre.
I: How do you ensure that the practices in the guidelines are followed?
R: During quarterly meetings, we used to have refresher were we would talk about what is needed, how to identify this and that and just interactions with CBVs to see if they were doing the right thing.
I: What form of supervision do you do to ensure that the work is implemented?
R: We do that when we go for outreach services to see how they are interacting and providing that service.
I: Are the Community Health Workers provided or receive any incentive for them to improve on their performance to implement hydrocele services?
R: As of now, we do not have a hydrocele specific programme. We try to integrate programmes as much as possible. So if there is support to the CBVs like HIV /AIDS, we try to motivated them were they are given bicycles, we try to motivated them. But at the moment, there is no hydrocele project running which can provide some incentive to such workers.

Files\\PATIENT 2 - § 1 reference coded [ 3.58% Coverage]

Reference 1 - 3.58% Coverage

I = Have you ever taken part in any program of elephantiasis
R = Yes I did okay like when they came out you can be helped for them to go back inside, even at the hospital they do know how to operate on them and put them back in their position so that you also be a person.

Files\\PATIENT 5 - § 1 reference coded [ 3.68% Coverage]

Reference 1 - 3.68% Coverage

= or just about your life, how are you feeling now?
R = I am just feeling very well because when the health people come here to help me medicine and gave me money for transport, we went twice the third time I never went I got the money and went fishing so I went whilst there I was given some medicine and I got healed, so I do not register that’s if the health workers who helped me to get healed.
