# Supplementary material for: Applying community health systems lenses to identify determinants of access to surgery among mobile & migrant populations with hydrocele in Zambia: A mixed methods assessment
Source: PLOS Glob Public Health. 2023 Jul 18;3(7):e0002145. doi: 10.1371/journal.pgph.0002145 (PMC10353788; doi:10.1371/journal.pgph.0002145)
Supplement: S3 File — Data collected and reported in the manuscript. (ZIP) [file pgph.0002145.s003.zip › S2. Datasets/Programmatic lens/Affordability.docx]

Files\\COMMUNITY HEALTH WORKER 1 - § 3 references coded [ 7.14% Coverage]

Reference 1 - 0.74% Coverage

R= When we had a program, we use to give the transport money, to use to when going to the hospital
I=You use the give them transport
R= Yes

Reference 2 - 4.91% Coverage

I= Okay, so this program, of hydrocele do you receive some funds for this program to go forward?
R= Yes we used to receive
I= Where was if coming from?
R= The organisation that funded the same program.
I= which organisation?
R= the same one under university.
I= under university
R= yes
I= I have forgotten, the issue of money no, we just know what you receive we did not know how much it was.
R= okay, do you know how much you use at this post.
I= I heard bout but I do not know may be because of the age I have forgotten.
I= Okay, do you know how these people pay in order to have or recive help.
R= Nothing
I= Nothing
R=Yes
I= Okay, what about those who look for them do they receive some money?
R= Yes we use to receive some money when we have meeting and we tell them how we have moved, we use to receive some money/
I= Okay, what was that money for?
R= It was money to use when going in the field.
I= Poor going in the field?

Reference 3 - 1.50% Coverage

R= Okay, what do you think would be the major barrier for you to go forward with your work mostly.
I= Is work needs you to do something but if the money is not enough it can delay the progress of the program to go forward.
I= Okay, so money is the most difficult thing in most cases?

Files\\COMMUNITY LEADER - § 1 reference coded [ 6.87% Coverage]

Reference 1 - 6.87% Coverage

I = Okay, is there money needed for those people going in the community to look for hydrocele patients? is there any money charged so that they can receive the help at the hospital?
R = At the hospital?
I = Yes
R = No
I = What about the same patients, do they pay an amount for them to be helped, do they pay?
R = Like at Katondo hospital we need money for transport and there also charges at the hospital.
I = Okay, let us talk about the facilities or clinics that are here, do they manage all the patients in this community?
R = Yes they can manage but they always complain about shortage of drugs.
I = What?
R = they always complain about shortage of drugs in the clinic.

Files\\COMMUNITY LEADER 1 - § 3 references coded [ 9.44% Coverage]

Reference 1 - 3.95% Coverage

I = Okay tell me about the fishermen and the migrants, what challenges do they fall to find.
R = They problem is that here we are too many and if they go to Kalondwa they are scared of paying.
I = At Kalondwa hospital, where is it?
R = That’s our hospital, it’s a mission hospital.
I = Okay, so making them pay is the problem or challenge to them?
R = If they want an operation especially those from Zimbabwe or Mozambique for them to find help they have to pay for the books and for the operation.

Reference 2 - 3.77% Coverage

I = oaky, is the same for fishermen and migrants, do they add a voice to this same programs of hydrocele?
R = Here are just few and no one goes fishing unless at the Boma, so here we are few.
I = When you look at the fishermen, migrants or business people, what do you think is the problem that they do not take part in these programs in many cases?
R = Because the migrants do not put effort to the disease or problem that they have what they know is just business that’s all.

Reference 3 - 1.72% Coverage

I = Do these patients of hydrocele pay anything for them to receive this help?
R = Yes patients of hydrocele as they return from the hospital are given something for their transport to reach where they are going.

Files\\HEALTH PROVIDER - § 2 references coded [ 4.16% Coverage]

Reference 1 - 1.88% Coverage

=so do you like the care givers and hydrocele patients themselves pay any cost to reach out the service.
R= no
I= they are no cost inolved?
R=like the one we had last time they where cost involved to their side but to those people who came where responsible to pay everything including transport funds.

Reference 2 - 2.29% Coverage

I= okay is there any gaps that you feel like this gap if it existed it will work out smoothly?
R=Transport
I= okay why do you say saw?
R= mostly we donot have transport, if you refer a patient they have to look for their own transport.
I= those healthcare and community based volunteers the ones who are able to provide services to hydrocele patients, are there enough?

Files\\HEALTH WORKER 2 - § 3 references coded [ 9.80% Coverage]

Reference 1 - 1.64% Coverage

I = Okay, is that all some of the barrier? That you experienced
R= And the other things I hear from the other health facilities, they get to pay if they have to access services from and there here and open up, is very difficult other they do not have money so they feel like when they came here, we are going to request for money from them.

Reference 2 - 1.91% Coverage

I= Okay
R= So they fail due to trance
I= okay are there any money from requested from them to pay for hydrocele services?
R= not really hydrocele but anything that they come, if they just come to seek for health intercession any health facility I hear they used to pay I do not know if now they pay, but long time they used to pay a K20.00
I= K20 for what?
R= for you to access health facilities

Reference 3 - 6.25% Coverage

I= so how is the implementation of services for hydrocele within the district funded?
R= as for now there is no funding
I= There is no funding
R= No
I= Who is supposed to provide the funding?
R= Am not so sure, because last time we had the angels who come.
I=would you know how much was funded was given in the last two years?
R= No
I= You don’t Know
R= I wouldn’t know
I= But would you know what proportion of funding was given?
R= Which ones.
I= To the ones which you are doing the program
R= The neighbourhood health committee
I= yes
R= May people where just given K50 per day just like lunch allowance, yes
I= That includes even the right one population?
R= Yes, not just those people who go to identify the people with hydrocele, they are the ones who were paid.
I= okay is there a cost that attached for people who have hydrocele to be like helped
R= A cost
I = a cost yes
R= I may not know
I= okay is there a cost for you who is giving care to that patient that is required.
R= no
I= No
R= we do not require any cost
I= okay
R= unless those that goes in the field, those that go for identification.
I= so what is in your own thinking, are the equipment that are here in lwanagigwa able to cater for all hydrocele patients?
R= okay, the facilities, but what is not enough are the resource.

Files\\Head Clinical Care LDH - § 5 references coded [ 11.15% Coverage]

Reference 1 - 2.70% Coverage

I: Since they are usually referred to here, do you think there are specific categories of hydrocele patients who are able to access the services more easy than others?
R: Yes, it is true. Actually because there are those that come from far areas and you find when they go to these health posts, they have to find their own transport to come here which is costly, so some are lucky because they stay near to the hospital while some stay far from here and for those who come from far, it is very difficult. When they come, you find that it is too late because you find that the hydrocele has grown very big.

Reference 2 - 4.37% Coverage

I: Why do you think could be the reason as to why some hydrocele patients especially fishermen and migrants fail to access these services for their condition?
R: For the fishermen, I would say there are on the river 24/7 and they always want to support their families and have an income at the end of the day, the only time they find to come here is when things are very bad. The other thing I see is lack of sensitization. There are those who have large hydrocele and those with smaller hydrocele, so for the smaller hydrocele, there are no signs and they think it is normal. There are even traditional beliefs that cause them not to come to the hospital, so we had cases where a patient comes, I examine the scrotum and testes. For the migrants, the thing is the problem they find is they are supposed to pay something if the operation is done on them. But you know how it is, some of them come from very poor backgrounds and fail to access this service because of this reason.

Reference 3 - 1.43% Coverage

: Like you said, they pay a certain amount. What is that amount for?
R: That amount is for surgical items that are used during the operations. Because we use a lot of, antibiotics, so we try to help ourselves as a hospital. The charge is not very big, it is a small charge so that as a hospital we do not run out of medicines.

Reference 4 - 1.52% Coverage

I: So, is it the same with fishermen?
R: Zambians do not pay, the only people that pay are foreigners. The only time that Zambians pay, is when they do not pass through the centre, but if they come to the hospital with a referral from the centre, they are not charged. If they do not pass through the centre, they are charged a k150 as by pass.

Reference 5 - 1.13% Coverage

I: What else was given to the patients?
R: Transport refund was the only thing given.
I: Any other materials?
R: The cleaning materials to clean their wounds, school of Public health procured some and the patients that were discharged were given.

Files\\IDI - CBV - Kasinsa - § 2 references coded [ 4.56% Coverage]

Reference 1 - 2.77% Coverage

I: What kind of help did they offer?
R: The help they offered was for example, when someone has hydrocele and has been referred to the hospital, they used to worry a lot about transport money to go to the hospital. But when that organization came, they used to provide transport money and they used to pay everything for the clients and they would just receive the services when they reach the facility. The other thing they did was to give knowledge to the CBVs to sensitize to other community members.

Reference 2 - 1.79% Coverage

I: You talked about people paying. Are hydrocele patients charged when they go to the hospital to access hydrocele services?
R: Yes.
I: Is it for hydrocele patients only or even other diseases?
R: There are services that involve certain fees like scanning, x-ray and if such is required for hydrocele then you need to pay.

Files\\IDI - CHW - Mangelengele - § 3 references coded [ 8.05% Coverage]

Reference 1 - 3.35% Coverage

I: You talked about charging people, those are hydrocele patients? Are they charged when they go to the hospital?
R: Yes, they are charged for operation and they pay a bit of money, it is not for free.
I: How much is it that they are charged?
R: I do not know how much I can lie.
I: Do you know what is it for?
R: No I can lie, we just hear that they have gone to the hospital due to hydrocele. What I can say is that even scanning is k10 at the hospital.
I: Whether you are Zambian or migrant?
R: I don’t know about the others, but maybe it is more than that when you are not a Zambian, as in the fee, but all we know is that they pay because they have hydrocele problem.

Reference 2 - 1.21% Coverage

I: Can their economic status be another reason that makes it difficult for them to seek health services for their condition?
R: To some yes but to others money is not an issue, they just have other reasons for not coming to the clinic.

Reference 3 - 3.50% Coverage

I: Okay, what is your recommendation on how we can integrate services for hydrocele patients into the community health system at community level?
R: My recommendation is that we do not need to stop when this campaign stops, we are supposed to continue helping them move forward. But I do not know where they are going, in terms of expenses which is a big challenge, so I do not know what help can be offered such that the people with such challenges can be helped although the campaign already finished and if funds can allow to let every go for surgery, then the conditions in the community may finish. When everyone goes for surgery, this problem may finish in the community.

Files\\IDI - Com Leader - Chitope - § 2 references coded [ 2.70% Coverage]

Reference 1 - 2.09% Coverage

I: Apart from that, would you say it is because of money?
R: Those who travel a lot have the information as they travel around to and from Mozambique and Zimbabwe. Now, the main reason they don’t want to go to the hospital like Katondwe mission hospital is stubbornness. They sometimes think when they undergo surgery business won’t go well for them. So, it is just stubbornness they instead focus so much on their businesses saying if I undergo surgery I will be bedridden and won’t be able to do business.

Reference 2 - 0.62% Coverage

I: Do people with hydrocele required to pay something to access medical services at the hospital?
R: No, they don’t pay anything it is free of charge.

Files\\IDI - Com Leader - M - Kasinsa - § 1 reference coded [ 2.27% Coverage]

Reference 1 - 2.27% Coverage

I: Are hydrocele patients required to pay for anything when they go to the clinic?
R: No. Hydrocele patient don’t pay to access hydrocele services.
I: Meaning they don’t incur any cost at all?
R: No. They don’t pay for anything.
I: What about fishermen and migrants?
R: They also don’t pay to access hydrocele services.
I: Even migrants from Malawi or Mozambique don’t pay anything to access hydrocele services at the clinic?
R: No. The hydrocele services are free.

Files\\IDI - Patient - Kanemela - § 2 references coded [ 4.56% Coverage]

Reference 1 - 1.77% Coverage

I: Any other reason why it was difficult for you to go to the hospital to access hydrocele services?
R: The other reason is sometimes I do think of going back to the hospital for operation and it requires money to pay which I don’t have. Then I may need to be admitted in hospital for some days as I may not be released immediately after the operation and then at home how will the children survive without me, so that is another challenge

Reference 2 - 2.80% Coverage

R: For migrants when they come from their country like Mozambique, I cannot explain the proper reasons why they fail to seek the services when they are here in Zambia as I know many foreign people come to seek different health services. But for those who have hydrocele I would know specifically what hinders them from accessing the services.
I: Do you think it is due to lack of legal document required to access health services in particular for hydrocele?
R: That cannot be the reason because these people who come from neighboring country they disclose for themselves that they come from Mozambique or Zimbabwe. So they pay at the hospital knowing that they are not Zambians.

Files\\IDI - Patient - Mpuka 2 - § 1 reference coded [ 5.50% Coverage]

Reference 1 - 5.50% Coverage

I: What of migrants like those from Mozambique why is it difficult for them to go the clinic or hospital for their condition?
R: Some migrants are afraid that they are foreigners and they can’t access the services while others it is lack of money.
I: So in terms of charges, do they really charge for you to access hydrocele services at the clinic or hospital?
R: When you a Zambian you don’t pay but when you come from another country like Mozambique, I am sure they pay a certain fee.
I: Do you know how much they are charged and what is it for?
R: I don’t how much they pay and why they pay that money.
I: In this community, who are usually involved in providing hydrocele interventions for patients? Be it NGOs, community volunteers or local leaders.
R: Those I know from here are who go round in the community with papers to register people and they are called volunteers.

Files\\IDI - Patient - Sinyawagora - § 3 references coded [ 6.62% Coverage]

Reference 1 - 1.32% Coverage

I: Meaning when you need to go to the hospital for your condition?
R: Yes. Because if you are to go there now, you will need to pay your own money. And when you look into the pockets you find there is nothing.

Reference 2 - 1.72% Coverage

I: So even you the local fishermen why are you failing to go to the hospital condition when you know you have this condition?
R: Personally it was money and fear of an operation but for others I don’t know because I have not seen any voluntarily going to the hospital.

Reference 3 - 3.59% Coverage

I: In your own thinking, do hydrocele patients and their caregivers incur any cost associated with accessing the services for their condition when they visit the clinic or the hospital?
R: With what I heard from those patients who went to the hospital, they said that the group from the University which came is the one which paid for all the costs. Like I said money was another barrier in accessing hydrocele services and also transport. When you have everything, if you discover that condition today, tomorrow you can go to the hospital for help.

Files\\IDI health provider Chitope - § 1 reference coded [ 2.62% Coverage]

Reference 1 - 2.62% Coverage

I: Among the marginalised population reached were fishermen and migrants also covered?
R: I think most of the hydrocele clients have history of fishing and going across for farming activities.
I: When the people go to the facility or hospital for hydrocele services, do they incur any costs?
R: Last year when the study was being done they were not paying anything. The other client I remember who felt free to come and talk to us after the project was over, just came when the surgery was done and I think it was just the registration fees which was charged.
I: How much is the registration fee?
R: When you come with a referral from the hospital, they just give you a bill for K10 and that is usually for scan.

Files\\IDI health provider Mandombe - § 2 references coded [ 3.95% Coverage]

Reference 1 - 2.79% Coverage

I: So are free services extend to local people only or even the fishermen and the migrants?
R: This service is free as long as you are a Zambian despite where you are coming from.
I: What about the migrants or come from other countries?
R: We charge them but not the amount they can fail to pay. We charge about k40.
I: What is that amount for?
R: I think that is a medical fee, I don’t know how I can explain it, I can say it is mandatory fee for foreigners, I do not know which category it falls under but any foreigner is supposed to pay that and not that it is because they will be going for surgery for hydrocele no, it is paid by all foreigners whether you are going for eye surgery or not, rather it is regardless the illness they come with.

Reference 2 - 1.17% Coverage

I: Are there any barriers that hinder effective participation of these fishing populations?
R: One of them is economic issues for migrants especially, you tell someone to go to the hospital and if they are supposed to be admitted they ask who will be providing food for them despite them not being well.

Files\\IDI_ Health Provider Kasinsa - § 3 references coded [ 2.59% Coverage]

Reference 1 - 0.77% Coverage

I: Okay, so they feel they can incur costs of transport to and from?
R: Yes, and also that side, they need to take food and all those other things that are needed when admitted.

Reference 2 - 1.41% Coverage

I: Do you ask foreigners about legal requirements when they are accessing health services?
R: Yes, like for Mozambicans when I tell them that they need to go to the hospital for this condition, they get an extra charge for not being a citizen of Zambia, so they say better they do it from here so that they are not charged.

Reference 3 - 0.40% Coverage

I: So they incur some costs?
R: Yes, because if you are not a citizen, you are charged a K300.

Files\\PATIENT 5 - § 2 references coded [ 8.70% Coverage]

Reference 1 - 4.23% Coverage

I = What is the most difficult thing to people like you who have this condition to find help, what is the major thing? May be at the hospital to find help, what is the most difficult thing?
R = The most difficult thing sometimes is feeling that pain and you have no transport so you think of walking on foot and the treatment at hospital will take one to two days and then you come back home, so that one is so difficult for us., so that is the major one.

Reference 2 - 4.47% Coverage

I = Okay, on the Hydrocele disease what would you say is a major thing when it will help us, what can you advice on the major thing we should start with at the clinic.
R = The first thing is for them to test us and see if the disease is there or noe, the second one they should give us transport for our movement so that we can be well, the third one they should be giving something so that you are able to use it, because just leaving us like this is not good. So this is the major one.

Files\\PATIENT 7 - § 1 reference coded [ 1.50% Coverage]

Reference 1 - 1.50% Coverage

I= Tell me more about those fishermen who are suffering from this disease, why is it so difficult for them to go to the clinic to find help
R= Money
I= money
