# Supplementary material for: Applying community health systems lenses to identify determinants of access to surgery among mobile & migrant populations with hydrocele in Zambia: A mixed methods assessment
Source: PLOS Glob Public Health. 2023 Jul 18;3(7):e0002145. doi: 10.1371/journal.pgph.0002145 (PMC10353788; doi:10.1371/journal.pgph.0002145)
Supplement: S3 File — Data collected and reported in the manuscript. (ZIP) [file pgph.0002145.s003.zip › S2. Datasets/Programmatic lens/Appropriateness.docx]

Files\\COMMUNITY HEALTH WORKER 1 - § 3 references coded [ 6.77% Coverage]

Reference 1 - 0.79% Coverage

I= Is there anything else?
R= No it was just transport but they are operated on, they use to be given soap for washing the wound, a dish and a towel.

Reference 2 - 3.24% Coverage

I= Okay, and can you talk me the problem that your face concerning the fishermen and the migrants about the issue of hydrocele
R= People like those coming from Mozambique
I= Yes
R= Those people are very difficult because they stay in other areas you may find him finishing after that he will go back, so it’s difficult even if you identify that person for you to follow him up in another country
I= Okay for you to cross #
R= Yes, so that you get that person, when there in our side and you happen to identify and talk to that person it is so easier but when they go it is because so difficult to trace them.

Reference 3 - 2.74% Coverage

I= okay something you can mistake if with hyner
R= yes
I= okay, now you if someone has not gone to the doctor how would you know or tell thatv this is not hydrocele but hyner
R= us we use to ask them when they say no stomach is paining then we ask him about his testacies, if he says is going inside then we will know thatv its hyner, that’s the only difference.
I = okay meaning hydrocele they do not go inside.
R= yes they do not go inside, but they start being big out outside
I= okay, so let us talk about COVID now.

Files\\COMMUNITY HEALTH WORKER 2 - § 5 references coded [ 19.05% Coverage]

Reference 1 - 2.35% Coverage

I= okay, but how do you think it’s like that only those whom you visited are able to find help.
R= it’s like that because these have got the information whom we do visit compared to those whom we have not visited and they know very well about this disease and all beliefs that they have that this disease come by this and that they remove them out, they even know that we can only find help from that.

Reference 2 - 5.08% Coverage

I= okay, so what do you think those migrants and fisherman find it difficult to have accepts concerning hydrocele?
R= to those people okay let me start with the finish men, it’s so difficult to trace them from where they do their fishing, like here, but they go and do fishing in Mozambique, we may put them on the list to be helped, by the time that help comes this person will be very far away may be in Mozambique.
I= okay
R= So for fishermen if becomes very difficult to help them, then when we look at the migrants they are same as fishermen by the time you want them you hear that they are in Zimbabwe they are the side, so they always miss out on help.
I= why do they move from one place to another?
R= Like fisherman they follow the fish and the migrants they follow business like they order shoes from Zimbabwe sale them in Zambia so they follow business.

Reference 3 - 3.20% Coverage

I= Do you have any other issue that can be the barrier which makes them not to find help
R= The other thing like those who moves from one country to another when they reach here its not their country they do not have the Zambian identity card, so they may want help put they will fail, another barrier is language you will find that he cannot understand our language in Zambia me speak English but him understand only Portuguese, so for us to understand each and that person to reach hospital it will be so difficult if will be so difficult.

Reference 4 - 3.91% Coverage

I= how is you structure or this facilities health facilities, do they manage to help everyone who is suffering from this some disease of hydrocele.
R= I can say they do not manage 100%, why I have said so sometimes these patient may go there and they say no we have given you another day which you have to come back so those people have things to do also like what we have said so this contributes to them missing the appointments, the day when the patient is ready the people of the hospital will also be busy, and you will find that they may give them one day like all hydrocele patiently should report on Wednesday then that patient on Wednesday wont be there

Reference 5 - 4.51% Coverage

I= okay, so lets us go back to the challenges of hydrocele, do have some recommendations concerning the hydrocele patients especially the fishermen and the migrants. Do you have some suggestion that may if we can do this or that if can work out.
R= for the migrants its good that wherever they go they should find it like if they are from Zimbabwe they find it in Zambia and from Zambia to Zimbabwe they find help in Zimbabwe, so that one they talk about if so much. Then another one they look at time since they are business men that why cannot they have only one to look at hydrocele because if time comes for their surgery if takes time, but these doctors have a lot of patients to attend to but if we can just have one specific for hydrocele it will be very okay.

Files\\COMMUNITY LEADER 1 - § 1 reference coded [ 3.95% Coverage]

Reference 1 - 3.95% Coverage

I = What about in this community, do you have some people who are patients who find it so easy to find help than other?
R = Yes we do have.
I = Okay who are those people who find it so easy to find help than others?
R = Those are people who have access when they come to this health facility they find help here, so that they are given help to go to the hospital.
I = What categories of people are those who find it easy than others?
R = Some are elderly others are young people who have these problems.

Files\\HEALTH WORKER 1 - § 1 reference coded [ 2.66% Coverage]

Reference 1 - 2.66% Coverage

I = okay, when we look the migrants do you receive the migrants that are living with the hydrocele?
R= Once in well
I= one in well?
R= Yes
I= do you feel like that is any barrier to her access in their services for hydrocele
R= yes now you are doing with people at are coming either Zimbabwe.
I= Yes
R= Mozambique
I= yes or Mozambique
R= so people will behave depending on where they come from,
I= yes,
R= lets easy to deal with some people coming from Zimbabwe may be because its easy because speak a bite of English and use speak English and me speak English its say to communicate, but our friendly from across if they cannot speak the local languages chikunda her, if means they come to speak Portuguese here which I have no knowledge of, so may be one barrier language.

Files\\HEALTH WORKER 2 - § 1 reference coded [ 3.89% Coverage]

Reference 1 - 3.89% Coverage

I = okay so there are these people from other country Zimbabwe, Mozambique that comes through because fish selling and trading.
R= Yes
I= What do you are some of the barriers for them to access these services like at this clinic here.
R= I think the first thing is language barrier.
I= Okay.
R= Language is very difficult for them to communicate even something like they have hydrocele they call it like it has its name in their local language, so if they come here for them just to express or explain what they have and feeling and what they have come for its very difficult and also showing if to the health worker because they are not used interacting with us so coming opening its very difficult for the, that interpersonal relationship with hearth to workers and the language barrie

Files\\IDI - CBV - Kasinsa - § 1 reference coded [ 8.91% Coverage]

Reference 1 - 8.91% Coverage

I: What recommendation would you suggest to help improve the implementation of hydrocele services?
R: Inviting us CBVs, then we go through fish camps to sensitize. This can help because when we follow them there, they will feel important that we care for them
I: Do you have any suggestion that can address challenges specifically for fishermen and migrants at facility level?
R: Any organisation, should be able to give support to the facility, because there are a lot of things that we luck, clients complain about transport money going to the hospital, but here it is nearer, they need k100 for transport to go to the hospital which they fail. So if the services can be brought closer, it can be better.
I: Is there any recommendation that you feel local political leaders can do to help address challenges?
R: Helping each other in terms of sensitizations to the community when they have their activities.
I: How we can integrate hydrocele services into community health system at community level.
R: Hydrocele services and community programmes cannot be difficult to integrate because we have NHCs meetings in the communities and we can integrate the hydrocele topics. We also have a committee that consists of 20 men in the community to discuss about GBV, we used to meet before Covid-19, so we can integrate this hydrocele programme too.
I: What about on the facility level, how can you integrate?
R: At facility level, we can integrate it during the meanings we have with headmen, NHCs and the teachers by telling them whenever they have meetings, let this be part of the topic to be discussed.

Files\\IDI - CHW - Mangelengele - § 4 references coded [ 11.49% Coverage]

Reference 1 - 2.46% Coverage

I: Do you think the fishermen and migrants have knowledge on what to do when they have hydrocele and where they can access the hydrocele services?
R: Knowing they do know that they are supposed to go to the hospital when they suspect of having hydrocele but maybe the only thing that stops them is asking them questions and maybe staying for a long time when they are admitted at the hospital and the pass might expire, no relatives to wait for them. This is mainly for migrants.

Reference 2 - 2.78% Coverage

I: There are times when you feel you can adjust for things to go well and for people to fully understand? Was there any opportunity for you to give feedback or views on the implementation process of the hydrocele services?
R: The only thing people complained about was the time they were told to go for surgery, it was a time when people where very busy with farming, so there were suggestions that next time if there is such a programme, they should not bring it during the farming season because most of them shunned away and went to farm.

Reference 3 - 2.68% Coverage

I: Are you and health care providers receive any incentives to improve the performance when implementing these services for hydrocele?
R: Yes, they give us money per day, such that even the people you are going to talk to, you leave something for them.
I: What about other times what are you given?
R: For those who used to go to the hospital it was soap.
I: What about for the rest of you as health providers?
R: They gave us the shirts that used to show and identify us that we went for the programme and not for campaigns.

Reference 4 - 3.57% Coverage

I: Do you have any suggestions that can address the challenges that patients for hydrocele face? Or any recommendation that can address the challenges at community level?
R: At community level, I can talk about the people that go to the hospital, last time there were lines and they just give them days to go there. But you find that people end up being busy with other things and fail to go back to the facility. The ques were too much long and others would go back home. If they say that people should start going for surgery, they should let them go at any time they are ready to rather making dates and they go there just to find long que. The other things were okay since they were taken care of.

Files\\IDI - Chairman - M - Mandombe - § 1 reference coded [ 2.71% Coverage]

Reference 1 - 2.71% Coverage

I: Could it be that migrants also fail to go to the hospital because for them to be attended to they need to produce certain documents when visit the hospital?
R: Yes. For instance, a patient coming from Mozambique or Zimbabwe to access medical services here in Zambia they will need to have a border-pass then that is when they would be attended to.
I: Can language barrier also be the reason why migrants and fishermen fail to go to the hospital?
R: No. Language differences cannot be a reason why someone fails to go to the hospital.

Files\\IDI - Com Leader - Chitope - § 1 reference coded [ 1.48% Coverage]

Reference 1 - 1.48% Coverage

I: Maybe they fear because they come from other countries so they cannot be attended to here?
R: That maybe true the fear of being asked questions like where they are coming from but that is just their perception because many people when they get sick, do come here from Mozambique for medical treatment and they receive it provided they have a border-pass.

Files\\IDI - Patient - Kanemela - § 1 reference coded [ 2.84% Coverage]

Reference 1 - 2.84% Coverage

I: Would you know how much they pay?
R: I don’t know how much they are required to pay. But the amount we pay as Zambians is different from what the foreigners pay.
I: So what is the purpose of paying that money? Is it for the book, checkups or what?
R: I may just say it is mainly for the service they are seeking from the facility.
I: Do you think other fishermen or migrants fail to access the services due to language difference?
R: It can be like that to others but usually even if the language may be different others still come to seek for the services.
I: Maybe they fail to come for fear of being discriminated to nationality?
R: There is no discrimination when it comes to receiving the services.

Files\\IDI health provider Chitope - § 1 reference coded [ 2.45% Coverage]

Reference 1 - 2.45% Coverage

I: Can it be something to do with them not having legal document to access these services?
R: Most of them have two NRCs and they vote from both countries. So possibly I think most of them don’t know that something can be done about it. Even us here as health personnel when we give them the drugs, possibly on history taking, if we do not know how long they have had this disease, they end up going for good because if we are to count the number of patients that have come to disclose they have this, they are less than 5. So they do not know that there are other kind of services that can be offered other than these common drugs that we give them that can at least help them.

Files\\IDI health provider Mandombe - § 5 references coded [ 11.41% Coverage]

Reference 1 - 1.68% Coverage

I: Do you think there are some reasons as to why some hydrocele patients from fishing and migrant population have difficulties in accessing these services?
R: Yes, at times the reasons maybe stigma, others may be thinking if they go to the clinic, people will know they have that problem. Others it is time management, if we book them for surgery, you find their schedule is tight because of their scheduled businesses and activities.

Reference 2 - 4.58% Coverage

I: What is that amount for?
R: I think that is a medical fee, I don’t know how I can explain it, I can say it is mandatory fee for foreigners, I do not know which category it falls under but any foreigner is supposed to pay that and not that it is because they will be going for surgery for hydrocele no, it is paid by all foreigners whether you are going for eye surgery or not, rather it is regardless the illness they come with.
I: Let us continue talking about the reason why some people do not access services at the health facility, could it be because of the language barrier?
R: They are localised and language cannot be a barrier, we have tried to make sure we fit in because at the facility, there are people who come from within.
I: What about the migrants?
R: The people from Mozambique speak Kunda, so even for them it is not so much difficult, they do not speak Portuguese, others migrated from Zambia to there so I think in terms of language barrier, the people are just from atoning Zambezi river.
I: Could it be that others do not have proper documents that is why they fail to access the services at the health facility?
R: In terms of legal documents, no. Mostly they do come and we do attend to them.

Reference 3 - 2.08% Coverage

I: Even without legal documents?
R: They get pass regulations as long as they say they are going to access medical services. Because either country is far, so our country easily understand and they give them passes, they agree on how many hours as well. If they are admitted, they are given something by the clinic to go and show the border people.
I: Maybe others they feel they can be discriminated since they are non-Zambians?
R: In terms of that no, but the people who have an experience have disseminated information on discrimination.

Reference 4 - 1.89% Coverage

I: In terms of participation rates, does this extends to the fishermen and migrant populations?
R: For those who are local, they actively participate. But we have situations where someone is coming from Lusaka to here to fish. Based on what they heard, if it involves surgery, they start asking how they will survive if they need to stay for a specific period in the hospital, because they do not have relatives, so most of them say they get some medicines. So in terms of migrants it is tricky.

Reference 5 - 1.18% Coverage

I: That is the reason why they do not participate?
R: Yes, I think it is one of them but maybe if it is a going in and out service, they easily accept if it doesn’t involve admission. The challenge comes in when you tell them they will be admitted, on that one there are a lot of challenges especially for migrants.

Files\\IDI_ Health Provider Kasinsa - § 2 references coded [ 3.40% Coverage]

Reference 1 - 1.86% Coverage

I: With regards to fishermen and migrants, do you think that it may be an issue of economic reasons?
R: Yes, that was also another reason like when you refer a person for surgery, they complain about the length of the stay, that they can lose out especially if it is farming season for migrants, that they cannot work and also issues of transportation, they might have to book a vehicle and all those issues come into play.

Reference 2 - 1.54% Coverage

I: Do you think these migrants and fishermen are unable to come to access the information because of language barrier?
R: Language here is not a very big issue because even those across the border speak Nsenga which we speak here. Here if you are not conversant in Nsenga, we have staff who can translate. On my side, language issue is not a big one.

Files\\PATIENT 1 - § 1 reference coded [ 10.62% Coverage]

Reference 1 - 10.62% Coverage

They can have this disease but they just shy going to the hospital.
I = they just do not want to go to the hospital.
R = yes
I = why do not they want to go to the hospital.
R = why do not they want to go to the hospital?
I = yes
R = I don’t know why.
I = can you tell me in this community if you have some groups who are fighting for this same disease to prevent if. What people or kind of people who are in from fighting this same disease.
R = I only knows you who have come for this program concerning hydrocele.
I You mean no one has ever come.
R = No one
I = are you sure
R = yes
I = have you ever taken part in any programs of hydrocele since it came in your community in any where possible?
R = No
I = why do you not take part?
R Why I did not take part?
I = yea, maybe I ask this way, those fishermen who are suffering from this disease of hydrocele. Why is it difficult for them to take part in these programs of hydrocele
R = some they do not stay in nearby please so they are not seen so frequently.

Files\\PATIENT 2 - § 1 reference coded [ 4.61% Coverage]

Reference 1 - 4.61% Coverage

R= Yes they do manage with the drugs that is required by the patient.
I=Is there anyone who has come to you to talk about this same disease of elephantiasis before in the past
R= to come at my home?
I= I do not know whether at home or any where you meet with that person to ask you about this disease of elephantiasis.
R= No one has come
I=No one

Files\\PATIENT 7 - § 1 reference coded [ 3.62% Coverage]

Reference 1 - 3.62% Coverage

= what would you say is the major issues that becomes as a barrier especially to you who is a patient that you do face to find help when you go to other places.
R= If we are entering Mozambique we do get help from Zambia, we go to the clinic and inform them that we need help because we are going out. So the drugs that we get should be enough to last for the period that shall be out.
