# Supplementary material for: Applying community health systems lenses to identify determinants of access to surgery among mobile & migrant populations with hydrocele in Zambia: A mixed methods assessment
Source: PLOS Glob Public Health. 2023 Jul 18;3(7):e0002145. doi: 10.1371/journal.pgph.0002145 (PMC10353788; doi:10.1371/journal.pgph.0002145)
Supplement: S3 File — Data collected and reported in the manuscript. (ZIP) [file pgph.0002145.s003.zip › S2. Datasets/Programmatic lens/Availabilty.docx]

Files\\COMMUNITY HEALTH WORKER 1 - § 2 references coded [ 5.19% Coverage]

Reference 1 - 2.66% Coverage

I= Okay, here at the health post do you have all the equipment to use for your program for hydrocele go forward?
R= Here they do not do operation we go the hospital which is very far.
I= They only refer.
R= Yes
I= So it is not enough
R=Here we do not have
I= Here we do not have, so how do you look at this for your program to go forward.
R= Because here we do not have any doctor who can look at the issue of hydrocele, is doctors are at the hospital so we just write a referee and send them to the hospital.

Reference 2 - 2.53% Coverage

I= okay something you can mistake if with hyner
R= yes
I= okay, now you if someone has not gone to the doctor how would you know or tell thatv this is not hydrocele but hyner
R= us we use to ask them when they say no stomach is paining then we ask him about his testacies, if he says is going inside then we will know thatv its hyner, that’s the only difference.
I = okay meaning hydrocele they do not go inside.
R= yes they do not go inside, but they start being big out outside

Files\\COMMUNITY HEALTH WORKER 2 - § 1 reference coded [ 4.16% Coverage]

Reference 1 - 4.16% Coverage

I= okay
R= that’s where they want to get their help than going to the clinic, that’s why you see less numbers, ifs very rear to see hydrocele patients at that hospital, but in the community there are many.
I= So are they some who went to the traditional healers and they are healed.
R= to be healed according to what we have seen when we visit them, no one is healed.
I= no one is healed
R= yes, but they say the medicine that they get if reduces the plain but they do not get healed.
I =they do not get healed.
R= yes
I= Okay, so those who come on their own how do they manage?
R= those are helped through their family members many are even at the facility till they reach the hospital for operation (surgery).

Files\\COMMUNITY LEADER - § 1 reference coded [ 2.58% Coverage]

Reference 1 - 2.58% Coverage

I = There is nothing, what about you, do you take part to look for this same disease of Hydrocele?
R = Yes, I always do go around to talk to them so that fear in them could come out.
I = Okay
R = I always go through and encourage them that they can find help.

Files\\COMMUNITY LEADER 1 - § 1 reference coded [ 1.61% Coverage]

Reference 1 - 1.61% Coverage

I = What about the equipment? Have you heard of equipments things that they use to treat this same disease of hydrocele are they enough?
R = At this clinic they are not enough.
I = There are not enough?

Files\\FISHERMAN - § 2 references coded [ 12.43% Coverage]

Reference 1 - 6.92% Coverage

I = Okay, from your own perspective, do you think that we still have some people in this community that suffering from the same disease?
R = Yes they are there.
I = What group are those?
R = the same people who have the same disease like me.
I = Those people who find it son fast to be helped than others?
R = people who like us.
I = I meant to say those people who are able to find help than others with the same disease.
R = those who have diseases like us.
I = no those who find help when they go to the clinic than others.
R = Most of us are helped just from here at the clinic.

Reference 2 - 5.51% Coverage

I = okay, what is your thinking about the clinics that you have here, are they enough to cater for all people with this disease?
R = Like our disease, they cannot manage.
I = They cannot manage?
R = yes
I = why do you say they cannot manage?
R = there is no doctor here, how can they manage?
I = okay so it the issue of the doctor only, what is the other thing which is required?
R = is for them to learn how to do it because the doctor who is here cannot manage.

Files\\HEALTH PROVIDER - § 3 references coded [ 11.07% Coverage]

Reference 1 - 4.37% Coverage

=in your own understanding do you think the facilities around this attachment area, are they enough to cater for all the hydrocele patients
R= like surgery or what?
I= yes it does not matter like surgery. I mean everything to help the hydrocele patients, are the catchment areas enough?
R= yes there are enough
I= they are enough?
R= yes
I=why do you say saw?
R= because we have 17 health facilities I think there are enough to the district but those who do like surgery there are only 2 facilities
I=okay
R=who do only surgery
I= so do the same facilities have enough equipments to cater for the service needed for hydrocele?
R=like the 2 ones who surgery `
I= yes the tools and medicine are there enough?

Reference 2 - 3.75% Coverage

R=for that one am not too sure because am not found to those facilities
I=let me ask you about the referral system, are they put in place that patients receive the most appropriate care.
R= yes
I=what systems are those?
R=like referrals?
I= yes, what referrals systems are there like for hydrocele patients?
R=oh when the patients comes here we write a referral letter so that they do not go direct to the hospital, we refer to a certain clinic, then there they refer to the hospital, but then if we see that this is an emergency whereby the patient should not be delayed we refer direct to the hospital .

Reference 3 - 2.95% Coverage

I=what recommendations would you have like implementations of hydrocele services?
R=the program should not stop but continue like last time we had about 15 clients and only 6 or 7 were worked on so to those who remained it was really a challenge, it reached at a time where they really wanted help but they could not receive it and also the issues of transport, if these people who goes in the field can be helped with transport, it will be easily for them to find these cases.

Files\\HEALTH WORKER 1 - § 3 references coded [ 6.37% Coverage]

Reference 1 - 1.76% Coverage

I=okay, so who do these people turned to go to like the patients which hydrocele, who do they go to? Possibly in this catchment area, who do they go to?
R= it of the community, it volunteered because
I= you mean community health volunteers
R= yes
I= okay
R= the community health workers because they know what these are the people that are trained to, they are the people that mobilize and try and actually try to organize whatever health service we want to give in the community the go through them.
I= okay

Reference 2 - 3.27% Coverage

R= so those tat have problems like they could contract certain community based volunteers but also we have seen we have seen those who that had this problem for a very long tie, okay for the people who could come for training for the accessing fluids have, that has been coming out from time to time till some had surgery when this program started.
I= okay, so the services, can you mention some of the services that you provide with you like all of them.
R = okay, like so the we participate mass drug administration, we help to identify the hydrocele patients and try to connect them link them to care because I think I the past there was no one who was competently enough around like to carry out surgeries but with the coming of this program we have been like a think to the patient so that they get the help but also the program come with incentives but where given to patients during the time of recoveries so that they are able to look after.

Reference 3 - 1.34% Coverage

= okay so specifically to hydrocele services is there anything is there anything that you feel you can recommend that should be improved
R= moving forward I think the way people have talked about malaria the way people have talked about TB, the way people have hear, so often on air.
I= okay
R= I think the same time and energy even are the same drive should be given towards this.
I= okay

Files\\HEALTH WORKER 2 - § 1 reference coded [ 3.29% Coverage]

Reference 1 - 3.29% Coverage

I= okay, when they come here, what type of services do you give them?
R=okay because this is health post mostly when they come here we identify them in the communities or they come own their own most of the times, we refer them to the hospitals.
I= okay
R=yes
I= here there is all you just do is refer?
R= we check the indecent of the condition if its bleeding and if it has some manner things that we can manage here, may be bleeding or the passes is coming out we try to manage those at clinical level at health post level;, then for further diarises and treatments we refer to the hospital so here we only deal with mainer things not really helping them out like treating.

Files\\Head Clinical Care LDH - § 2 references coded [ 3.29% Coverage]

Reference 1 - 1.87% Coverage

I: So, basically, these people when they have the condition within the district, where do they access services mostly, is it at the district hospital, or clinics or other places?
R: Normally, when they are seen at the facility and the health provider at the centre there has seen that the swelling they cannot manage it, they refer them to either here at Luangwa District Hospital or Katondwe Mission Hospital.

Reference 2 - 1.42% Coverage

I: Who mostly receive services on hydrocele, Malawian, Zimbabweans or those from Mozambique?
R: On average I would rate them as Zambia, Mozambique and Zimbabwe. But for Zimbabweans, the percentage is lower, the bigger chunk comes from Zambians and Mozambicans. Zimbabwe, less than 2%. These are migrants.

Files\\IDI - CHW - Mangelengele - § 1 reference coded [ 2.99% Coverage]

Reference 1 - 2.99% Coverage

I: So what kind of assistance do they get from the nurse when they come here for those who usually come here?
R: Those who come here, since we do not do any operation and we do not have what to use for testing, we just assess the situation and tell the person that what is wrong is this and we write for them a referral letter to go to the hospital where they are helped depending on the problem.
I: So, only screening is done from here?
R: Yes, here we just do screening and we give them pain killers.
I: So meaning you refer them because you don’t have proper equipment?
R: No we do not have.

Files\\IDI - Chairman - M - Mandombe - § 3 references coded [ 13.04% Coverage]

Reference 1 - 3.11% Coverage

I: Are there specific groups of people you think are able to access the hydrocele services faster or easier than others when they come to the hospital to seek?
R: The health workers are the ones who go around sensitizing people that when you have hydrocele you should immediately go to the hospital without delaying not saying you will just use traditional medicine. Also telling the patients that once you go early to the hospital you will recover fully. In the past, many people used to have hydrocele but these days people rush to the hospital for treatment hence there are now fewer people with hydrocele.

Reference 2 - 4.23% Coverage

: From your observation are there reasons many people fail to participate in implementing hydrocele services programs?
R: One reason I see is when you suspect someone has hydrocele and ask him whether he has hydrocele they do get upset ending up not receiving the help they need. Some people feel embarrassed asking them whether they have hydrocele.
I: Any other reason?
R: There is no other reason.
I: Are you satisfied with how the delivery of hydrocele services in your community?
R: Yes. I am satisfied with hydrocele services delivery in my community because people get helped by accessing the available hydrocele services. You see now we have two hospitals which are helping people unlike before when we just had one hospital attending to people that made some people feel lazy to find money to travel to the hospital.

Reference 3 - 5.70% Coverage

I: Thank you. Do you ever have chance to review or contribute your views on how hydrocele services can be improved or implemented in your community?
R: Chance can be found when there are many hydrocele patients and when NGOs come to teach people about hydrocele that chance can be found but right now there are few hydrocele patients that chance is not there.
I: So, you have no chance?
R: No. I don’t have that chance.
I: Do hydrocele patients pay anything to access hydrocele services?
R: No. Hydrocele patients do not pay anything to access hydrocele services.
I: So, you are sure hydrocele patients don’t have to pay anything?
R: I am sure they don’t need pay anything to access the services.
I: Ok. Thank you. Do the all the facilities in Luangwa district cover all the communities or there are some communities that are not covered?
R: Yes they cover. An example I can give is from Kavuramanja its 15km so if a patient’s condition gets worse the clinic will just call the district hospital and a vehicle will come to transport the patient to the hospital for treatment. That’s why I said they do cover all communities.

Files\\IDI - Com Leader - Chitope - § 1 reference coded [ 2.38% Coverage]

Reference 1 - 2.38% Coverage

I: Ok. Thank you so much for sharing that. So, where do people with hydrocele go to access hydrocele services?
R: Here patients go to hospitals like Katondwe Hospital and Luangwa District Hospital.
I: How about here at Chitope clinic?
R: When you come to Chitope clinic they will just give you a referral letter to go to these two big hospitals I have mentioned which are Katondwe Mission Hospital and Luangwa district hospital.
I: Why do you think they just give referral letters to go to these same hospitals?
R: At the hospital that’s where they perform surgery on patients.

Files\\IDI - Com Leader - M - Kasinsa - § 2 references coded [ 5.64% Coverage]

Reference 1 - 1.42% Coverage

I: Alright. Do they conduct hydrocele surgery there at Kasinsa clinic?
R: No. There are no expert doctors to perform the surgery. Here at the clinic they just drain the fluids whilst the patient waits for specialized doctors who just come here if there is certain number of hydrocele cases.

Reference 2 - 4.22% Coverage

I: When you look at all the facilities from Faira to Luangwa Bridge, do they cover for all the communities or some communities are left out?
R: All communities are covered by the facilities.
I: What about services concerning hydrocele? Are all communities covered?
R: Yes and many people know about where to access hydrocele services.
I: So, even fishermen know about available hydrocele services?
R: Yes. Even fishermen know that the available hydrocele services.
I: Now when you look at this facility, does it have adequate equipment to carryout hydrocele surgery at this point?
R: I cannot say that there are adequate equipment for surgery at this clinic.
I: So do you that there are there or not?
R: There are no equipment because when you need surgery you will be referred to Katondwe Mission Hospital for surgery which mean the clinic does not have equipment.

Files\\IDI - Patient - Kanemela - § 1 reference coded [ 2.85% Coverage]

Reference 1 - 2.85% Coverage

I: How have the fishermen and migrants been involved with regards to hydrocele programs?
R: In short I can that here in this catchment area, the programs or services concerning hydrocele started before last year in 2019 although they were not taking place so often. Sometimes let me say others do not take part because in previous people do not take that as a problem or rather a disease because even when we look back our parents had this condition but they taking it as normal when this is a problem. So even now there are people with this condition but they are taking it as normal especially when it is not giving them pain while growing, it only considered as a disease when it starts to you pain.

Files\\IDI - Patient - Kansinsa - § 1 reference coded [ 2.70% Coverage]

Reference 1 - 2.70% Coverage

I: Are there adequate health providers or Community Based Volunteers who are able to handle your condition and provide hydrocele services to the patients?
R: They are not enough because the people who deal with hydrocele, it has to be someone with the skill and experience. So these people here cannot manage to handle these cases. I think these don’t have experience hence there is need to bring staff who are well equipped and have an experience.

Files\\IDI health provider Chitope - § 1 reference coded [ 2.95% Coverage]

Reference 1 - 2.95% Coverage

I: Do you have a specific category of hydrocele patients who are able to access these services more easy than others?
R: Yes, for example, the patients that come for relief the moment the swelling study is too much, they come for aspiration of extra fluid. Yes we do these procedures but our scope of practice sometimes we refer to clinical officers, in case we do not manage, we refer them to the hospital. There are those who come after 3 months when there is accumulation of fluids, they get relief. Then there are others like the ones we identified who are quite young, like 28 to 29 years, they shun the services because they do not want people to know they have such conditions. Others were interested and became open when they heard that Timothy was looking for people with hydrocele cases.

Files\\IDI health provider Mandombe - § 2 references coded [ 4.93% Coverage]

Reference 1 - 1.71% Coverage

I: What kind of services do you usually give them with regards to their condition?
R: Depending on the severity of the condition. But if its swelling, mostly here, we rarely manage here, sometimes if a patient says they feel pain and etc, we manage. But with draining of fluids, we rarely manage it here. So here it is just pain management. Were surgery is involved or withdraw, we refer them because here it is less than a kilometre to the hospital.

Reference 2 - 3.21% Coverage

I: The health facilities that exist in the district, do they cover all the regions of the district and making it easy for people in fishing camps and migrants access hydrocele services?
R: When you look at the district, we are one of the luckiest, because we have health facilities at least in almost all the villages because when you look at the setup, all the villages are along the road. I think they just move for a few kilometres and find a facility and move further again and find another facility. So in terms of facilities been able to make sure that the general public are able to access the services I think it is okay. When you look at the fishing camp here, it is just within town, and you find there is the Luangwa Boma, Luangwa District Hospital, we also have Feira there, so just there we have about 3 facilities within the same area.

Files\\PATIENT 2 - § 1 reference coded [ 4.61% Coverage]

Reference 1 - 4.61% Coverage

R= Yes they do manage with the drugs that is required by the patient.
I=Is there anyone who has come to you to talk about this same disease of elephantiasis before in the past
R= to come at my home?
I= I do not know whether at home or any where you meet with that person to ask you about this disease of elephantiasis.
R= No one has come
I=No one
