# Supplementary material for: Applying community health systems lenses to identify determinants of access to surgery among mobile & migrant populations with hydrocele in Zambia: A mixed methods assessment
Source: PLOS Glob Public Health. 2023 Jul 18;3(7):e0002145. doi: 10.1371/journal.pgph.0002145 (PMC10353788; doi:10.1371/journal.pgph.0002145)
Supplement: S3 File — Data collected and reported in the manuscript. (ZIP) [file pgph.0002145.s003.zip › S2. Datasets/Relational lens/Social networks.docx]

Files\\COMMUNITY HEALTH WORKER 1 - § 1 reference coded [ 4.01% Coverage]

Reference 1 - 4.01% Coverage

I = Okay, what do you other in the community who helps like the pastor or other or anyone else.
R= The others who use to help, there are some who are community health workers those during teaching under five, there where there to talk to people.
I= Is there any organisation that helps
R= We only had the university
I= Okay
R= We only had the university for the past two (2) years.
I=Okay, these people on have told me, how do they help on the issue of hydrocele tell me, what kind or type of help do they bring.
R= Help
I= Yes
R= The help was to teach us how these people looks like and how we can help them.
I= Who use to teach you?
R=The way the program for hydrocele started.
I=Okay, teaching was coming from who?
R= It was coming from the university

Files\\COMMUNITY LEADER 1 - § 1 reference coded [ 6.44% Coverage]

Reference 1 - 6.44% Coverage

I = do you have some time when you have meetings to talk about these same programs?
R = Whenever we have these meeting we do talk about them and encourage people to go to the hospital those who have hydrocele disease.
When do you hold these meetings?
I = Since I am one of the headmen we do our meetings maybe three time here or at the school and we do tell our people about the facts of life and how they should live in society.
I = Do you have opportunities when you encourage people to go to the hospital those who have this same disease and after that they bring feedback, do you have same moments.
R = We do have time just like the way are doing where someone is writing and after that they go to the hospital that people will tell us that those people he/She wrote want to the hospital for an operation.

Files\\IDI health provider Chitope - § 1 reference coded [ 4.28% Coverage]

Reference 1 - 4.28% Coverage

I: Who is Timothy?
R: The community health worker, as he was looking for those clients with hydrocele, they were talking at the drinking place where they were discussing that people with such condition can be seen even as they sit, people can see that someone is not seated comfortably and people with problems should go to Timothy. Fortunately enough, the message reached those new patients and they came. The other case was after hydrocele patients were identified, the one that went to Katondwe, I think they did not do any surgical procedure nor given medicine. So the other friend was asking what was done and he was just told to sit down with his condition, so, I think even our colleagues are not free to go and look for information and come back because I think they feel there is nothing that can be done, so if more awareness is to be done, the others can come. The other one who did the surgery, he is doing fine because the wife used to complain that even during the sexual activities, he could not perform, but now he was okay he even told us that if you want me go and communicate to others in terms of surgery, we can use him and he can be the testimony.
