# Supplementary material for: Applying community health systems lenses to identify determinants of access to surgery among mobile & migrant populations with hydrocele in Zambia: A mixed methods assessment
Source: PLOS Glob Public Health. 2023 Jul 18;3(7):e0002145. doi: 10.1371/journal.pgph.0002145 (PMC10353788; doi:10.1371/journal.pgph.0002145)
Supplement: S3 File — Data collected and reported in the manuscript. (ZIP) [file pgph.0002145.s003.zip › S2. Datasets/Relational lens/Solidarity, equity and particpation.docx]

Files\\COMMUNITY HEALTH WORKER 1 - § 1 reference coded [ 3.51% Coverage]

Reference 1 - 3.51% Coverage

I= how was it use to help you?
R= yes because we use look in those books and say on such a day I talked to this one and I did refer him, then if it means I have to remind that person I have to go and remind him, on such a day you are supported to go this side and also go and tell the provider that on such a day this person is supposed to go that side.
I= so how did you sue to know that this person is suppose to go on such a day at that particular time.
R= we use to talk to the doctors and they use to give us days that on Thursday or 20 that’s wje we are going to have a say for operation. When may in the first weak we shall need 3 peope or 2 people so we use to have a question.

Files\\HEALTH WORKER 1 - § 1 reference coded [ 2.50% Coverage]

Reference 1 - 2.50% Coverage

I= the language barriers, so what happened in the case where you know that this person is illegal, like in Zambia even documentation I illegal, what then do you do as health provider yourself.
R= I think, the policy is the ministry of health is you do not turn any anyone.
I= okay
R= you attend to them
I= yes
R= is for myself personally, I have never reported to crossed the border illogically because I think it’s a long process
I= okay
R= but I think there some people that will take it up for themselves to report such but for us I think we have been told, you do not look at colour, race you do not look at the patient is coming from lets attend to them because they have presented with a problem to you . so they attend to them.

Files\\HEALTH WORKER 2 - § 1 reference coded [ 5.79% Coverage]

Reference 1 - 5.79% Coverage

I= okay what about on community level, what do you think should happened.
R= community level as I said those people we round in the field for identification, they are just a few, they only get motivated many be anomaly or once in a year. So they just needed to be supported may if there could be an NGO that can came and support them by buying a bicycles to help them.
I= what about this facilities what do you think should be done? Do not forget that we are talking about hydrocele program.
R= Now we know that the people in the village move and of these programs, we need to have a room for them so that when they come we should not mixing the because those thing are very shameful for the lackey of better words, just come to the op and be among the other people it will be very in convince for them and others will stop coming, if they could just created as a operate room for them so that. The just come and receive the service there. As you can see its very small and we had such challenges some of them could not even come after identify they have swollen testicles they cannot come and mix with these people but if they had their only room it will be easy for them to access the services.

Files\\Head Clinical Care LDH - § 2 references coded [ 8.61% Coverage]

Reference 1 - 3.93% Coverage

I: So it is the Chiefs….
R: It is the Chiefs, community health workers and the health workers themselves.
I: So do you think the same people who support or were involved in providing the hydrocele services have an influence on how these services are utilised within the district?
R: After the surgeries, the people who go in the field see the positives that come from the patients and they see that this thing is beneficial.
I: Do the patients and local communities and stakeholders do they actively participate in the programs related to the implementation of hydrocele services?
R: They do as I said, the two Chiefs are learned and very active people and oriented them, including the community health workers. So what they do is that they do disseminate the information to the people and they know that those with symptoms of hydrocele should go to the hospital.

Reference 2 - 4.68% Coverage

I: Do you think the funding you received from the University of Zambia, a portion of it that was received was used to provide services to the most marginalised population?
R: That one, so much because a lot of these clients that came received the care and that time there was no, we had a lot of medication and a lot of vulnerable people benefited from that project.
I: Does that include migrants and fishermen?
R: Yes, this includes migrants and fishermen. They all benefited.
I: Do the existing health centres in Luangwa cover all regions of the district making it easy for patients from fishing and migrant population to access health services?
R: Yes but not all the boundaries, but I would say 80%. Some are too far, like the migrants from Zimbabwe are not covered but we just accept them because they are part and parcel of us as human beings.
I: Are the villages along the Luangwa Bridge well covered in terms of hydrocele?
R: There are some, it is easy for them to come and some it is because they have to cross the river to come here.

Files\\IDI - CBV - Kasinsa - § 2 references coded [ 5.63% Coverage]

Reference 1 - 1.40% Coverage

I: What about a situation where you segregate and treat differently the Zambians and the migrants?
R: No, that does not happen. What I know in this area is when we look at the people who have hydrocele, some say they were bewitched, some say it is genetic.

Reference 2 - 4.23% Coverage

I: Do you think proportion of the funding received was used to ensure that the services provided for hydrocele reached the most marginalised population?
R: They were mixing both critical and the ones who were better. Because what it is was that they were afraid at first and they never joined. After seeing the friends benefited, that was when a lot came through and the help reached to most of them we registered.
I: Did the help that came from the University of Zambia carter for fishermen and migrants?
R: Yes they also benefited because we were not choosing who to receive the service.
I: Since you were registering the patients, did you recruit any of the fishermen?
R: Yes, the fishermen were there.
I: How many?
R: I talked to 6 and the 6th one was a fisherman.

Files\\IDI - CHW - Mangelengele - § 1 reference coded [ 4.03% Coverage]

Reference 1 - 4.03% Coverage

I: Are there specific categories of hydrocele patients who access these services much easier than others?
R: What do you mean by specific categories?
I: These are certain group or type of people who access services more easy than others.
R: So when someone comes at OPD, whether they have hydrocele or not, we do not keep anyone for a long time at the clinic, they don’t stay for a long time. We do not keep them for long.
I: Meaning you treat everyone equally not that this one is rich, this one is light, or this one is educated and so on?
R: No, it’s first come first save, unless there are those who are in critical condition. They are the ones we give immediate attention and we put them first. But the rule is that whoever comes here, they are not supposed to stay for a long time here.

Files\\IDI - Chairman - M - Mandombe - § 2 references coded [ 5.31% Coverage]

Reference 1 - 3.60% Coverage

I: What other reasons apart from stigma and not knowing the benefits?
R: Other patients think they have to pay a lot of money and if they don’t have the money the patient will fail to go to the hospital thinking they cannot afford the services.
I: Now, do you think migrant fail to go to the hospital to access available services because they fear they are foreign citizens so they will not be attended to?
R: That fear is there because some patients think that since they are foreigners no one would help them but when they come to Zambia community leaders in the villages would tell the migrants to just go to the hospital and access available services there is no discrimination in accessing hydrocele servic

Reference 2 - 1.70% Coverage

I: When it comes to migrants and fishermen, do these health facilities cover the fishermen and migrants or not?
R: As long as someone has a health problem health workers will attend to you without looking at whether you a Zambian or not they just assist everyone. At the health facility they don’t look at where someone is coming from.

Files\\IDI - Com Leader - M - Kasinsa - § 1 reference coded [ 2.18% Coverage]

Reference 1 - 2.18% Coverage

I: When you look at Luangwa district, it has different jobs people do and people come from various places. Considering that there are migrant and fishermen with hydrocele, what are the reasons why they fail access the available hydrocele services within the district?
R: I don’t have reason so far because when someone is sick they don’t discriminate at the clinic whether that person is a foreign national or not they will be attended to equally.

Files\\IDI health provider Chitope - § 1 reference coded [ 1.56% Coverage]

Reference 1 - 1.56% Coverage

I: Or maybe others think they can be discriminated due to their nationality?
R: I do not think so because most migrants are free to access services here and it is difficult to identify whether someone is a migrant from Mozambique because we inter cross some people do farming that side in Mozambique and some people come marry here and when they come, they register under the village names found in Zambia like Manyende.

Files\\PATIENT 5 - § 1 reference coded [ 5.85% Coverage]

Reference 1 - 5.85% Coverage

I= Okay, let me talk about the fishermen and the migrants, what problems do they face when they want to have these services in order for them to be helped, what is the major difficult thing for them?
R = Those people coming from Zimbabwe and Mozambique and they are found with this disease for them to receive help, they should ask first of how they can receive that help because there is no way we can help them just because they have come, they have a country where they are coming from first they should seek help in their own country unless if they fail that side that’s when they can work with their friends in here.
