# Supplementary material for: Applying community health systems lenses to identify determinants of access to surgery among mobile & migrant populations with hydrocele in Zambia: A mixed methods assessment
Source: PLOS Glob Public Health. 2023 Jul 18;3(7):e0002145. doi: 10.1371/journal.pgph.0002145 (PMC10353788; doi:10.1371/journal.pgph.0002145)
Supplement: S3 File — Data collected and reported in the manuscript. (ZIP) [file pgph.0002145.s003.zip › S2. Datasets/Relational lens/Historical analysis.docx]

Files\\COMMUNITY HEALTH WORKER 1 - § 1 reference coded [ 0.74% Coverage]

Reference 1 - 0.74% Coverage

R= When we had a program, we use to give the transport money, to use to when going to the hospital
I=You use the give them transport
R= Yes

Files\\COMMUNITY HEALTH WORKER 2 - § 1 reference coded [ 3.47% Coverage]

Reference 1 - 3.47% Coverage

I= okay, who funded this same program about the hydrocele do you know then.
R=the one who funded this same program this same program about hydrocele.
I you have forgotten?
R=yes I have forgotten but I have the T-shirt
I= Okay what is written on it
R= I have forgotten
I= okay but do you know the amount of money which reaches to your catchment areas.
R= no I do not known
I=you do not know. Okay is there any money that a patient should take for him to be helped or the money that you get as health community worker?
I= Nothing
R= yes after taking the patient there is nothing
I = okay
R= sure

Files\\COMMUNITY LEADER - § 2 references coded [ 5.70% Coverage]

Reference 1 - 2.70% Coverage

I = Okay, in most cases who is always in front to look or take these same disease to the hospital?
R = Right now we can only say its just the hospital, Like now we can say the looking like helping.
I = Are there any other people or any other group?
R = There is nothing

Reference 2 - 3.00% Coverage

I = what do you think is a challenge you face to put your thinking or ideas concerning the same Hydrocele disease?
R = The way we have been told that the mosquito is the career of this disease and we stay near the water here in Luangwa, we need a lot of drugs, at least now things are promising.

Files\\COMMUNITY LEADER 1 - § 2 references coded [ 4.64% Coverage]

Reference 1 - 1.72% Coverage

I = Do these patients of hydrocele pay anything for them to receive this help?
R = Yes patients of hydrocele as they return from the hospital are given something for their transport to reach where they are going.

Reference 2 - 2.92% Coverage

I = Si I will request for your words of recommendation towards the programs of hydrocele at this facility level.
R = What is required is for them to continue with the program of hydrocele so that people can be helped and us headmen we shall continue encouraging people to go to the hospital for operations and checks these are the words of encourage I can give.

Files\\HEALTH PROVIDER - § 1 reference coded [ 2.47% Coverage]

Reference 1 - 2.47% Coverage

R=I think here the patients we have if they are operated on and if have no one having the hydrocele it will be better
I= so what would you like to change at the provincial level what you to change just looking at hydrocele services
R= I think they should just continue supporting these people and not to stop
I=what about on the national level?
R=still the same support and motivation is all is needed

Files\\HEALTH WORKER 1 - § 1 reference coded [ 4.03% Coverage]

Reference 1 - 4.03% Coverage

I= what about the local level local structure level around.
R= local structure level that DHO, so the way DHO has relocated resources to the higher range, is it malaria is it COVID is it TB, is it HIV I think should be given the same kind of attention, you know its we should constantly talk about resource, information should be disseminated constantly, some people are now aware all the time not when we want to do mass drug administration that when you start talk about then if sound new that has come up, you know it’s a disease that if you have it,, the disease that is associated with shame deformities of gentles you are carrying a 2.5 litres in between your legs and don’t know people to know people to know, so even if people talk about is not everyone who will come out to say I have the same problem people will tend to keep quite to myself if have to die let me die inside, unless when it comes so painful that you cannot take it alone but would love to see people come through when the deformities are still mental there are easy you manage unlike when it has grown so big so much deformities so we need, coastally talk about this not just about ephantarisis

Files\\Head Clinical Care LDH - § 2 references coded [ 10.98% Coverage]

Reference 1 - 3.80% Coverage

I: Did you give them chance to give their suggestions on how to improve quality of service for hydrocele?
R: On that one, discussions were held and at present, nothing has been done. As I said earlier on that, when school of Public health left us with drugs, nothing has been done, so if those things run out then I don’t how the program will run.
I: But during that time, did the stakeholders have a chance to give feedback?
R: No, they did not. There was no any feedback that came.
I: Do you think the funding you received from the University of Zambia, a portion of it that was received was used to provide services to the most marginalised population?
R: That one, so much because a lot of these clients that came received the care and that time there was no, we had a lot of medication and a lot of vulnerable people benefited from that project.

Reference 2 - 7.18% Coverage

I: As we conclude, we can talk about how we can integrate services for hydrocele patients into the community health systems.
R: I think for me, integration of hydrocele at community is very simple undertaking, because there are those programmes that run like MCH, oral vitamin A, those are the ones that always run I think monthly. So what the district can do is to incorporate public health officers to talk about hydrocele into the community, if it is a programme that is been held consistently, it will be very easy for us to identify those people that have this condition in the community and allocating a small portion of money to the PR for the same communities. Because that is what is happening with the eye clinic, like for them, they start with community leaders and they go to talk to the people through the sensitization and most people come for eye check-ups and that can be the same with hydrocele and we can save a lot of people.
I: How can we integrate at facility level?
R: At facility level, they just have to continue talking about this hydrocele during their child health week and community meetings through the headmen and community leaders.
I: What about recommendations on how we can integrate these health systems at provincial level?
R: I think it is very easy because the problem which is there is that the condition is not very prevalent in most of the district, so now since it is prevalent, the province can integrate and give a small grant for surgical, health education to the affected district because each district has a peculiar disease in their area.
