# Supplementary material for: Applying community health systems lenses to identify determinants of access to surgery among mobile & migrant populations with hydrocele in Zambia: A mixed methods assessment
Source: PLOS Glob Public Health. 2023 Jul 18;3(7):e0002145. doi: 10.1371/journal.pgph.0002145 (PMC10353788; doi:10.1371/journal.pgph.0002145)
Supplement: S3 File — Data collected and reported in the manuscript. (ZIP) [file pgph.0002145.s003.zip › S2. Datasets/Relational lens/Multisectoral engagement.docx]

Files\\COMMUNITY HEALTH WORKER 2 - § 1 reference coded [ 5.98% Coverage]

Reference 1 - 5.98% Coverage

I= The all Luangwa
R= why I have said so there are some people who attended the meeting, chief and others but now when we come on ground level. They are not so active to tell their people to do that, for them to let people know they may have the information but fails to deliver to their people, because in the community you find out the people have no information but the headman has the full information
I= Okay so you where do you work from which area.
R= Okay come from Ludaka village but under Lwangwa boma clinic.
I=Okay
R=Yes
I=from the time his program, started, do you have some people who comes to visit and see how this program about hydrocele is going on?
R= I can say this program, the people who spear head is the district
I= district?
R= yes
I= so they come to check on this program
R= no not coming physically but we communicate with them, may be if there are some other, new case and those who remind from the other program, how are they doing so they try to test them so that they can receive help also

Files\\HEALTH PROVIDER - § 1 reference coded [ 4.55% Coverage]

Reference 1 - 4.55% Coverage

I= okay so can you tell the people or organizations that are offering services for these hydrocele patients
R= I know one that came from UNZA where Mr chileshe came here
I= UNZA?
R=yes, that the only one I know and patients were referred to Luangwa and Katondo Hospital, the district hospital
I=okay so how did they came to know and implement this same program?
R=which one? the same
I=the same people you are telling me how did they came to implement for hydrocele services?
R= because they saw that this case is growing high no wander they started implementing to prevent it, from continuing
I=okay so is there any one for example in the community around here who is also involved for providing services to hydrocele patients?

Files\\HEALTH WORKER 2 - § 2 references coded [ 9.25% Coverage]

Reference 1 - 6.46% Coverage

I= okay, twice in this catchment area are their nay persons, groups or organisation that involved or spear heading the hydrocele campaign or programs like that.
R= okay in this catchment area yes.
I= yes
R= yes because was if last year if not mistaken I think last there was an NGOs, that came they were looking for the same people with hydrocele.
I= so which kind people or person or organisation are those, can you mention them?
I= I cannot really remember because they passed through the district health office and the district health office, just communicated to us and from that time we have confided it to be on going thing we never stop it because they implemented it and created awareness in us and also created some group some groups of people in the communicate to go be going around and identify people with hydrocele.
I= but you did not know where they come from or what they represent who they were.
R= I just know that they were NGO
I= an NGO
R= Yes
I= Okay, do those had an influence in what’s happening in the community.
R= Influences as in?
I= Any, do they have specific roles responsibility or anything like that.
R= The NGOs
I= Yes the ones that come through, the ones you were telling me about?
R= those people just came for the same identification, after that they have never come back again.

Reference 2 - 2.79% Coverage

I= okay may be stake hoods like that local people communities, are able to participate in any these programs?
R= yes
I= Why do you so say so
R= Because like I mentioned earlier from that time if has been declared as an ongoing thing, so they organise some neighbourhood health committees, the community health workers, most of the times they go through villages to identify these people
I= Okay
R= Yes
I= So are these people also includes the migrants and those other traders.
R= yes any group of individually, even if they are migrants has long as are here.
I= okay

Files\\Head Clinical Care LDH - § 6 references coded [ 20.28% Coverage]

Reference 1 - 5.54% Coverage

I: In terms of providing hydrocele services to patients, who are usually involved in this district?
R: Normally, I think it is not specific, let me not lie. What happens is a centre will refer. Depending on the person found at OPD, most of the clinical officers I work with know that when a case come they would inform me and then I will an assessment of the condition, and if the person is ready for surgery just there and then, it will be done especially these people who come from Zimbabwe because once you tell them to go and come back another day, they go for good. So I tell them that prepare we will do the surgery the very day.
I: I mean the people who participate in making sure that the health services for hydrocele patients are given.
R: There was a programme with school of Public Health, it was the first study that they did and provided us with a number of materials like surgical materials, pain killers and anti biotics. So it was a substantial amount of supplies that they gave us, those supplies are kept in the theatre and they are the ones that we are using and still available because they were given to us here at Luangwa Hospital and Katondwe Mission. When they run out, we have to revert to our stock I the pharmacy

Reference 2 - 0.95% Coverage

I: Apart from the University of Zambia, school of Public health, who else are involved in the provision of hydrocele services, at community level, facility level and district level?
R: At the moment, no one.

Reference 3 - 1.99% Coverage

I: Community health workers?
R: Yes because some were trained and I have quite active ones like in my catchment area, they are 2 and remember one time they even escorted the patients to the hospital.
I: What of Religious leaders?
R: Let me not lie for that one, I am not sure.
I: Are there any NGOs that support the provision of hydrocele services?
R: There is no any NGO apart from the University of Zambia, from that time nothing has happened.

Reference 4 - 3.56% Coverage

I: Are there times when stakeholders review the implementation of hydrocele services in the district to improve the quality of service?
R: On that one no. No stake holder has ever come. The only time we saw concern for hydrocele was when the School of Public Health came. Otherwise, all the stakeholders don’t look at hydrocele to be a public health concern or emergency.
I: During the time the University of Zambia students came to the district to implement the same hydrocele, were the stake holders given an opportunity to participate in the implementation of hydrocele services?
R: Yes, they were. We were having meeting and workshops with their team and local stakeholders like chiefs, village headmen, CHW, Health workers and others for I think 3 times from 2019 to 2020.

Reference 5 - 5.76% Coverage

I: Okay, let us talk about the recommendations on how we can improve the hydrocele services in the district. So will talk about community, local political structures and facility levels. How can the challenges be addressed to improve the issue of hydrocele especially for fishermen and migrants?
R: With the community, there is an area where the fishermen always start from before they go and fish and they have an association, so if health education can be given to them on what is, what causes and what are the benefits of hydrocele can be given to them, it can help a lot. Because once we involve people, they will not be surprised if there are some success stories, we can also take to them for them to learn. And it is very easy for anyone to disseminate information to them at the harbour since that is where most of the fishermen are found. You talk to them, they understand the issues and complication and a lot will come, that is at community level. Then at health centre level, people have just been seeing hydrocele as a disease form the books. So the district office can do a clinical visit on these centres to sensitize on hydrocele, this can help a lot during their monthly visits at these centres also put hydrocele as one of the health problems in the district.

Reference 6 - 2.48% Coverage

I: Any recommendations on how we can integrate this at National level?
R: I think the advocacy has to come from the University, you know like school of Public health, has the power, resources, information to go and convince those at national level to say this is a problem that we have identified at this district and this is the recommendation we have for this district. That will be much easier, like us here, we have information but we do not use it. But with the school itself with its resource, knowledge and skills it can help us implement this.

Files\\IDI - CBV - Kasinsa - § 2 references coded [ 9.02% Coverage]

Reference 1 - 5.04% Coverage

I: So, within your facility catchment area, who are usually involved in providing hydrocele implementation for the patients?
R: I can say CBVs, Clinical Officers at the centre provide those services to people through sensitization. Traditional leaders too they do help in organizing people for the meeting and also disseminating information to the community members.
I: What about others like churches?
R: Unless we just tell them to announce for us in the church when there is an activity coming, they do that for us.
I: You talked about an NGO from UNZA as one of the stakeholders that took part?
R: Yes.
I: How did they become part of the programme?
R: They spearheaded the hydrocele services in the district.
I: And how were you as CBVs involved in implementing hydrocele services?
R: The community identified me to say since I am in the community and if I’m sent anywhere, I do deliver services nicely.

Reference 2 - 3.98% Coverage

I: Do you mainly focus on hydrocele in those meetings or it is general?
R: It is a general meeting, it is also a subject.
I: So in terms of hydrocele, how often do you talk about hydrocele in those meetings?
R: When we talk about it now, next meeting we will not talk about it? So it is not often.
I: So how many times in a quarter, half or year?
R: In a quarter, once. So for us volunteers, in a week we should visit our clients once to talk to them about hydrocele and to tell them how they can go to the hospital.
I: Would you know if there has been funding for the past 3 years to implement hydrocele?
R: No.
I: Apart from the University of Zambia, you have not been funded?
R: No.
I: Do you know how much you were funded?
R: No I don’t know.

Files\\IDI - CHW - Mangelengele - § 2 references coded [ 8.45% Coverage]

Reference 1 - 3.64% Coverage

I: So, in your area, who are usually involved in providing health services for hydrocele patients?
R: Nurses at the facility.
I: Others?
R: Community Health Workers, Traditional Leaders and SMAGS.
I: Any NGOs that help out?
R: There are no NGOs.
I: You mentioned about the project that came in 2019.
R: There was one NGO that came from the University of Zambia.
I: What kind of help did they bring?
R: They taught people at the clinic and after that, then they started teaching the community members about the disease, attending to patients, took them to the hospital for operation. At the hospital, transport refunds were given, bills were paid for, soaps were being given and everyone was thankful.

Reference 2 - 4.80% Coverage

I: Any other help?
R: They are still helping those who remained.
I: What are the roles of the Community Health Workers to ensure they implement the hydrocele services for people?
R: They help to take the word in the community about hydrocele and its implications so that everyone knows about the disease, because they are the ones found with people in the community. Then they also help to take the people who are shy but have the condition to the clinic and they explain for them what the problem is to the nurse.
I: What is the role of Traditional and Church Leaders?
R: Church leaders help spread the word to the people in the community and people listen to whatever these traditional leaders and church leaders say to them.
I: What about you, what do you do?
R: It is the same job that we also do to help in spreading the word and ensuring that people receive help from the hospital and also check on their health after an operation.

Files\\IDI - Chairman - M - Mandombe - § 1 reference coded [ 8.51% Coverage]

Reference 1 - 8.51% Coverage

I: What about at community level who is involved?
R: At community level community health workers are involved as go they around the villages teaching people about hydrocele and when they find a hydrocele patient they encourage such person to go to the hospital and seek help.
I: Who else is involved?
R: The same health workers are involved when a patient comes to the hospital or they visit the patient at home they do advise the patient to access available hydrocele services as soon as possible. Upon coming to the hospital and the problem is discovered to be big the patient is referred to the hospital.
I: Are there any NGOs you know that assist hydrocele patients or promote hydrocele services?
R: I don’t know any NGO that does that.
I: What about church leaders are they involved?
R: Church leaders are not involved either.
I: As a chairperson, is there a role you play in implementing hydrocele services?
R: We get to organize meetings teaching people about hydrocele and advising people that if you have hydrocele go to the hospital as soon as possible instead of just staying at home. I also get to invite health workers to come and teach people about hydrocele.
I: What other roles do you play?
R: There is no other role I play.
I: You don’t play any other role?
R: No. I don’t play any other role.
I: Who else is involved in promoting hydrocele services delivery in the community?
R: The community health workers are also involved because as they go around the community if they find someone with hydrocele they do advise such patient to immediately go to the hospital to access available hydrocele service so that the patient’s problem is cleared completely.

Files\\IDI - Com Leader - Chitope - § 8 references coded [ 19.23% Coverage]

Reference 1 - 2.11% Coverage

I: Now who is involved in helping to provide services for hydrocele in your area?
R: At Katondwe mission hospital and Luangwa district hospital the nurses, clinical officers, doctors all work together to provide services for hydrocele patients. When we come to the community level we do sensitize the headmen to gather people together and teach them about hydrocele. Then the community health workers and those in neighborhood health committees also work together to help provide hydrocele services.

Reference 2 - 2.25% Coverage

I: How about church leaders?
R: Yes. Church leaders also get involved.
I: How about local political leaders in your area?
R: I have not hear any political leader getting involved.
I: Are there organizations that are involved in providing intervention for hydrocele services?
R: When it comes to hydrocele I just know the district health team.
I: There is no organization?
R: No, there is no organization but in 2019 and 2020 we used to work together with Professor Michelo from the University of Zambia (UNZA). He come here about three times.

Reference 3 - 2.71% Coverage

I: What help came from UNZA?
R: We used to meet at Luangwa district Boma discussing ways on how to help people with hydrocele. From there we agreed that we go the community sensitizing people so that people with hydrocele should start going to the hospital to undergo surgery.
I: What was UNZA bringing here?
R: They used to bring information to the people on hydrocele through the headmen and community health workers since the headmen and community health workers are the ones who live with the people in the community. It was like a discussion where we would find ways to help these people with hydrocele to access hydrocele services from the hospital.

Reference 4 - 3.03% Coverage

I: Thank you so much. When it comes to hydrocele interventions for those of you involved what exactly do you do? What are your roles as headmen?
R: In past we used to have meetings as headmen and being senior headman myself we would talk about hydrocele. Then each headmen would go to his village tell his subjects about the benefits of going to the hospital to access hydrocele services.
I: How about church leaders?
R: If they are in attendance we tell them to talk about hydrocele to their church members but we advise it is done in confidence not announcing in church as it brings embarrassment to those who have hydrocele. So, the church leaders have to know those with hydrocele then talk to them in private about their condition.

Reference 5 - 2.56% Coverage

I: Now, there different programs concerning hydrocele do you often participate in these programs?
R: If they tell me that on such a day there will be conducting surgery we do make sure we inform people about such a program. Some people will accept to go for surgery while others will accept but will change the mind at the last minute and not undergo surgery.
I: Let us talk about active participation, how active do stakeholders participate in the hydrocele programs?
R: Like I said church leaders are not very actively involved but headmen and community health workers are actively involved in hydrocele programs.

Reference 6 - 0.82% Coverage

I: How about the migrant do they also attend such programs?
R: Some fishermen and migrant do attend meetings concerning hydrocele when they are come back from ordering fish down the Zambezi River.

Reference 7 - 4.51% Coverage

I: Now with regards to hydrocele programs being implemented here, are you satisfied with the program?
R: Yes. This program is very good.
I: Has it helped people?
R: Yes it has help because hydrocele is bad disease. That is why this program should continue to help people because not everyone will accept to undergone surgery at the same time. It takes time to convince some people to access available hydrocele services.
I: As a community leader, have you ever been given a chance review the implementation program concerning hydrocele and express your views on how to improve the quality of services concerning hydrocele program?
R: Yes.
I: What opportunity was that?
R: I was given chance for instance in 2019 and 2020 in the meeting when I was working with professor Michelo, I was given chance to first gather people together in community then I start teaching them about hydrocele. Thereafter, I would ask all those that have hydrocele not to believe people who say when you undergo surgery you will die. That was the chance I was given in the community and to this day we still do that.

Reference 8 - 1.24% Coverage

I: What about other leaders?
R: Other local leaders like church leader should not wait until church day to talk hydrocele patients. When they go to the harbor to buy fish and meet such people they should talk to them nicely telling them benefits of going to the hospital to access hydrocele services.

Files\\IDI - Com Leader - M - Kasinsa - § 2 references coded [ 3.11% Coverage]

Reference 1 - 1.61% Coverage

I: Now, who is involved in providing hydrocele intervention for patients with hydrocele in your community?
R: In the community we do have volunteers and headmen. We do go for workshops where they teach us how to skillfully talk to patients about benefits of going to the clinic and overcoming shyness as they live with hydrocele.

Reference 2 - 1.50% Coverage

I: How did you get involved in the hydrocele program?
R: When health workers taught us that when people are getting sick in village especially the youth, there will be no development in the area. So, that is how I decided to get involved to help people by influencing them to go the hospital for their condition

Files\\IDI - Patient - Kanemela - § 2 references coded [ 3.82% Coverage]

Reference 1 - 2.58% Coverage

I: What of the traditional or local leaders?
R: Ok here I will not lie I have not seen any traditional leader taking part in any program.
I: Don’t they offer any services like talking to their subjects?
R: On hydrocele but on other diseases like Covid 19 they do talk about that.
I: What are the main roles and responsibilities of the people you have mentioned here?
R: Their roles is that they help in creating awareness in the community. Then also helping to find a solution to the sickness that one is having. And they explain to the patient how the problem starts so that the patient can know where and how his condition came about.

Reference 2 - 1.24% Coverage

I: What about other fishermen like you and they have hydrocele?
R: They also do participate because I do remember there was a time when they were registering people with this condition, we took part in that process. We were called and they explained to us how the disease comes about and I was also there.

Files\\IDI - Patient - Kansinsa - § 1 reference coded [ 5.80% Coverage]

Reference 1 - 5.80% Coverage

I: So within your health facility catchment area who usually involved in providing hydrocele intervention for patients with hydrocele? Be it NGO, be it government organization CBVs and many others.
R: I consider help as in when you have been given something so that you can feel better.
I: If it is like that, who helps you access those items that make you feel better on your condition?
R: There are two men who are volunteers from the clinic.
I: So those are involved in providing hydrocele services. Who else is involved?
R: And the doctor at the clinic helped in draining the fluid.
I: Any other person or organization?
R: No one, those are the people I know who provides help here.
I: Are you involved in any way in the implementation of hydrocele services?
R: I have never taken part in any activity concerning hydrocele. The only thing I do is that when it starts paining, I go in the bush to look traditional medicine and use it to release the pain but it doesn’t work.

Files\\IDI - Patient - Sinyawagora - § 1 reference coded [ 4.31% Coverage]

Reference 1 - 4.31% Coverage

R: I am not sure but I hear they come from town at the University.
I: Which university?
R: University of Zambia.
I: Yes, others? You can even mention those from within the local communities.
R: I think it is just 2 that I have seen with the Community Volunteers.
I: What about the headmen?
R: The headmen also help in such a way that when there is a program, and when the headman is informed to tell the people in that community, he manages to tell his subjects. On this they really help I can’t lie.
I: What of those from the clinic, don’t they help?
R: They help by proving the information to the headmen to disseminate to the people surrounding different communities.

Files\\IDI health provider Chitope - § 3 references coded [ 10.72% Coverage]

Reference 1 - 4.21% Coverage

I: So with regards to hydrocele, who is involved in this district in terms of providing hydrocele services for the patients?
R: Locally, clinical when we come to OPD, last time more emphasis was put when there was a study by the University of Zambia, that was when most of them were been booked, taken for surgery and give soaps.
I: Just soaps?
R: There were soaps, buckets and I think they were being given some cash so that they can be using for transport as well as money for review.
I: That was from the University of Zambia?
R: Yes, it was the only organisation I heard of in the provision of care for hydrocele patients.
I: Who else apart from the University of Zambia was involved, it could be NGOs, Community Organisations, any other persons that you think were involved.
R: At community level, it is Community Health Workers who have been following them to see how they are doing. Then at facility level, it is the Clinicians when we see these patients, then at tertiary point of care is the hospital.
I: Church leaders and headmen are they not involved in providing the hydrocele services to the patients?
R: I have never seen such.

Reference 2 - 5.27% Coverage

I: How were these actors you have mentioned involved? What were their roles?
R: The community health workers are the ones who identify the patients from the Community, and refer them to the nearest health facility and then after help is given, they come back, and sometimes they do follow ups to see how the patients are doing. Sometimes there are other clients who do not want to come to the clinic, I think we have an old man in Kapete village, so the community health workers came in and say we really want to help this man but we do not know how to approach him, we are scared of him. So we advised him to use his wife so that she can talk to him so that she can explain to him that there is such a service which can be offered at the clinic, better to try him than let him continue to suffer. So they usually help us identifying such clients and make recommendations.
I: At district level?
R: They have been following up such cases at district level in conjunction with the University of Zambia. They have been following up on how the study went on, how clients were doing and if there were new clients that were available. During sensitization of health education, they encouraged us to include information related to elephantiasis and in every February, we usually have a programme where we give drugs to prevent lymphatic Filariasis that albendazole. So the district is involved possibly in the prevention of infection.

Reference 3 - 1.24% Coverage

I: So, does the participation also extend to the patients from the fishing and migration population?
R: Yes they do participate.
I: Whenever they are participating, do the migrants and fishermen who have hydrocele participate in these programmes?
R: Actually, the study that was conducted last year, a lot of them participated.

Files\\IDI health provider Mandombe - § 3 references coded [ 14.25% Coverage]

Reference 1 - 3.60% Coverage

I: Not the names, it could be an NGO, what kind of NGO, is it people who are at the district?
R: Well, if I’m not mistaken, the one we had last year, I don’t know if it is early this year when we had Mass Distribution of DEC, we had Akross who came in which is an NGO which supported the programme of hydrocele. In terms of management of hydrocele we have Medical Licentiate which involves surgery after identification by the CBVs, then refer them to Luangwa general hospital.
I: So, there is Akross, Medical Licentiate at the hospital and CBVs. Who else is involved?
R: Even us health workers and Environmental Health Technicians EHTs.
I: Who else?
R: Even traditional leaders we involve them, Headmen, especially when we have Mass Drug Administration, we involve everyone church leaders, nurses, CBVs, nurses and everyone. It is a multi-sectoral programme, we approach it holistically to make sure almost everyone access the services.

Reference 2 - 6.14% Coverage

I: So do you as health workers here and others have an influence on how these services are been delivered or utilised within the district?
R: Yes. The influence is there since we are the people on the ground and we know if it involves stigmatisation, so in terms of approach, sometimes you find that certain people fail to open up and they might say okay, the people from the district are going to come. But we usually advise that if you people from the District you come and enter the community without us who are based here in this catchment area, it will be difficult for you to talk to the people better you involve staff at the facility because we are used to them and we know how to talk to them so it becomes easier. So we have an influence on that, we also advice that at this time you cannot find these people to talk to them because we know the setup of these communities.
I: Are the local communities, patients and stakeholders able to actively participate in the implementation of the programme activities in relation to hydrocele?
R: The local communities, leaders and others, they are very much helpful in terms of these services especially were the consumption of the services is involved. Even before we implement the programmes, we call the church leaders, community leaders, traditional leaders to sit them down to sensitize to them because if we just go like that, they will start wondering what is that, but before that, we make sure we get their permission and educate them on what we want to do, they even have meetings without us and go and educate the people according to what we told them.

Reference 3 - 4.50% Coverage

I: So, this is done at the facility level. Do you hold meetings?
R: Yes and meetings are usually there.
I: What about at district level, do you have an idea of what happens?
R: On district level, that one, I can say not that much because mostly, it flows down, so the district just brings the programme and we follow what they tell us. So commenting on district level, I cannot comment much because I’m not part of the committee. The district comes and tells us what they want us to do then we think about it. If it is okay, we go to the community and take it.
I: So, you have said at district level, you think it is not that much?
R: The meetings are there but in terms of how or maybe how they make resolutions, that is the part I’m saying we just receive the final decision and we are not involved in the process. Unless if they call for a meeting at district level and they involve us. But there are situations where they plan as a district and they just come to information us. So they do have the meetings and they inform us.
I: How often do you usually hold these meetings here?
R: We have schedules for health education, those educational schedules, sometimes we have them twice in a month.

Files\\IDI_ Health Provider Kasinsa - § 2 references coded [ 5.29% Coverage]

Reference 1 - 2.83% Coverage

I: It could be health workers, government organizations, community volunteers and NGOs, so who are the people who work with you?
R: Last time we had a programme where the community health workers were told to identify the cases and those people where provided with transportation to have surgeries done at Katondwe Hospital, but since the programme ended, patients are still been identified, but when it comes to transport and other costs, if they come to Katondo for surgery, the other costs should be incurred by themselves. So there is no a specific NGO which is helping to provide hydrocele services since the programme ended.

Reference 2 - 2.46% Coverage

I: In the communities, who help out with the support of hydrocele, not necessarily financial but other ways of support to these people?
R: We have neighbourhood health committees where we do outreach programmes, we disseminate that information using CBVs. When it comes to Voluntary Male Circumcision, we also try to integrate other health issues when we go to mobilise because we usually have men and boys, so we tell them even during the VMC that if they have any other problem, they can tell us afterwards, so that we see how we can offer assistance.
