# Supplementary material for: Applying community health systems lenses to identify determinants of access to surgery among mobile & migrant populations with hydrocele in Zambia: A mixed methods assessment
Source: PLOS Glob Public Health. 2023 Jul 18;3(7):e0002145. doi: 10.1371/journal.pgph.0002145 (PMC10353788; doi:10.1371/journal.pgph.0002145)
Supplement: S3 File — Data collected and reported in the manuscript. (ZIP) [file pgph.0002145.s003.zip › S2. Datasets/Relational lens/Adaptive implementation.docx]

Files\\COMMUNITY HEALTH WORKER 1 - § 1 reference coded [ 2.62% Coverage]

Reference 1 - 2.62% Coverage

I= Okay, Let’s talk about this community here who provides services for hydrocele? who brings help.
R= Hydrocele here we have a provider if you refers a patient if found and us also we refer if we found one in the community, we also had someone who was our leader.
I= which leader
R= Chileshe
I= He was under what?
R= University
I= University of Zambia
R= Yes
I = Okay
R= He uses to come and hold meetings and he monitors what we were doing and monitors the books and the way we are working.
