# Supplementary material for: Applying community health systems lenses to identify determinants of access to surgery among mobile & migrant populations with hydrocele in Zambia: A mixed methods assessment
Source: PLOS Glob Public Health. 2023 Jul 18;3(7):e0002145. doi: 10.1371/journal.pgph.0002145 (PMC10353788; doi:10.1371/journal.pgph.0002145)
Supplement: S3 File — Data collected and reported in the manuscript. (ZIP) [file pgph.0002145.s003.zip › S2. Datasets/Relational lens/Relationships of trust.docx]

Files\\COMMUNITY HEALTH WORKER 2 - § 3 references coded [ 13.41% Coverage]

Reference 1 - 3.89% Coverage

R= yes, so at the hospital that where they do surgery but here we just refer so then they go to hospital.
I= okay, do you have some people here in the community who finds if easier to find help of this disease than others.
R= may be I just say there us a certain tribe that find it easier for help than the other one?
I= those who find it so easy to be helped than others.
R= I can only say all these people are at the same level but those who find if easy are those whom we go through your programs like this one
I= okay
R= Those at least they have information because we have visited them than those who not heard about thus those find it so difficult to have this help.

Reference 2 - 5.55% Coverage

I= do you have some guide times of how to help the hydrocele patients
R=at community level we do not have them.
I= you do not have them
R= yes we have nothing to help the patiently
I=so now how do you help the patients
R= the way it is at community level despite of any disease or sickness we just help each other, as a general help which means even hydrocele it can be found if that person is not feeling too well to go to the clinic, as a community you can even remove money for transport for them to go but its general, maternity anything we do help, we even have the board to take to the hospital and back.
I= Okay do you give anything or receive anything on the same work as CHW.
R=At community level or what
I= At any level as long as you can do the work concerning the hydrocele
R= We receive something when we go in the field as refunds for transport.
I= Something like how much
R=Like this program we have for 10days we are given K100per day.

Reference 3 - 3.98% Coverage

I= Okay, what about the provincial level what would be you recommendation for the program of hydrocele to go forward.
R= Okay I can say at provincial those who make programs for it to go forward they should consider us we go I the field a lot of people do ask us about our IDs even the t-shirts we do not they start asking us where do you come from for me to give you information about my self, because this is secret disease so if we can have those even from a far we be able to be known from a far we be able to known, so that it can even send a message to people before we even talk to them, we here given T-shirts but it was a long time age there are worn out, you see how hot it is here.

Files\\COMMUNITY LEADER - § 1 reference coded [ 9.71% Coverage]

Reference 1 - 9.71% Coverage

I= How did you become involved in this same program of going around to encourage them?
R = To start with I was suffering from the same disease and here we call it Nshinga.
I = Okay Nshinga and not Ntumbu?
R = No if you say Ntumbu people feel shy.
I = Okay why do they feel shy?
R = Because that disease is placed on the testicles and is painful, so the things go inside and become bid under your legs, so we cannot say Ntumbu is painful, so we cannot say its paining but we say Nshinga, when it reaches so big then we call it Ntumbu.
I = Okay, so Nshinga
R = yes
I = Okay tell me how you became involved.
I = so this program after they came, they were looking for people suffering from this same disease of Hydrocele, I was the first one to tell them that I have this same disease, but I have a problem that in most cases I run out of drugs, so after registering with them, then we went together and I started telling my friends that there is help this side.

Files\\COMMUNITY LEADER 1 - § 1 reference coded [ 1.27% Coverage]

Reference 1 - 1.27% Coverage

I = Do you have any challenges to the people in your community about hydrocele disease?.
R = It’s not difficult because am the headman I Can talk at anytime.

Files\\FISHERMAN - § 1 reference coded [ 7.37% Coverage]

Reference 1 - 7.37% Coverage

= so because you never went, so when did you know about it.
R = I knew about it when this man called Jesus visit me.
I = Okay this CHW
R= yes
I= when was that day when he visited you?
R = it was Monday
I = what did he tell you, do not say like a story but tell me everything
R = he told me that they’re some people who can take you that side and you can be helped, at least you can feel good, you should go to the clinic and talk to them.
I = so what happened when you told?
R = when I came back, okay let me put this like this
I = yes tell me, so what happened?
R = yesterday there was nothing that happened till you came here.

Files\\IDI - Patient - Sinyawagora - § 1 reference coded [ 2.14% Coverage]

Reference 1 - 2.14% Coverage

I: Are there any opportunities for you to review the implantation of hydrocele services and provide you feedback to improve the quality of services?
R: Yes, at the time when that community volunteer came to convince me to accept and talk to him about my condition. He gave some for a drink saying others hide for this condition.
