# Supplementary material for: Applying community health systems lenses to identify determinants of access to surgery among mobile & migrant populations with hydrocele in Zambia: A mixed methods assessment
Source: PLOS Glob Public Health. 2023 Jul 18;3(7):e0002145. doi: 10.1371/journal.pgph.0002145 (PMC10353788; doi:10.1371/journal.pgph.0002145)
Supplement: S3 File — Data collected and reported in the manuscript. (ZIP) [file pgph.0002145.s003.zip › S2. Datasets/Relational lens/Feedback mechanisms into formal health system.docx]

Files\\COMMUNITY HEALTH WORKER 1 - § 4 references coded [ 11.77% Coverage]

Reference 1 - 2.62% Coverage

= Okay, Let’s talk about this community here who provides services for hydrocele? who brings help.
R= Hydrocele here we have a provider if you refers a patient if found and us also we refer if we found one in the community, we also had someone who was our leader.
I= which leader
R= Chileshe
I= He was under what?
R= University
I= University of Zambia
R= Yes
I = Okay
R= He uses to come and hold meetings and he monitors what we were doing and monitors the books and the way we are working.

Reference 2 - 2.87% Coverage

R= Yes like people from the health district, they use to help so when they go, we use to go through as well.
I= Okay, are there some people who use to come and see how this program is moving.
R= Yes there where.
I=Who are those
R= The same from the university and other from the hospital and people from the boma.
I= How come people from the boma use to come twice twice?
R= They use to come monthly if not then quarterly they come.
I= What about these from the university
R= University then they use to come quarterly.
I= Quarterly
R= Yes

Reference 3 - 2.40% Coverage

I = do you have some where it is written and shows you the guide, the number of people you have found and those who have been operated on, do you have that?
R= we just used to write in the books
I= in books
R= yes
I= okay
R= In the note books we write we find this one was write we bring that person here and then is referred, the only had some papers.
I = So do you have those books and did they use to help.
R= Yes it uses to help us
I= how was it use to help you?

Reference 4 - 3.88% Coverage

I = so the same question use to remind you.
R= yes, that’s the some that use the remind us that on such a day should send 2 or 3 people for operation.
I= the same book, use to help you
R= yes it used to help us
I= in which way
R= it use to remind me that on this day this person is supposed to go, then I go and talk to other person.
I= the same books did there use to show you people who have renew received any help but you did find them
R= those we found?
I= yes, but never received any help
R= us when we go that aside when we come back I have to go back and see that person, so that person will tell me that they operated on him and if I send him and go back visit said no in failed then I will know then I will write that this person never went.

Files\\COMMUNITY HEALTH WORKER 2 - § 2 references coded [ 7.15% Coverage]

Reference 1 - 4.36% Coverage

I= Okay do you get any information that you send to the district like data sheet?
R= Information from the community we do get and when we get information from the community not in writing but just through the phone, we community that here we have found this problem like this, so them are supposed to keep that information.
I= do they have it?
R= I think so but am not sure because we have never meet and sit so that we look at hat problem, information may be reaching them but there’s nothing that they do.
I= okay so you have not used the data that you send.
R= no
I= Not yet
R= yes, for myself I haven’t used them.
I= okay but do you think if its there if can help
R= yes it can so that we can inform the funders so that the program can go forward.

Reference 2 - 2.79% Coverage

I= Okay do you have another recommendation at the provincial level
R= To add on here in our community we should just have a person to looking or guiding us in our community, but us the CHW we report to the provincial and I don’t know because may there is to much work to be done, we just need one person directly, like last time we had Chileshe and we started with him and things where just okay. So the province should just give us a person per district so that call him direct

Files\\HEALTH PROVIDER - § 4 references coded [ 15.82% Coverage]

Reference 1 - 2.37% Coverage

I= Were you given an opportunity to give the feedback how they were implementing the program?
R=yes
I= yes why do you say so?
R=because people where responding to the information they were giving other coming back to say no I was like and this when these people came to me I was able to go to the hospital and I was able to go to the hospital and I was worked on right now am okay

Reference 2 - 5.85% Coverage

I=so these two have to be where?
R=what?
I= two per what?
R= per facility, okay that will depend with the population I think like for facility two people will be enough for other facility I don’t know if they want three or what?
I= local health workers or community workers do their utilize the guidelines in helping the implementation of hydrocele?
R=yes
I=what kind of manual do they use?
R=I have just forgotten the name but they here at our at our facility.
I=okay so what is the guidelines in the guidelines in that ones. There is how to prevent how, I can’t remember but there are some things like how to prevent it and the severity.
R= do they do so how do they make sure that these guidelines are followed?
I=they do, so how do they make sure that these guidelines are followed?
R= may you ask the clients questions
I = is there anyone who is supervising that the guidelines are followed?
R= yes
I= who is thatone?
R= Martha
I=who is Martha?

Reference 3 - 4.33% Coverage

I=okay let’s move to the next question, do you have data for hydrocele patients that you collect for the service and compile together?
R=yes we do
I=you do?
R=yes
I=okay what is your role in data collection?
R= just to know how many people have hydrocele and how many do not have.
I= how would you describe the quality of the data that you collect?
R= the data that we collect is just okay
I=it’s okay?
R=yes
I= why do you say it’s okay?
R= because the way the reason I say it’s okay when we collect data from the very person so they give us the collect information
I=okay
R= yes
I= so do you like often analyzed used data to find the delivery of hydrocele services?
R=yes
I=how do you use it?
R=the same way

Reference 4 - 3.28% Coverage

I= Would you know how patients who have received and not received treatment or any kind of hydrocele services?
R=yes because those who receive treatment like here we can say surgery, we write the names and those have not received we write the names so that we know how many were worked on and how many remained.
I=so can you be able to know the needs from the communities from the same data.
R=yes
I=it can show you?
R=yes
I= why do you say so?
R=just from the clients that we have can show us the need of the people and what they want.

Files\\HEALTH WORKER 1 - § 1 reference coded [ 2.65% Coverage]

Reference 1 - 2.65% Coverage

I= okay, that’s good, is there meetings that are held for hydrocele in plemenatation services like here to the health post specifically for hydrocele.
R= specifically for hydrocele, because think when, the first round come when we were going to see how many patients we have who has this in our catchment area, finally we had to sit down when the project was ending.
I= okay,
R= we have to sit down
I= when was that?
R= it should sometimes, earlier this year
I= early this year?
R= yes
I= okay
R= I cannot specifically se the month but I think in one of this months to conclude what was going on and to see how many have been helped, and who still need but I think we were fortunately we were blessed, most of the people that we had on our register for us were attended to.

Files\\HEALTH WORKER 2 - § 2 references coded [ 9.65% Coverage]

Reference 1 - 7.64% Coverage

I= Do you collect any data on how hydrocele service are being provided or utilized mere at the catchments are?
R= yes we have the data for them.
I= you do
R= yes
I= so what is your role in that data.
R= data collection
I= yes
R= when we collect the same data we send to the district health office and mostly our role is to provide IEC, health education to the community.
I= okay
R= yes
I= yes
I= so is there any effective monitoring and feedback that help you to collect the relevant information
R= 80% thing when they go in field they identify then they carried back, we get those people that have been identified, send that data to the district health office, they give us the feedback we go back to the community and communicate to those people who identified them.
I= so who do you describe the quality of the data that you collect?
R= the data is effective
I= is effective?
R= yes
I= okay, how often us used to inform the delivery of hydrocele.
R= I think quarterly
I= Quarterly
R= Yes
I= Okay, do you show it’s effective?
R= yes its effective but not very effective like what I said we need more people for it to be effective because they are just few people doing data collection.
I= so like are able to use the same data to determine those that are not receiving the hydrocele services.
R= Yes
I= you are able
R= Yes
I= Why do you say saw?
R= Because when the same data is collected we are able to take note of the patients that are coming to access the health services and we use the same data to notice those people who haven’t come to access the health services.

Reference 2 - 2.01% Coverage

I= Okay, the same data is able to tell you the need of the community,
R= Yes
I= Why do you say so?
R= When they collect the same data if will tell us how many people are affected and why are they affected and have they not accessed the health services, when data is collected it will show long that people has been with hydrocele disease and also where haven’t they not come to the facility, so it’s able to do that.

Files\\Head Clinical Care LDH - § 1 reference coded [ 3.98% Coverage]

Reference 1 - 3.98% Coverage

I: Is there any data that is collected for hydrocele services within the district?
R: We have the numbers of hydrocele that have been handled, those that have recovered and in case of any complications, we have the book where we record.
I: Is there any role you do with the collection of data?
R: No.
I: That information that you have when the data is collected on hydrocele cases, is it often analysed and used to make an informed decision on hydrocele services?
R: It is not used, we just collect and store for future use.
I: Have you been able to use the data to identify communities that are not receiving hydrocele services?
R: No, not really, we depend on CHW to inform us.
I: How do you identify gaps with regards to hydrocele services?
R: That one is very difficult to see the gaps, maybe the only indicator is starting to see a reduction in the number of patients coming through.

Files\\IDI - CBV - Kasinsa - § 1 reference coded [ 5.49% Coverage]

Reference 1 - 5.49% Coverage

: Is there an effective way of monitoring and feedback mechanism in place to help you collect data?
R: I had something that was providing guidance. But I no longer use it.
I: Do you think that was relevant?
R: Yes.
I: How?
R: Because it used to provide some guidance.
I: Was the data you collected utilised or used to inform the delivery of hydrocele service in the community?
R: It was helping, because when we collect data in the community, we would present it to the CO which helped him to make some informed decision
I: Did the data help you identify other communities where patients did not receive hydrocele services?
R: Yes.
I: How?
R: The time I used to collect data, after some days, you find that they start coming on their own here, as in those who never wanted to know.
I: Did the data help you identify the community needs?
R: Yes.
I: How? To what extent do you think it was able to help you know the needs of the community?
R: To a larger extent. We were able ask how the services were delivered.

Files\\IDI - CHW - Mangelengele - § 4 references coded [ 12.92% Coverage]

Reference 1 - 2.82% Coverage

I: Are there any opportunities given to you and community members including patients, to review the implementation of hydrocele services?
R: I can say yes, on the last study we had, time was there to see how the program was going and see if it was the way we wanted or not.
I: Were you and other members able to provide your feedback or give input on the implementation of these hydrocele services?
R: Yes, during the last study, most of them we spoke to said that the programme was successful and people were happy. Though this happened at the end.

Reference 2 - 4.36% Coverage

I: Alright, so what kind of opportunities where there? Did you go for a meeting or it was done door to door?
R: It was a meeting and some were called at once and where being interviewed about what they think about the whole program.
I: How often were the meetings held?
R: I have forgotten.
I: Can you say it was once in a year, once in a month or once after 3 months?
R: Not after 3 months, there were stages. After collection of data, you sit, aggregate the data and agree on the sending time then you sit and discuss for how many have received the data. After sending, you discuss how many were successful in sending how many remained, that was how it was. It was done stage by stage.
I: Do you think the suggestions and views given by the stakeholders were used to implement the hydrocele programs in the district?
R: Others have been used but others no.

Reference 3 - 4.40% Coverage

I: Where you given guidance on how you were supposed to collect this data?
R: Yes, when you do not understand, you ask and they guide you.
I: Okay, how do you describe the quality of data that you collected?
R: The data was quiet good I think there were no problems since it was used when starting the project up to the end.
I: So was the data you collected utilised?
R: The data was used because after everything finished, they used it to see that there were a number of people that remained with the conditions and they followed up to work on the people that remained.
I: Did the same data help you to make an informed decision regarding which people did not receive the service for hydrocele
R: Yes, it helped because we knew that there are people with the problems and the reason they failed to go for help was luck of knowledge and expenses of the hospital.

Reference 4 - 1.34% Coverage

I: To what extent do you feel that the data you collected is able to inform you on the needs of the community?
R: The data has helped us to know which one received and did not receive the services. And it also helps to know what the patients’ needs like pain killers

Files\\IDI - Com Leader - Chitope - § 1 reference coded [ 2.59% Coverage]

Reference 1 - 2.59% Coverage

I: Has there been a time when you asked to provide information that can be used to improve the implementation of hydrocele services within your community?
R: Yes. Here at this facility they called for me as well as at Katondwe mission hospital. What I told them is that when hydrocele patients come to the facility they should be attended to fast treating their cases as emergence because if they are delayed and they see a lot of women at the facility they feel uncomfortable and want to go back immediately and once they have gone home it becomes very difficult to bring them back to the hospital. That is what I shared with them

Files\\IDI - Com Leader - M - Kasinsa - § 3 references coded [ 8.02% Coverage]

Reference 1 - 1.07% Coverage

I: Why do you say you are satisfied?
R: Everyone who access the hydrocele services appreciate the services when they have fully recovered saying in fact we were just wasting time delaying to access hydrocele services.

Reference 2 - 2.35% Coverage

I: Is there any example you would give that makes patients to appreciate.
R: At first patients used to think they will be discriminated when they visit the clinic. When patients go to the clinic they find no discrimination against people with hydrocele they will access the services. Then after undergoing surgery they are the ones who are encouraging their friends to go and access hydrocele services and admitting that they were just wasting time delaying going to the hospital.

Reference 3 - 4.59% Coverage

I: Has there been a time when you or any other community member were given an opportunity to review the implementation of hydrocele services and be able to provide your input in order to improve the quality of service for hydrocele?
R: Yes. I have had that chance.
I: What opportunity was available for you?
R: While it was a meeting which was taking place and the district in 2019 and we gave contributions as a members of that meeting and not really an individual.
I: Where such meeting used to provide feedback on the implementation of hydrocele services?
R: Yes I would say so because that is where the organizers were able to get information on how they will go about with hydrocele services in the community.
I: How often are these meetings held?
R: Well, such meetings come once in a year.
I: What challenges that can make you it difficult for you to provide feedback or input on how to improve hydrocele service?
R: I don’t have such a challenge.

Files\\IDI health provider Chitope - § 2 references coded [ 5.63% Coverage]

Reference 1 - 3.49% Coverage

I: Are there opportunities were the stakeholders review the implementation of the hydrocele service?
R: It is rare that we do have reviews for these programs. Even at facility level, most of the times we only remember about these patients when they come to seek help for the hydrocele services, or possibly come for some other illnesses like Malaria or ART, that is when we remember this is one of our hydrocele patients. For some of the staff, they cannot know that someone is a hydrocele patient. I think we need a register where we have names for these clients and even have some follow up programmes for them so that at least they can be actively monitored. Sorry to say that I think we have neglected them and the only time we follow them is when we have programmes to deal with lymphatic Filariasis and elephantiasis and at times when the University of Zambia comes to follow up on their study I think that is when they followed up closely.

Reference 2 - 2.14% Coverage

I: Do you collect any data on how hydrocele services are being utilised at your health facility?
R: The only time data was collected was during the study by the University of Zambia, after that, nothing.
I: Did you take part in the data collection?
R: No, I did not take part, I was part of the team that was managing patients who came back after surgery and after they come back, they come for feedback and review.
I: Do you have access to any data that was collected?
R: The only data we have is the number of patients for hydrocele and possibly those who received a kind of service.

Files\\IDI health provider Mandombe - § 1 reference coded [ 7.70% Coverage]

Reference 1 - 7.70% Coverage

I: Do you collect data on hydrocele services?
R: Yes.
I: At the facility and district?
R: Yes, we do collect at both.
I: What is your role during data collection process?
R: We involve CBVs to go and write down names who are eligible for this and we tell them how to go about it, through this, identification comes in for who has hydrocele. Based on that data, we follow up as health workers, if they say they have identified 6 people with hydrocele, we test them and refer them to the district. If there is need for any financial help, we inform the district hospital.
I: Do you supervise or take part in the data collection process?
R: We send the CBVs, they do data collection and we supervise them in the community, we usually monitor them to make sure there are no errors during data collection.
I: How would you describe the quality of data that you collect?
R: The data collected is ok. We do analyse the data, when the cases are coming in, we analyse and after analysing it, we make an informed decision.
I: Have you been able to use the data to identify communities that have not been receiving hydrocele services?
R: Yes we do. We collect the data and they say at that particular household, the patient needs to access the medical services. We health honkers follow up to make sure they access the services. If they are hesitant in accessing the services, we explain to them about the importance of accessing the hydrocele services. So when the people who are doing data collection reports to the facility, it is our turn as health workers to make a follow up on the facilities.
I: To what extent do you think the data you collect helps you identify the needs of the communities?
R: Not too large and not too small.
I: In short, do you feel that data you collect helps you know the needs of the community?
R: Yes because when the data comes we sit down and analyse it, so from there it 100% gives us the needs of the community. We are able to tell that the community lacks this and this and we come up with informed decisions.
