# Supplementary material for: Applying community health systems lenses to identify determinants of access to surgery among mobile & migrant populations with hydrocele in Zambia: A mixed methods assessment
Source: PLOS Glob Public Health. 2023 Jul 18;3(7):e0002145. doi: 10.1371/journal.pgph.0002145 (PMC10353788; doi:10.1371/journal.pgph.0002145)
Supplement: S3 File — Data collected and reported in the manuscript. (ZIP) [file pgph.0002145.s003.zip › S2. Datasets/Relational lens/Biography and geography of kowledge production.docx]

Files\\COMMUNITY LEADER 1 - § 1 reference coded [ 3.28% Coverage]

Reference 1 - 3.28% Coverage

I = When you look at these people in community, do you think they influence to look at this program of hydrocele?
R = There are some who do not know when you have the hydrocele they used to remove the one which is for reproduction so people were scared but today people have learnt that they only remove water from the testicles and not removing everything and people now are willing to go for an operation.
