# Supplementary material for: Applying community health systems lenses to identify determinants of access to surgery among mobile & migrant populations with hydrocele in Zambia: A mixed methods assessment
Source: PLOS Glob Public Health. 2023 Jul 18;3(7):e0002145. doi: 10.1371/journal.pgph.0002145 (PMC10353788; doi:10.1371/journal.pgph.0002145)
Supplement: S3 File — Data collected and reported in the manuscript. (ZIP) [file pgph.0002145.s003.zip › S2. Datasets/Relational lens/Political economy, feminist and intersectionality.docx]

Files\\Head Clinical Care LDH - § 2 references coded [ 2.86% Coverage]

Reference 1 - 1.31% Coverage

: Do you think it could lack of proper legal documents for the migrants?
R: That is where another the problem comes in because coming from the border, they need to be given a pass and when coming to the hospital, they are supposed to say that they are foreigners and that could be another fear

Reference 2 - 1.55% Coverage

I: What of the fishermen or migrants, do they also participate?
R: Those, no, it is very difficult because the time we meet the migrants is very short because when you see them, within a short period, they are gone. For fishermen, themselves it is very difficult because all they believe is to be in water and do fishing to feed their families.
