# Supplementary material for: Applying community health systems lenses to identify determinants of access to surgery among mobile & migrant populations with hydrocele in Zambia: A mixed methods assessment
Source: PLOS Glob Public Health. 2023 Jul 18;3(7):e0002145. doi: 10.1371/journal.pgph.0002145 (PMC10353788; doi:10.1371/journal.pgph.0002145)
Supplement: S3 File — Data collected and reported in the manuscript. (ZIP) [file pgph.0002145.s003.zip › S2. Datasets/Relational lens/Power and power relations.docx]

Files\\COMMUNITY HEALTH WORKER 1 - § 1 reference coded [ 3.90% Coverage]

Reference 1 - 3.90% Coverage

R= yes guidelines some I know the some no they are difficult.
I= okay can you tell me those which you know?
R=some of the guide is that this person with hydrocele is a person whom they have to talk to nicely, there should be privacy and you should not tell others that this person is like that.
I = okay, what do you do so that these guidelines are followed?
R= you have to use them.
I= you are them
R= yes
I= how do you manage that these guidelines are followed.
R= I have to call that person we talk in privacy and whatever is discussed an not supporse to disclose the to anyone else. Is between is I will only tell the nurse because he/she is the one who known everything and the nurse she is the one who will you will pass through.

Files\\COMMUNITY HEALTH WORKER 2 - § 1 reference coded [ 4.51% Coverage]

Reference 1 - 4.51% Coverage

I= Okay, tell me the patients in this community who are suffering from hydrocele, how do they find any help
R= Help like going to the hospital
I= Yes going to the hospital? On this issue of hydrocele
R= Okay, these patients they find help through when we go to them as community workers and tell them so that they know why they get sick, that’s how they manage to get help, but on their own they have their own rules that holds them not to go, so they need to be pushed in order for them to go.
I= Can you tell me what are those traditional rules that they follow?
R= Those people they do not believe that they can be healed form the disease of hydrocele, they believe that those patients are bewitched, so most of them who want help they go to the traditional healer.

Files\\COMMUNITY LEADER 1 - § 2 references coded [ 4.19% Coverage]

Reference 1 - 1.27% Coverage

I = Do you have any challenges to the people in your community about hydrocele disease?.
R = It’s not difficult because am the headman I Can talk at anytime.

Reference 2 - 2.92% Coverage

I = Si I will request for your words of recommendation towards the programs of hydrocele at this facility level.
R = What is required is for them to continue with the program of hydrocele so that people can be helped and us headmen we shall continue encouraging people to go to the hospital for operations and checks these are the words of encourage I can give.

Files\\HEALTH WORKER 1 - § 2 references coded [ 6.29% Coverage]

Reference 1 - 3.28% Coverage

I= okay are there certain types like patients hydrocele patient that access these services more easily than others
R= yes
I= what are those types?
R= I think the younger ones.
I= the younger ones
R= yes I think the younger ones because they look at the productivity of their lives, if you look at who at the old ones I think they have been living with this program for a very long time and then attributed to traditional medicine, you are like because you did this you are like because of witchcraft, you like this because you slept with some body’s wife now some wants to get back to you, so I think before the public had that much information everything would be kept under laps, people wouldn’t want other people to know that this is the condition that they have, the younger ones because they are looking at their at their you know they family men, they need to provide for their families, they need the comfort and also the marital problems.

Reference 2 - 3.01% Coverage

I= so may be let’s look at the babble community here, I want who is involved in the hydrocele disease case.
R= we have our community health workers, of course we the families of the patients we also have the headmen.
I= okay
R= yes, because you cannot do any program, or attar where it is coming from without our traditional leaders, because for you to b able to enter that community you first need to go through them.
I= okay, so can you tell me specific roles that they play for example the community leader, that I may be concerned with this disease, what do they do in helping.
R= they are the ones that Convey the meetings on our behalf
I= okay the meetings
R=they Convey the meetings so that we are able to spread the information is it the mass drug administration, they know what when people know that it has the blessings of the traditional leader s then it is safe.

Files\\Head Clinical Care LDH - § 2 references coded [ 4.74% Coverage]

Reference 1 - 2.25% Coverage

I: Any recommendations at local political structures?
R: Those have to be involved because if there is political will, then that is when things can run. If it is the Councillor, town Chairperson is well informed because these are the people who the people on the ground and if the information may come from civic leaders, it may be easy for people follow what they always say. So these are the key people to tackle so that when they come they will talk to the district and the district will take it up.

Reference 2 - 2.48% Coverage

I: Any recommendations on how we can integrate this at National level?
R: I think the advocacy has to come from the University, you know like school of Public health, has the power, resources, information to go and convince those at national level to say this is a problem that we have identified at this district and this is the recommendation we have for this district. That will be much easier, like us here, we have information but we do not use it. But with the school itself with its resource, knowledge and skills it can help us implement this.
