# Supplementary material for: Applying community health systems lenses to identify determinants of access to surgery among mobile & migrant populations with hydrocele in Zambia: A mixed methods assessment
Source: PLOS Glob Public Health. 2023 Jul 18;3(7):e0002145. doi: 10.1371/journal.pgph.0002145 (PMC10353788; doi:10.1371/journal.pgph.0002145)
Supplement: Attachment — Submitted filename: Responses to reviewers- Lungwa CHS.docx [file pgph.0002145.s004.docx]

**Journal Requirements:**

1. Please include additional information regarding the survey or questionnaire used in the study and ensure that you have provided sufficient details that others could replicate the analyses. For instance, if you developed a questionnaire as part of this study and it is not under a copyright more restrictive than CC-BY, please include a copy, in both the original language and English, as Supporting Information.

We have provided the all data collection tools as supplementary materials.

2. Please amend your detailed Financial Disclosure statement. This is published with the article. It must therefore be completed in full sentences and contain the exact wording you wish to be published.
   a. State the initials, alongside each funding source, of each author to receive each grant.

We have amended the funding section to indicate that Joseph Mumba Zulu (JMZ) was the recipient of the grant.

b. State what role the funders took in the study. If the funders had no role in your study, please state: “The funders had no role in study design, data collection and analysis, decision to publish, or preparation of the manuscript.”

We have included the description provided above highlighting that the funders did not play a role in any of the study processes.

c. If any authors received a salary from any of your funders, please state which authors and which funders.

None of the authors received a salary through the grant.

3. We have noticed that you have uploaded Supporting Information files, but you have not included a list of legends. Please add a full list of legends for your Supporting Information files after the references list.

The legends for all the supporting materials has been included.

**Review Comments to the Author**

4. On a broad level, the paper is quite focused on mobile/migrant health but only around 20% of those sampled were migrants. The authors did say about 50% travel seasonally so this could perhaps just be stressed or better set up for that as the focused population in the discussion. I would argue that migrant populations (from other countries) and mobile populations (Zambians) might face different circumstances, though.

Thank you very much for the comment. Though only 20% of the survey participants are migrants, mobile populations are also represented in the survey either as fishermen or farmers who travel seasonally across the borders. Further, aside from the survey participants, qualitative data was also collected from the both the migrants and the mobile populations and integrated in the results section where specific references are made to barriers/facilitators affecting each populations but consolidated using community health system lenses. In targeting mobile and migrant populations we were interested in looking at community members who cannot be served by static health services.

5. As an aside, the discussion section alluded that some surveys were done in other countries. Is this true? If so, this should be better outlined in the methods section (see specific suggestions below on more information sampling requested).

In the methods section, we highlight that the community health workers not only went household to household but went into neighboring communities. In Zambia, community boundaries go beyond the national boundaries with neighboring countries such Zimbabwe and Mozambique. So one finds that a community can span two countries with patients accessing health services in the Zambian health facilities even if they are coming from Mozambique or Zimbabwe. Since Community health workers play are critical role in health service delivery in the country, they become familiar with patients across borders and are able to identify them and link them to care.

6. Similarly, most people in this study were unemployed, yet the discussion focused on fisherman as a focused group. Could the authors focus their discussion more on the population studied?

In the study, we categorized the fishermen as a mobile population because they have to go away on fishing expeditions for long periods of time usually crossing over to Mozambique and Zimbabwe which hampers the extent to which they are able to access services.

7. A very minor percentage of patients underwent surgery. I wonder if the authors survey included more information on why that could complement the qualitative findings.

In the results, we wanted to reflect an integrated joint display of the barriers and facilitators drawn from the quantitative and qualitative data but aligned to the community health systems lens. Therefore the placement of the survey responses to the barriers may be interspersed throughout the results rather than at single point. For instance, Lines 280-282, 325-326 or 357-361.

8. I do wonder the relationship—or lack there of—of traditional healers and other stakeholders in the therapeutic landscape. Stacey Langwick writes a lot about this in Tanzania. Would recommend the authors to review some of her work.
   CHWs have complex identities. Mohamed Yunus Rafiq writes about this in Tanzania. Would recommend the following study: https://pubmed.ncbi.nlm.nih.gov/31638989/
   Rafiq also writes about use of religious leaders in public health campaigns. Would caution the authors to simply add that as a proposed solution without a more in-depth conversation for something that is complex on multiple levels: <https://pubmed.ncbi.nlm.nih.gov/34915242/>

Thank you for drawing our attention to the work being conducted by both Stacey Langwick and Mohamed Yunus Rafiq particular as they relate to the relational lens as well as the collective action lens of the Community health systems. Though our study did not go deep into the collective action lens we hope to explore it further in our upcoming work.

9. Was discrimination addressed on the survey?

The study procedures were conducted in an ethical manner that limited opportunities for discrimination. We used local community health workers to identify patients, we contacted the patients at home or in other areas where they felt comfortable being interviewed.

10. The paper has multiple grammatical errors in the form of incomplete sentences.

We have reviewed the article and corrected the grammatical errors and incomplete sentences.

11. Surgical camps are not a solution if people fear surgery or going for diagnosis. It’s a treatment that will not work if no one comes. Can the authors better lay out what this might look like as a solution or what it has looked like elsewhere?

Our suggestion is for the surgical camps to be placed in fishing camps particularly targeting fishermen as they would bringing the services closer to them. We found that when they travel on fishing expeditions they have limited access to health services and this can be a potential service delivery point.

**Line by Line Comments**

**Abstract**

12. Recommend labeling sections of abstract (e.g. background, methods, results, conclusion)

The sections have been labeled (lines 8, 13, 17, 26)

13. Line 25-27 the sentence “The absence of cross border collaborative structures that can support the continuity of care for patients moving across the three countries” is not a complete sentence. Recommend revising.

We have revised the sentence such that it reads “The absence of cross border collaborative structures limits the continuity of care for patients moving across the three countries.”

14. Line 29, need a comma after “play”

We have added a comma.

**Introduction**

15. Line 52 – need comma after “higher level hospitals”

We have included a comma as per the provided suggestion.

16. Lines 56-61. I don’t completely follow the authors explanation of how universal uptake of a treatment (such as hydrocelectomy) leads to health inequities, particularly point number three that delayed care leads to unequal treatment outcomes (this part makes sense) but then this has an impact on people’s social positions? How is that related to the intervention of surgery itself rather than this simply being a manifestation of the complex sociopolitical and economic circumstances within which people live?

In our previous publications on health beliefs and health seeing practices in the district, we found that having hydrocele is seen as a sign of high social standing with patients being considered wise and respected elders. So they often delay getting surgery because it means that their social standing in the community is likely to change afterwards. This is what we were trying to highlight in that particular sentence. As such we have expanded the sentence to reflect this.

17. The authors have provided an EXCELLENT discussion of the unique challenges faced by migrant/mobile populations. I would only ask them to also include language barriers as a major impediment to health, particularly in the context of surgery. This is very relevant to the topic at hand because sometimes distinguishing between an incarcerated hernia and a hydrocele can be difficult and good history taking is critical.

Thank you very much for this comment which represents a common challenge we have experienced in our study site particularly among Community health workers as they do case identification and referral. We have included a sentence to reflect this.

18. Line 95-97 – incomplete sentence.

We have rephrased the sentence for greater clarity.

19. More distinction on the programmatic and critical lens might be useful.

We have included an additional description of the critical lens from the guiding publication.

20. It might be helpful to provide more background specifically on hydrocele treatment in Zambia and provide the reader with background epidemiology and patterns of disease and treatment.

We have included a statement of the burden of LF in Zambia. One of the challenges in estimating the burden of existing hydrocele cases is that a morbidity mapping is conducted as part of MDA campaigns which underestimates the number of patients available. For instance in the most recent mapping, cases in the study district are estimated to be 60 yet our study found over 400 patients. Cases are typically identified passively and health facilities which tend to have health providers who have not been appropriately trained to conduct the surgeries.

**Methods**

21. Line 112 – replace the word “with” with the word “and” so it is a complete sentence.

The word with has been replaced.

22. It would benefit the reader to understand, particularly for hydrocele treatment, at what level of the health system a hydrocelectomy would normally be offered? Is the lowest level that it is offered the district level hospital?

This statement has been included “Hydrocele surgery is typically provided at the district hospital after referral from the lower level health centres.

23. How many total households were visited to identify the 438 patients? How were households selected? Random sampling? Cluster sampling?
    The authors should provide more detail on their sampling methods. How were original individuals chosen? How was sample size determined?

The household level mapping was conducted by Community health workers. Each Community health worker has a number of households that they cover within their health facility catchment area. During the data collection visited each household under their jurisdiction they also went to specific areas where the mobile and migrant populations are found. As such we did not sample but conducted complete enumeration.

24. What online server was used?

We did not use any online server. The data was collected through paper-based questionnaires and the data entered into Excel before being exported to STATA for analysis.

25. Is the survey tool available to be published as supplementary material?

The survey and interview guides have been included in the supplementary materials.

26. Were the qualitative interviews done in English? IF not, what language was used? Who and how were they translated?

The qualitative interviews were done in English and various languages. We have added a statement on the same in the article. The research assistants were multilingual and the collected the data and did the transcription.

27. It seems like the authors used primarily deductive coding with the proposed frameworks. Can the authors further specify why or how they used inductive coding?

We have outlined that we used a deductive approach based on the community health system lens.

28. Line 175 – not a complete sentence.

The sentence has been rephrased.

**Results/Discussion**

29. Line 185 – is this average age a mean or median? Can the authors provide standard error or deviation (if mean) or IQR if median and they want to provide the average? Otherwise would recommend just referring to the table.

We have removed the sentence on the average age and left the subsequent statements that describe the information in the table.

30. Line 192 – Write 79% instead to be consistent with the other sentences. If authors want to avoid starting sentence with a number, consider revising the order of the sentence or simply combining with previous one.

We have made adjustments to the sentence to ensure consistency.

31. Line 233-234 – I don’t follow the reason provided for patients not getting surgery if they knew it is available

The main theme that we are trying to highlight in that section is that even with high levels of awareness, low levels of acceptability poses a challenge to the uptake of the surgeries. This low level of acceptability is driven by the absence of cross border structures, patients under estimating the severity of their cases and the need for care as well as myths about the disease.

32. Line 235 – why could migrants not access surgery? Several reasons were hypothesized in the introduction, but it is unclear if these reasons were elicited in the survey for migrants specifically.

In the results, we wanted to reflect an integrated joint display of the barriers and facilitators drawn from the quantitative and qualitative data but aligned to the community health systems lens. Therefore the placement of the survey responses to the barriers has been interspersed throughout the results rather than at single point. For instance, Lines 280-282, 325-326 or 357-361.

33. Line 264 - is there a sample quote to back up this claim?

We have included a quote from one of the Community health workers.

34. Line 284 – if patients from outside Zambia pay higher fee, would recommend authors provide more details on this in the intro since there is a big focus on migrants in their paper but not enough background on this in the introduction section.

A statement on the difference in the fees paid by locals and migrants has been included in the introduction.

35. The whole section seems to partly focus on implementation and how patients are not included but what interventions have really been proposed or used besides community health leaders trying to encourage people?

Granted that our work is an implementation science study, our goal is to identify implementation determinants and match them to implementation interventions which are also referred to as implementation strategies within the field. The implementation strategies that have been provided as table 3 are the proposed interventions. We have described them using an existing taxonomy for reporting implementation strategies to ensure that they are coherent with existing literature.

36. Table 3 impossible to read. Recommend putting on landscape and reformatting.

We have used the reporting guidelines for implementation interventions/ Implementation strategies to structure table 3. Our concern in shifting the table is that it may not speak to implementation research literature and recommendations on enhanced replicability of such strategies if extensive changes are made. In addition, the journal requires the table to be in portrait mode during the submission and review phases.
