# Supplementary material for: Applying community health systems lenses to identify determinants of access to surgery among mobile & migrant populations with hydrocele in Zambia: A mixed methods assessment
Source: PLOS Glob Public Health. 2023 Jul 18;3(7):e0002145. doi: 10.1371/journal.pgph.0002145 (PMC10353788; doi:10.1371/journal.pgph.0002145)
Supplement: Attachment — Submitted filename: Reviewers comments.docx [file pgph.0002145.s005.docx]

**Author responses to general and reviewer’s comments**

1. Please review your reference list to ensure that it is complete and correct. If you have cited papers that have been retracted, please include the rationale for doing so in the manuscript text, or remove these references and replace them with relevant current references. Any changes to the reference list should be mentioned in the rebuttal letter that accompanies your revised manuscript. If you need to cite a retracted article, indicate the article’s retracted status in the References list and also include a citation and full reference for the retraction notice.

We have carefully reviewed all the articles that have been included in the paper and none of them have been retracted.

2. Please include additional information regarding the survey or questionnaire used in the study and ensure that you have provided sufficient details that others could replicate the analyses. For instance, if you developed a questionnaire as part of this study and it is not under a copyright more restrictive than CC-BY, please include a copy, in both the original language and English, as Supporting Information."

A description of the survey methods and the qualitative interview procedures have been described in the article and all the data collection tools that were used have been provided in the supporting information.

3. Reviewer #1: Recommend paper move forward with acceptance. Commend the authors for their time spent on the review. I would still ask that the additional information responded to in the comments on methods and some communities spanning two countries find its way into the final manuscript.

Thank you very much for your review and helpful comments. We have included a statement in the methods that highlights that data collection was done in communities whose boundaries cross international borders i.e. Mozambique and Zimbabwe. Further, we have also indicated that as a border district, movement is commo because people farm, fish, trade or go to see their families. So the community boundaries go beyond the geographic/international boundaries.

4. I would also still ask the authors to comment one or two sentences to acknowledge that even if you bring surgical camps closer to a community---this does not necessarily translate to more people utilizing health services. Appreciate the opportunity to review an important contribution to this field and commend the authors on a well thought out study and well written manuscript.

Thank you very much for your review and helpful comments. We have added a statement in the discussion highlighting that availability of services does not always mean their utilisation.
